# Trimetallic Nanozyme‐Embedded Smart Hydrogel Enables NIR‐Controlled Bacterial Killing and Oxidative Stress Alleviation

**DOI:** 10.1002/advs.202512875

**Published:** 2025-10-03

**Authors:** Zehui Xiao, Jiangli Cao, Jifeng Liu, Zhiyong Song, Ting Du, Xinjun Du

**Affiliations:** ^1^ State Key Laboratory of Food Nutrition and Safety College of Food Science and Engineering Tianjin University of Science and Technology Tianjin 300457 P. R. China; ^2^ College of Science Huazhong Agricultural University Wuhan 430070 P. R. China

**Keywords:** dynamic covalent hydrogel, mild PTT, multiple enzyme‐like activities, self‐switching, wound healing

## Abstract

Nanozyme‐based antibacterial therapy is limited by inefficient single‐component nanozymes and complex infection microenvironments. A mild near infrared‐I (NIR‐I) photothermal‐enhanced nanozyme catalytic system is developed using polymyxin B‐modified trimetallic nanoparticles (AuMnCu) embedded in a smart hydrogel (AMCB‐FTB) formed by 3‐formylphenylboronic acid (FPBA), tobramycin (TOB), and tannic acid (TA). The AuMnCu nanozymes exhibit self‐switching multi‐enzyme activity, generating ROS for bacterial killing in non‐NIR mode while scavenging ROS and producing oxygen post‐disinfection to alleviate oxidative stress and hypoxia, promoting wound healing. Under NIR‐I irradiation, mild hyperthermia (≈44.3 °C) further boosts catalytic activity, enhancing sterilization. The AMCB‐FTB hydrogel is injectable, pH‐/temperature‐responsive, and releases tobramycin/tannic acid in acidic infection microenvironments, synergizing with photothermal therapy (PTT) and nanozyme activity for potent antibacterial effects. In vitro and in vivo studies confirm AMCB‐FTB's programmable antibacterial, anti‐inflammatory, and pro‐regenerative functions via microenvironment self‐regulation. RNA sequencing analysis confirm that AMCB‐FTB combined with NIR disrupts bacterial energy metabolism, protein synthesis, and lipid pathways, effectively suppressing survival, motility, biofilm formation, and virulence. This work reports a microenvironment‐responsive hydrogel with enzyme‐mimetic ROS modulation properties, providing a novel pathway to develop thermal‐enhanced catalytic materials for refractory diabetic wounds and infectious diseases.

## Graphical abstract

A microenvironment‐responsive hydrogel is developed incorporating polymyxin B‐modified AuMnCu nanozymes and dual drugs (tannic acid and tobramycin). At infection sites, it generates ROS, releases drugs responsively, and enhances mild photothermal action to disrupt bacterial redox balance. After disinfection, it self‐switches to scavenge ROS, reduce hypoxia, and stimulate angiogenesis, enabling precise and programmed repair of infected wounds.

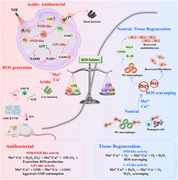

## 1 Introduction

Infectious wounds continue to pose a significant challenge to global healthcare systems, often aggravated by bacterial resistance, excessive inflammatory activity, and disruptions in the tissue regeneration processes.^[^ ^1^, ^2^ ^]^ Reactive oxygen species (ROS), including hydroxyl radicals (•OH), superoxide anions (•O_2_^−^), and hydrogen peroxide (H_2_O_2_), exert a paradoxical influence during wound healing. While they serve as antimicrobial agents in the early inflammatory stage, their uncontrolled accumulation can lead to oxidative stress, persistent inflammation, and delayed tissue recovery.^[^ ^3^, ^4^, ^5^ ^]^ Excessive or prolonged inflammation exacerbates tissue injury and hinders fibroblast recruitment and function, which are essential for extracellular matrix deposition, collagen remodeling, and wound closure. Despite advances in clinical interventions, conventional treatments such as antibiotics and single‐function nanozymes frequently lack the capacity to modulate ROS homeostasis in a stage‐specific manner throughout the healing cascade.

Nanozymes, engineered nanostructures that mimic natural enzyme functions, have garnered attention as robust candidates for ROS modulation, owing to their structural adaptability, functional diversity, and long‐term stability.^[^ ^6^, ^7^, ^8^ ^]^ For instance, nanozymes with peroxidase‐like (POD‐like) properties facilitate ROS generation to combat microbial infections, whereas those exhibiting superoxide dismutase (SOD‐like) or catalase (CAT‐like) activities function primarily to neutralize excess ROS, thereby alleviating oxidative stress.^[^ ^9^, ^10^ ^]^ However, effective wound repair requires more than antibacterial and antioxidant functions. The capacity to mitigate inflammation and promote fibroblast proliferation and migration is equally essential for accelerating granulation tissue formation and re‐epithelialization. Many current nanozymes operate in a fixed mode and lack the capability to dynamically alternate between ROS production and scavenging in response to the evolving microenvironment of the wound. This critical limitation highlights the urgent need for developing stimuli‐responsive nanozyme systems that can adaptively regulate their catalytic functions according to dynamic pathological demands.

Innovative strategies that integrate the sensing and precise control of the infection microenvironment can be employed to address this challenge. Although high levels of glutathione (GSH) are known to affect the catalytic efficiency of nanozymes, recent advances in photo‐responsive systems offer new solutions.^[^ ^11^, ^12^, ^13^ ^]^ One of the primary methods to enhance the nanozyme catalytic performance involves leveraging the Arrhenius principle, where elevated temperatures accelerate reaction rates and thereby amplify enzymatic activity.^[^ ^14^, ^15^ ^]^ Photothermal therapy (PTT) is effective in generating localized thermal therapy, which can overcome the temperature limitations associated with nanozyme‐catalyzed therapies. However, excessive operating temperatures (> 50 °C) may cause irreparable damage to healthy tissues. Therefore, mild thermotherapy enhances the catalytic activity of the nanozymes without harming healthy tissues. This synergistic strategy bridges the gap between static nanozymes and the dynamic wound microenvironment. Furthermore, it simultaneously regulates inflammation and stimulates fibroblast activity, laying the foundation for smart wound dressings that can autonomously adapt therapy.

**Scheme 1:**
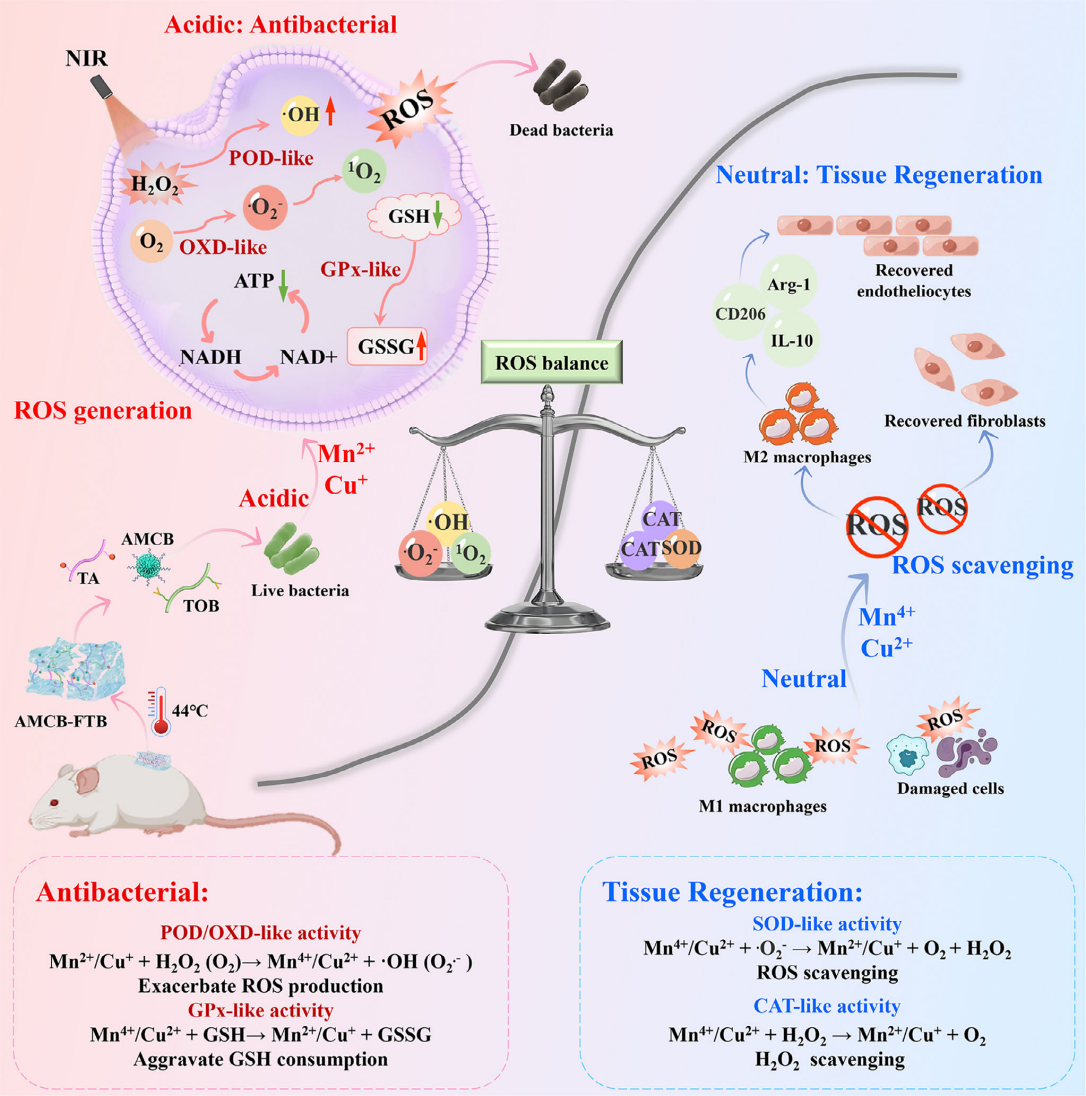
Bacteria‐responsive programmable hydrogels with a cascade reaction mechanism exhibiting self‐switching enzyme‐like activity.

In this study, a smart self‐activating on‐demand antimicrobial hydrogels responsive to the bacterial infection microenvironment was fabricated, which has the ability to remodel the regenerative microenvironment of wound infections to clear bacteria for wound recovery (**Scheme** **1**). We first constructed a trimetallic AuMnCu nanozyme (abbreviated as AMC) with intrinsic multi‐enzyme activity. Then, polymyxin B was modified on the surface of AMC based on the Au─S bond and amide bond (named as AMCB), which could enhance the targeting of the bacteria. Next, dynamic covalent hydrogels with aldehyde and boronic acid groups were prepared using 3‐formylphenylboronic acid (FPBA), tobramycin (TOB), and tannic acid (TA). Due to the presence of a large number of dynamic covalent bonds in the hydrogel system, the resulting hydrogel exhibits multi‐stimulus responsiveness and controlled drug release. More importantly, the mild photothermal effect of AMCB can break the intermolecular hydrogen bonds between small molecule building blocks, enabling not only the controlled release of “antimicrobial drugs” from the hydrogel but also facilitating catalytic nanozyme‐based therapeutics. In the pre‐infection stage, the released AMCB can precisely locate the bacteria. The mildly acidic pH favors electron transfer from Mn^2+^ and Cu^+^ to H_2_O_2_, enhancing the POD‐like catalytic activity of AMCB, thereby weakening the antioxidant defense mechanism and energy metabolism of the bacteria, leading to bacterial death. In contrast, the elevated pH in the post‐infection stage shifts the valence states toward a redox equilibrium (Mn^4+^/Cu^2+^), which favors SOD‐ and CAT‐like activities, thereby facilitating ROS scavenging and tissue repair. Furthermore, Cu^2+^ promotes angiogenesis by regulating vascular endothelial growth factor (VEGF) after eliminating infection.^[^ ^16^, ^17^ ^]^ RNA sequencing analysis results also demonstrated that AMCB‐FTB hydrogel combined with NIR treatment could severely inhibit bacterial survival, motility, biofilm formation, and virulence by interfering with bacterial energy metabolism, protein synthesis, and lipid pathways. Overall, the constructed composite hydrogels exhibited smart bacterial response, self‐activation, on‐demand antimicrobial properties, and promotion of tissue vascularization, providing an effective strategy for the efficient treatment of wound infection and healing.

## 2 Results and Discussion

### 2.1 Synthesis and Characterization of Au NPs, AMC, and AMCB

**Figure 1:**
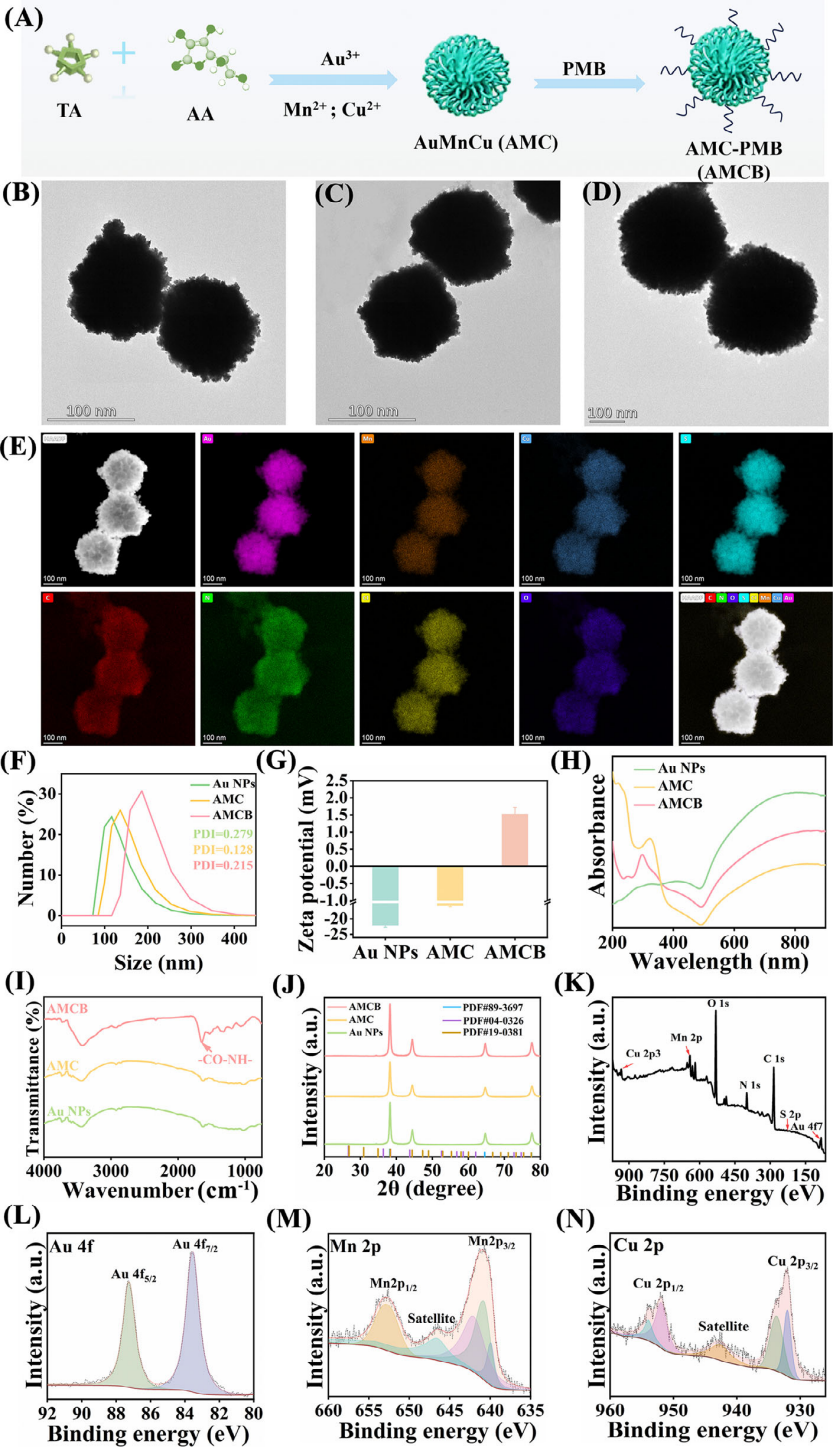
Characterization of AMCB. A) Preparation process of multifunctional acid‐responsive trimetallic nanozymes. TEM of Au NPs (B), AMC (C) and AMCB (D). E) Elemental mapping of AMCB. The average hydrodynamic diameters (F), zeta potentials (G), UV–vis absorption spectra (H), FTIR spectra (I), XRD patterns (J), of Au NPs, AMC and AMCB. XPS survey spectra (K) and high‐resolution XPS spectra of Au 4f (L), Mn 2p (M) and Cu 2p (N) orbits for AMCB.

**Figure** **1**A outlines the stepwise preparation of multifunctional acid‐responsive trimetallic nanozymes. Gold nanoparticles (Au NPs) were synthesized by one‐step reduction using tannic acid (TA) and ascorbic acid (AA) as reducing agents, and the successful formation of Au NPs was confirmed by transmission electron microscopy (TEM) (Figure 1B). After the removing of the supernatant, manganese (Mn^2+^) and copper (Cu^2+^) ions were introduced into the Au NPs solution, where they reacted with TA on the nanoparticle surfaces to form the trimetallic AuMnCu nanozyme (AMC) (Figure 1C). Subsequently, polymyxin B (PMB) was conjugated to the AMC through an amide reaction (AMCB, Figure 1D). The grafting rate of PMB was found to be 53.50% ± 7.86% (Figure S1, Supporting Information). The resulting AMCB nanoparticles exhibited a uniform sea urchin‐like morphology with a lattice spacing of 256.4 pm (Figure S2A,B, Supporting Information). Scanning transmission electron microscopy‐energy dispersive X‐ray spectroscopy (STEM‐EDX) and energy‐dispersive spectroscopy (EDS) further confirmed the elemental distribution of Au, Mn, Cu, S, C, N, O, and Cl within the AMCB structure (Figure 1E; Figure S2C, Supporting Information).

Dynamic light scattering (DLS) results indicated that the average hydrodynamic diameters of Au NPs, AMC, and AMCB were 116.1, 135.8, and 185.8 nm, which were consistent with the TEM results. The PDI was 0.279, 0.128 and 0.215 for Au NPs, AMC and AMCB, indicating that they have good dispersion (Figure 1F). Zeta potentials of Au NPs and AMC were −22.15 ± 0.59 mV and −15.76 ± 0.32 mV, respectively. After modification by PMB, AMCB with a positive charge of 1.52 ± 0.19 mV was formed (Figure 1G). Additionally, we monitored the changes in hydrodynamic diameter and surface charge of AMCB in different environments (containing water, DMEM, RPMI 1640 and simulated wound fluid (SWF)) over a 96 h period using DLS analysis. As shown in Figures S3 and S4 (Supporting Information), the hydrated particle size of AMCB remained ≈186 nm across various environments and time points, while the surface charge remained consistent. No obvious aggregation or charge neutralization is observed. This stability is beneficial for AMCB to maintain its catalytic performance in vivo. Figure 1H shows the UV–vis spectrum of AMCB, exhibiting a distinct and broad absorption band within the range of 500–900 nm. This feature facilitates effective photothermal heating under 808 nm laser irradiation. In FTIR analysis, the spectra of AMC and Au NPs are nearly identical due to the absence. AMCB exhibits a distinct amide bond absorption peak at 1645 cm^−1^, indicating the successful attachment of PMB onto the AMC surface (Figure 1I).^[^ ^18^ ^]^ Figure 1J shows the XRD patterns of Au NPs, AMC and AMCB. The characteristic peaks of Au NPs at 38.22°, 44.38°, 64.66° and 77.68° can be assigned to the [111], [200], [220], and [311] reflection plane of Au NPs (PDF#89‐3697). AMC exhibited two typical diffraction peaks at 38.27° and 44.37°, corresponding to the [111] and [200] planes of Cu (PDF#19‐0381), and the [111] and [050] planes of Mn (PDF#04‐0326), respectively. An additional peak at 77.48° was attributed to the [332] plane of Cu (PDF#19‐0381). No crystalline peaks were detected for PMB in the XRD pattern, therefore, the diffraction profiles of AMC and AMCB are consistent. The elemental composition and valence states of AMCB were characterized by X‐ray photoelectron spectroscopy (XPS). As shown in the survey spectrum (Figure 1K), five characteristic peaks corresponding to Cu 2p (933.1 eV), Mn 2p (641.1 eV), O 1s (531.1 eV), C 1s (285.1 eV), and Au 4f (84.1 eV) were identified. The C 1s spectrum (Figure S5A, Supporting Information) was deconvoluted into five component peaks located at 283.47, 284.16, 284.80, 285.61, and 287.67 eV. The O 1s spectrum (Figure S5B, Supporting Information) exhibited two distinct peaks at 532.14 and 530.83 eV, assigned to C═O and C─O bonds, respectively. In the Au 4f region (Figure 1L), the doublet at 83.54 and 87.24 eV corresponds to Au 4f_7/2_ and Au 4f_5/2_, respectively. The high‐resolution S 2p spectrum (Figure S5C, Supporting Information) displayed three peaks at binding energies of 165.36, 167.18, and 170.78 eV, attributed to atomic sulfur, S 2p_3/2_, and S 2p_1/2_, respectively.^[^ ^19^ ^]^ The Mn 2p spectrum (Figure 1M) showed major peaks at 640.79 eV (Mn 2p_3/2_) and 652.64 eV (Mn 2p_1/2_),^[^ ^20^ ^]^ along with satellite features at 642.02 and 646.57 eV characteristic of Mn^2+^. An additional peak observed at 639.90 eV was assigned to Mn(0).^[^ ^21^ ^]^ Quantitative analysis indicated that the peak area of Mn^2+^ is larger than that of Mn(0) (Figure 1M). The Cu 2p high‐resolution spectrum (Figure 1N) exhibited principal peaks at 932.02 eV (Cu 2p_3/2_) and 953.93 eV (Cu 2p_1/2_), consistent with Cu^2+^.^[^ ^22^ ^]^ A minor peak at 951.9 eV was attributed to Cu^+^. The satellite peaks at 942.56 and 933.72 eV further support the presence of Cu^2+^. The larger peak area of Cu^2+^ compared to Cu^+^ confirms that the dominant oxidation state of copper in AMCB is +2. The coexistence of Cu^2+^ and Mn^2+^ enables glutathione (GSH) depletion, thereby enhancing the efficacy of chemodynamic therapy (CDT). These XPS results confirm the successful synthesis of AMCB nanocomposites.

### 2.2 AMCB Enzyme‐Mimicking Activities under Acidic Conditions

**Figure 2:**
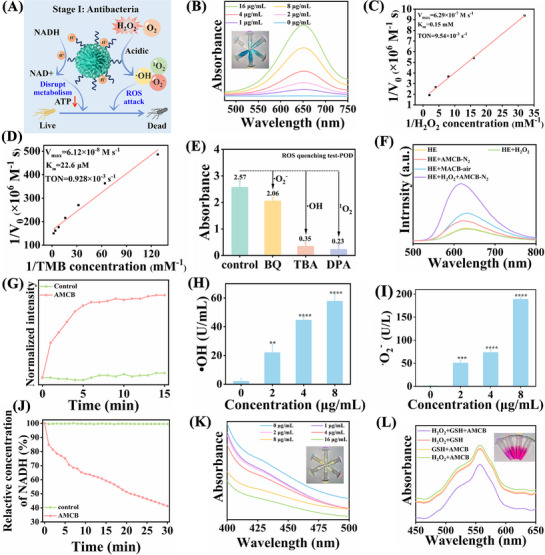
A) Schematic diagram of ROS generation and NADH oxidation under acidic conditions in AMCB. B) Absorption curves after reaction between AMCB and TMB at different concentrations (0–16 µg mL^−1^). C,D) Lineweaver‐Burk plots of AMCB for POD‐like activity. E) Free radical quenching data. F) HE probes detection of •O_2_^−^. G) Detection of ^1^O_2_ by 9,10‐diphenanthraquinone. H) •OH and I) •O_2_^−^ generation after treatment with different concentrations of AMCB (0–8 µg mL^−1^). Data are presented as mean ± SD (*n* = 3). Statistical significance was tested with one‐way ANOVA, **p < 0.01, ***p < 0.001, ****p < 0.0001. J) Time‐dependent curve of NADH oxidation after different treatments. K) Changes in absorbance at 412 nm after the reaction of different concentrations (0–16 µg mL^−1^) of AMCB with GSH and DTNB. L) Degradation of RhB by AMCB under different conditions.

The catalytic properties of AMCB under acidic conditions were investigated through in vitro assays, including POD‐like and NADH oxidase‐like activities (**Figure** **2**A). In Figure 2B, in the presence of a fixed concentration of H_2_O_2_ (50 µm), the color intensity increased with higher AMCB concentrations, indicating enhanced POD‐like activity.

To further evaluate the catalytic efficiency of AMCB, steady‐state catalytic analyses were performed. Michaelis‐Menten curves were used to determine key kinetic parameters, including the maximum reaction rate (V_max_), Michaelis constant (K_m_), and turnover number (TON, defined as the maximum number of substrate molecules converted per catalytic site per second). For H_2_O_2_, AMCB exhibited a V_max_ of 6.29 × 10^−7^ m s^−1^, a K_m_ of 0.15 mm, and a TON of 9.59 × 10^−3^ s^−1^ (Figure 2C; Figure S6A, Supporting Information). For TMB, the corresponding values were V_max_ = 6.12 × 10^−8^ m s^−1^, K_m_ = 22.6 µm, and TON = 0.928 × 10^−3^ s^−1^ (Figure 2D; Figure S6B, Supporting Information). These kinetic parameters were significantly superior to those of horseradish peroxidase (HRP), which has a V_max_ of 1.0 × 10^−7^ m s^−1^ and a K_m_ of 0.434 mm,^[^ ^23^ ^]^ underscoring the highly efficient POD‐like catalytic activity of AMCB toward H_2_O_2_. Further comparison with recently reported transition metal‐based nanozymes, such as metal oxides and metal nanoparticles (Tables S1 and S2, Supporting Information), revealed that AMCB outperforms these materials in terms of TON, V_max_, and K_m_ (Figure S7, Supporting Information), highlighting its superior ROS‐generating capacity. To identify the types of ROS produced by AMCB, hydroxyl radicals (•OH) were trapped using tert‐butanol (TBA), superoxide anions (•O_2_^−^) with p‐benzoquinone (BQ), and singlet oxygen (^1^O_2_) with 9,10‐diphenylanthracene (DPA). The results confirmed that the primary ROS produced were •OH and ^1^O_2_ (Figure 2E). Additional validation through hydroethidine (HE) and DPA assays further affirmed the generation of •O_2_^−^ and ^1^O_2_ (Figure 2F,G). Quantitative analysis using specific ROS detection kits also revealed that the concentrations of •OH and •O_2_^−^ increased proportionally with AMCB concentration (Figure 2H,I). Moreover, the POD‐like activity of AMCB were evaluated at different time points (0, 12, 24, 36, 48, 72, and 96 h) and under various environmental conditions (containing water, DMEM, RPMI 1640 and SWF). The results demonstrated well‐maintained catalytic efficiency, confirming the strong enzymatic stability even under conditions of high redox stress and elevated protein content (Figure S8, Supporting Information). These findings demonstrate the potential application of AMCB in treating bacterial infections.

To evaluate whether AMCB could catalyze the oxidation of NADH to NAD^+^, thereby disrupting the NADH/NAD^+^ balance in bacteria, the oxidation process was monitored using UV–vis spectroscopy. As shown in Figure 2J, the time‐dependent NADH oxidation curves under various treatments illustrate the dynamics of NADH depletion. These results demonstrate that AMCB significantly accelerates NADH oxidation compared to the control group, indicating pronounced NADH oxidase‐like activity and the capacity to perturb bacterial energy metabolism over time.

Bacterial infections upregulate GSH synthase activity, thereby promoting GSH synthesis and regeneration.^[^ ^24^ ^]^ However, excess GSH can neutralize ROS including •O_2_^−^, •OH, and H_2_O_2_, which may compromise the antibacterial efficacy of nanozymes.^[^ ^25^ ^]^ Notably, several studies have shown that metal nanozymes can deplete GSH by exhibiting glutathione peroxidase (GSH‐Px)‐like activity, leading to enhanced therapeutic outcomes.^[^ ^26^, ^27^ ^]^ To evaluate the GSH‐Px‐like properties of AMCB, we employed 5,5′‐dithiobis‐(2‐nitrobenzoic acid) (DTNB) as a probe to measure its GSH depletion capacity. DTNB reacts with GSH to form a stable yellow product, which exhibits a characteristic absorption peak at 412 nm, allowing spectrophotometric quantification.^[^ ^24^, ^28^ ^]^ As depicted in Figure 2K, treatment with AMCB (16 µg mL^−1^) for 10 min resulted in the consumption of 100 µm GSH, and the solution became nearly transparent. These results demonstrate that AMCB effectively depletes GSH via redox interactions involving Cu^2+^ and Mn^4+^, thereby enhancing the efficacy of ROS‐dependent CDT.

Furthermore, the effects of AMCB on bacterial oxidative stress were studied by simulating the bacterial infection microenvironment using H_2_O_2_ and GSH. The degradation of RhB was tested after adding AMCB, H_2_O_2_, and GSH to the RhB solution. The extent of RhB degradation reflects the level of ROS generated via the Fenton reaction. In Figure 2L, the most pronounced degradation of RhB occurred in the presence of AMCB + H_2_O_2_ + GSH, while significantly less degradation was observed with AMCB + GSH, AMCB + H_2_O_2_, or GSH + H_2_O_2_ alone. In summary, AMCB exhibits exceptional multifunctional enzyme‐like catalytic activity under acidic conditions. It exhibits highly efficient POD‐like and OXD‐like activities, catalyzing the decomposition of H_2_O_2_ to generate •OH, •O_2_^−^ and ^1^O_2_. Additionally, AMCB shows notable NADH oxidase‐like activity, accelerating NADH consumption and disrupting bacterial energy metabolism. It also possesses strong GSH‐Px‐like activity, enabling rapid depletion of GSH. These synergistic effects (ROS burst, NADH/NAD^+^ imbalance, GSH depletion) form the foundation of AMCB's potent antibacterial activity.

### 2.3 Preparation and Characterization of AMCB‐FTB Hydrogel

**Figure 3:**
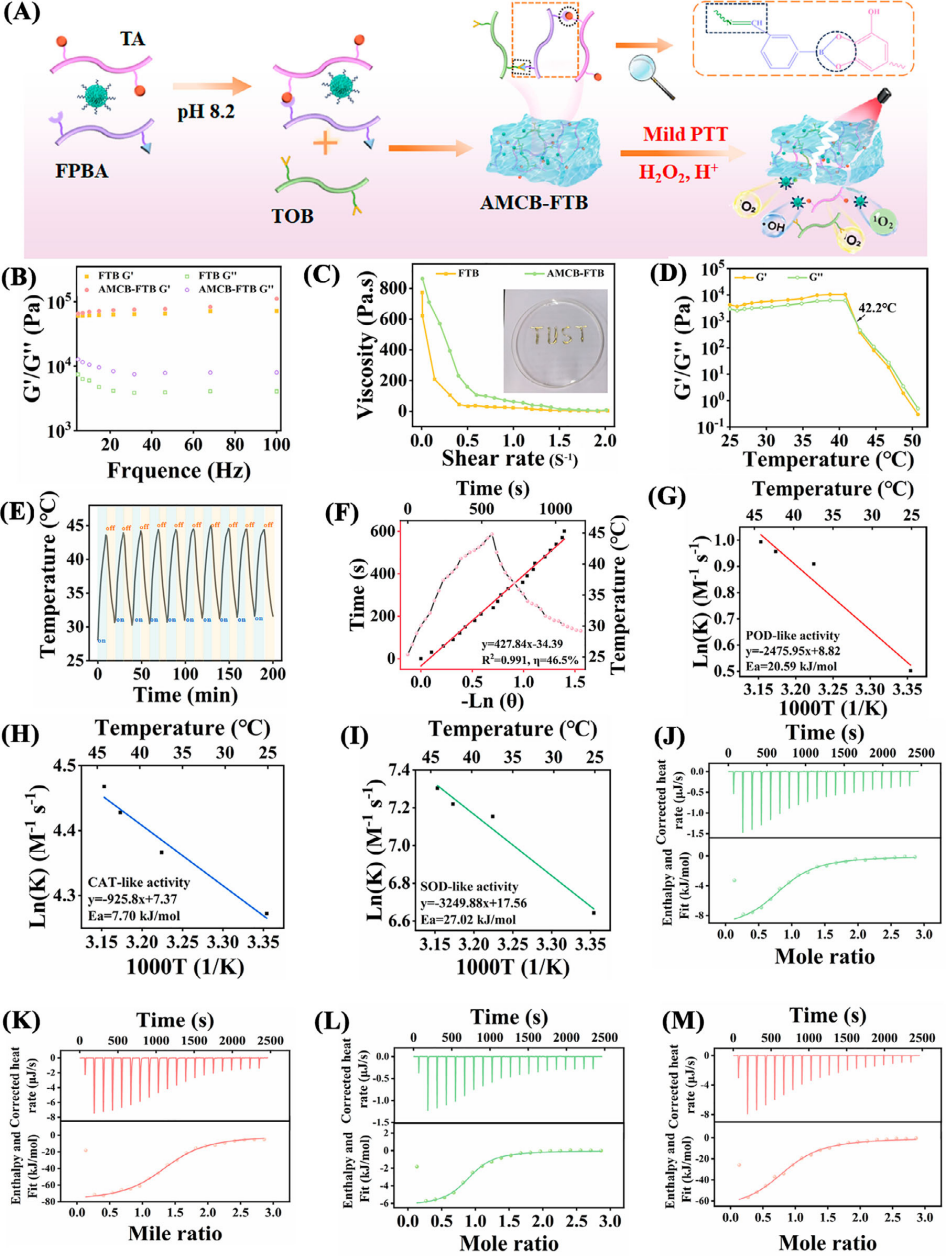
A) Preparation process of AMCB‐FTB. B) Storage moduli (G′) and loss moduli (G″) of FTB and AMCB‐FTB. C) The shear‐thinning properties of FTB and AMCB‐FTB. D) The rheological properties of AMCB‐FTB vary with temperature. E) Photothermal stability of AMCB‐FTB after 10 cycles of laser on/off irradiation. F) Correlation between cooling time and ‐lnθ and photothermal conversion efficiency of AMCB‐FTB. The activation energy (Ea) for POD‐like activity (G), CAT‐like activity (H) and SOD‐like (I) activity by Arrhenius plots (ln(k) vs 1000/T). Isothermal titration calorimetry (ITC) analysis of the interaction between nanozymes and their substrates under NIR_(−)_ and NIR_(+)_ conditions. Binding profiles for POD‐like activity under NIR_(−)_ (J) and NIR_(+)_ (K) conditions. Binding profiles for CAT‐like activity under NIR_(−)_ (L) and NIR_(+)_ (M) conditions.

**Figure** **3**A illustrates the synthetic steps of the multifunctional hydrogels. TOB was conjugated directly with TA using 3‐formylphenylboronic acid (3‐FPBA) to prepare the FTB and AMCB‐FTB hydrogels. TA is a naturally derived polyphenolic structure featuring numerous reactive groups on its surface. This structure enables rapid reaction with boronic acid groups, forming dynamic boron ester bonds that respond to temperature, acidic pH, and hydrogen peroxide.^[^ ^29^, ^30^, ^31^ ^]^ Rheological analyses confirmed the successful formation of both FTB and AMCB‐FTB hydrogels. The intersection of the loss modulus (G″) and storage modulus (G′) curves indicated the gel formation time of FTB and AMCB‐FTB is 60 s (Figure S9A, Supporting Information). Frequency sweep measurements showed that G′ exceeded G″ across the tested range, confirming the solid‐like gel behavior of the hydrogels (Figure 3B). Under increasing shear rates, the shear stress initially increased, then decreased, and eventually plateaued. The yield stresses of FTB and AMCB‐FTB were determined to be 283.8 and 465.5 Pa, respectively (Figure S9B, Supporting Information). Both hydrogels also demonstrated pronounced shear‐thinning behavior, as illustrated by the smooth extrusion of the material through a needle and the subsequent formation of the “TUST” pattern, visually confirming their injectability (Figure 3C). More importantly, AMCB‐FTB exhibits significant temperature sensitivity. As temperature increases, both storage modulus (G′) and loss modulus (G″) decrease significantly. A gel‐to‐sol transition occurs when the temperature exceeds 42.2 °C, indicated by G′ falling below G″ (Figure 3D). Consistently, visual and thermal imaging records confirmed that AMCB‐FTB dissolved at 44 °C (Figure S9E, Supporting Information). Next, the photothermal performance of AMCB‐FTB was further systematically investigated. Under 808 nm laser irradiation (0.8 W cm^−2^), the temperature of AMCB‐FTB increased significantly by ≈ 44.3 °C until the temperature reached steady state (10 min). In contrast, the temperatures of the PBS and FTB remained almost unchanged (Figure S9C, Supporting Information). Based on the heating‐cooling curve, the photothermal conversion efficiency of AMCB‐FTB was calculated to be 46.5% (Figure 3F), which is higher than that of Au NPs (34.10%, Figure S10, Supporting Information), AMC (37.47%, Figure S11, Supporting Information), and AMCB (41.12%, Figure S12, Supporting Information). Moreover, after 10 cycles of heating‐cooling, AMCB‐FTB continued to exhibit a strong photothermal effect, demonstrating its high photothermal stability (Figure 3E). The photothermal performance of AMCB‐FTB is positively correlated with the power density of the 808 nm laser (Figure S9D, Supporting Information). All these results indicate that AMCB‐FTB holds great potential for efficient photothermal therapy.

To validate the enhancement of catalytic activity induced by NIR‐mediated mild photothermal effect (∼44.3 °C), we determined the enzyme kinetic parameters (K_m_, V_max_) of AMCB at different temperatures (25, 37, 42, and 44 °C) and verified the temperature‐dependent enhancement via Arrhenius fitting. The results are shown in Figure S13 (Supporting Information). For TMB (POD‐like activity), the Michaelis constant (K_m_) decreased from 22.6 µm at 25 °C to 8.47 µm at 44 °C, while the maximum reaction rate (V_max_) increased from 6.12 × 10^−8^ to 1.08 × 10^−7^ m s^−1^ over the same temperature range (Figure S13D,G,J,M, Supporting Information). For H_2_O_2_ (CAT‐like activity), a similar trend was observed: K_m_ decreased from 0.73 mm at 25 °C to 0.46 mm at 44 °C, while V_max_ increased from 2.86 × 10^−7^ to 3.48 × 10^−7^ m s^−1^ (Figure S13E,H,K,N, Supporting Information). For pyrogallol (SOD‐like activity), V_max_ increased from 3.07 × 10^−6^ m s^−1^ at 25 °C to 5.94 × 10^−6^ m s^−1^ at 44 °C, accompanied by a reduction in K_m_ from 112.85 to 61.62 µm (Figure S13F,I,L,O, Supporting Information). Furthermore, we constructed Arrhenius plots (ln(k) vs 1000/T). The calculated activation energies (Ea) were 20.59 kJ mol^−1^ for POD‐like activity, 7.70 kJ mol^−1^ for CAT‐like activity, and 27.02 kJ mol^−1^ for SOD‐like activity (Figure 3G–I), consistent with temperature‐dependent enhancement of AMCB's catalytic activity as described by the Arrhenius equation.

To elucidate the effect of NIR irradiation on the substrate‐binding behavior of the nanozyme, we performed isothermal titration calorimetry (ITC) to quantify the relevant thermodynamic parameters both in the absence (NIR_(‐)_) and presence (NIR_(+)_) of irradiation. As depicted in Figure 3J–M and Figure S9G (Supporting Information), NIR irradiation markedly increased the binding affinity between the nanozymes and their respective substrates. For instance, in the case of POD‐like activity, the association constant (K_a_) increased from 3.60 × 10^5^ to 4.78 × 10^5^ m ^−1^, while the dissociation constant (Kd) decreased from 2.78 × 10^−6^ to 2.09 × 10^−6^ m. A similar enhancement was observed for CAT‐like activity, with K_a_ increasing from 2.67 × 10^5^ to 3.19 × 10^5^ m ^−1^ and Kd decreasing accordingly. Further thermodynamic analysis revealed that the binding process was enthalpy‐driven under both conditions, and NIR irradiation resulted in more negative ΔH values, which decreased from −9.775 to −79.25 kJ mol^−1^ for POD‐like binding and from −6.297 to −68.55 kJ mol^−1^ for CAT‐like binding, suggesting strengthened molecular interactions. This was accompanied by a more negative change in Gibbs free energy (ΔG), reflecting an increase in the spontaneity of substrate binding upon irradiation.^[^ ^32^ ^]^ These results demonstrate that NIR irradiation markedly improves both the substrate‐binding affinity and catalytic thermodynamics of the AMCB, thereby enhancing its catalytic performance under photothermal stimulation.

### 2.4 Antibacterial Activity of AMCB‐FTB

**Figure 4:**
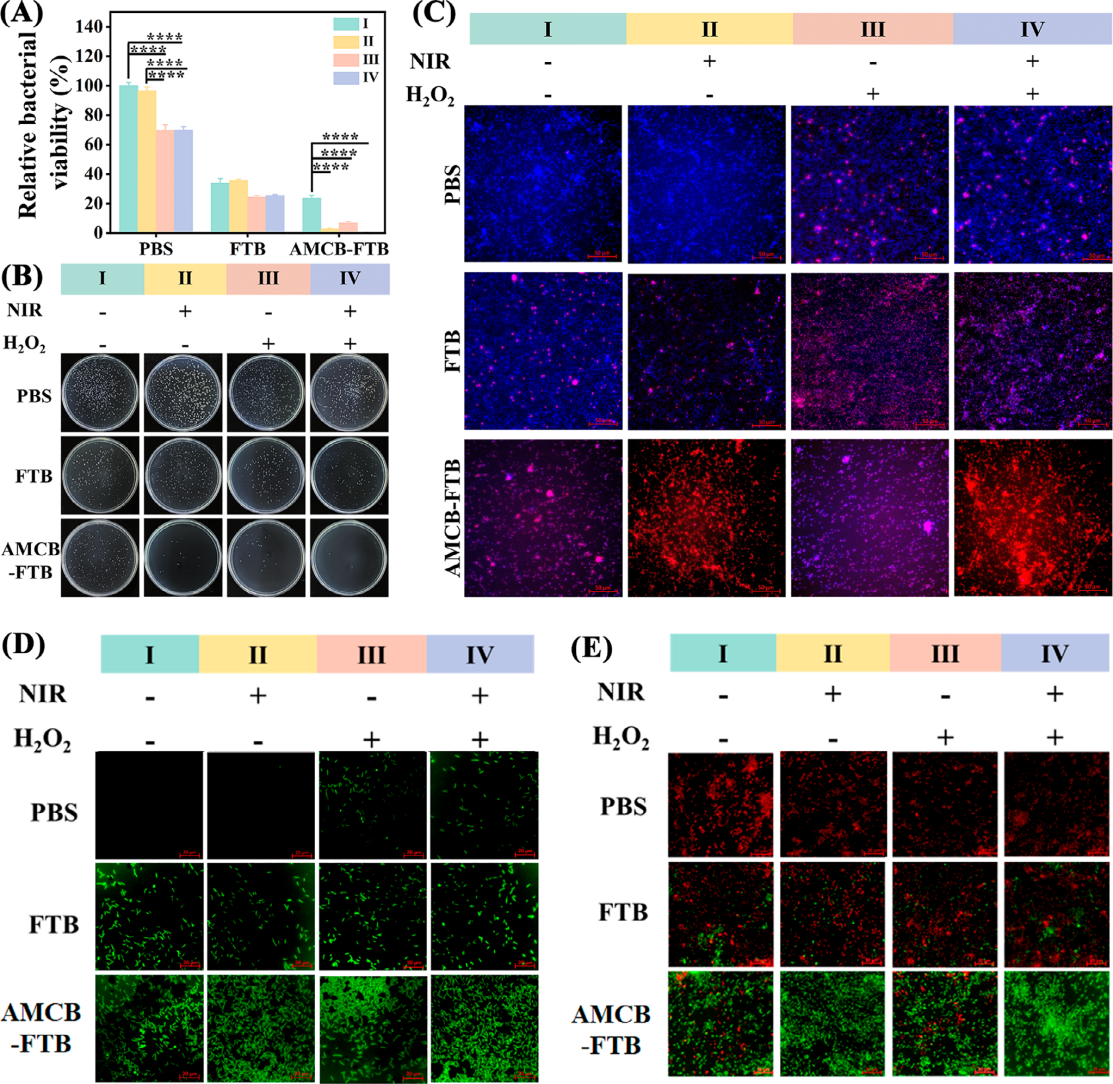
In vitro antibacterial activity of AMCB‐FTB. A) The corresponding relative bacterial viability of *P. aeruginosa*. B) Colonies growth of *P. aeruginosa* after different treatments on agar plates. C) Live/dead bacterial cells staining images of *P. aeruginosa* after different treatments. D) Fluorescence images of *P. aeruginosa* stained with DCFH‐DA after different treatments. E) Membrane potentials of *P. aeruginosa* after different treatments were stained with the fluorescent dye DiOC_2_(3). I: Treatments without NIR and H_2_O_2_. II: Treatments with NIR but without H_2_O_2_. III: Treatments without NIR but with H_2_O_2_. IV: Treatments with NIR and H_2_O_2_. Data are presented as mean ± SD (*n* = 3). Statistical significance was tested with two‐way ANOVA, ****p < 0.0001.

After demonstrating the ability of AMCB to generate ROS effectively in an acidic environment, we systematically evaluated its antibacterial activity. First, we focus on the pathogens represented by *Pseudomonas aeruginosa* (*P. aeruginosa*) and *Escherichia coli* (*E. coli*). Polymyxin B is a cyclic cationic antimicrobial peptide that can specifically bind to lipopolysaccharides in the cell walls of Gram‐negative bacteria, thereby targeting and binding bacteria.^[^ ^33^, ^34^, ^35^ ^]^ The inhibitory activity of FTB and AMCB‐FTB against *P. aeruginosa* and *E. coli* under different treatments were evaluated by plate coating method. In **Figures** **4**A,B and S14A,B (Supporting Information), the bactericidal activity of the pure H_2_O_2_ group (400 µm, Group III) was negligible. In contrast, the inhibition rates of AMCB‐FTB + H_2_O_2_ + NIR (Group IV) against *P. aeruginosa* and *E. coli* were 99.95% and 99.97%, respectively. This treatment group also produced the largest inhibition zones, with diameters of 30.78 mm for *P. aeruginosa* and 44.48 mm for *E. coli* (Figure S15, Supporting Information). These findings suggest that the combined ROS generation and photothermal effect of AMCB‐FTB under H_2_O_2_ and NIR activation make its antimicrobial effect superior to that of AMC (Figure S16, Supporting Information) and AMCB (Figure S17, Supporting Information). Additionally, comparative experiments were conducted to evaluate the antibacterial activity of Au NPs, AuMn, and AMCB. As shown in Figure S18 (Supporting Information), the AMCB group exhibited the most significant antibacterial activity. These results highlight the synergistic effect of Au, Mn and Cu, which collectively endow AMCB with enhanced catalytic and bactericidal capabilities compared to monometallic or bimetallic systems. To visualize the antibacterial effect of AMCB‐FTB, live/dead bacterial staining was performed using 4′,6‐diamidino‐2‐phenylindole (DAPI) and propidium iodide (PI). As shown in Figure 4C and Figure S14C (Supporting Information), bacteria treated with AMCB‐FTB + NIR + H_2_O_2_ emitted strong red fluorescence, demonstrating substantial membrane disruption and increased permeability induced by the combined PTT and CDT therapy. Overall, these results confirm that AMCB‐FTB exhibits excellent in vitro antibacterial efficacy under synergistic PTT and CDT activation facilitated by NIR laser and H_2_O_2_.

Bacterial membrane potential, which refers to the electrical potential difference across the bacterial membrane, is essential for maintaining vital cellular processes. To investigate the membrane properties under different treatments, we used the fluorescent probe 3,3′‐diethoxycarbocyanine iodide (DiOC_2_(3)) to monitor changes in membrane potential. At concentrations below 100 nm, DiOC_2_(3) accumulates in membranes with intact potential and emits red fluorescence. Disruption of the membrane potential leads to a reduction in red fluorescence and a concomitant increase in green emission. In Figure 4E and Figure S14E (Supporting Information), no green fluorescence was observed in the PBS group or the PBS + H_2_O_2_ group, while the AMCB‐FTB, AMCB‐FTB + H_2_O_2_, AMCB‐FTB + NIR and AMCB‐FTB + NIR + H_2_O_2_ groups produced a large amount of green fluorescence, indicating that the membrane potential decreased significantly and the membrane was depolarized and damaged. To further assess the ability of AMCB‐FTB to generate ROS within bacterial cells, 2′,7′‐dichlorofluorescein diacetate (DCFH‐DA) was used as a fluorescence‐labelled probe, which is oxidized to green‐fluorescent DCF in the presence of ROS. Figure 4D and Figure S14D (Supporting Information) show that both FTB and AMCB‐FTB treatments induced green fluorescence, with the most intense signal observed in AMCB‐FTB + NIR + H_2_O_2_ group. These results demonstrate that AMCB‐FTB can effectively produce ROS in the presence of H_2_O_2_, contributing to its strong antibacterial potential.

Notably, the acidic microenvironment resulting from bacterial colonization and metabolism can trigger the responsive release of antimicrobial agents from AMCB‐FTB, thereby enhancing bacterial killing. Additionally, localized heating of AMCB‐FTB accelerates the release rate of TOB and TA, further enhancing the antimicrobial efficacy of the hydrogel. Based on the standard curves of TA (Figure S19, Supporting Information) and TOB (Figure S20, Supporting Information), the cumulative release profiles of TOB and TA from both FTB and AMCB‐FTB exhibited clear H_2_O_2_‐ and temperature‐responsive behavior (Figure S21A,D, Supporting Information). The in vitro antibacterial effects of TA (0–500 µg mL^−1^) and TOB (0–25 µg L^−1^) were also evaluated. The results of Figure S21B,E (Supporting Information) show that the inhibitory effects of TA and TOB on bacteria are dose‐dependent. At a concentration of TOB was 25 µg L^−1^, the inhibitory effects on *P. aeruginosa* and *E. coli* were 39.97% and 40.11%, respectively (Figure S21B,C, Supporting Information). Similarly, TA (500 µg mL^−1^) exhibited inhibition rates of 37.59% against *P. aeruginosa* and 43.18% against *E. coli* (Figure S21E,F, Supporting Information). These results underscore the crucial contribution of both TA and TOB to the overall antimicrobial performance of AMCB‐FTB.

**Figure 5:**
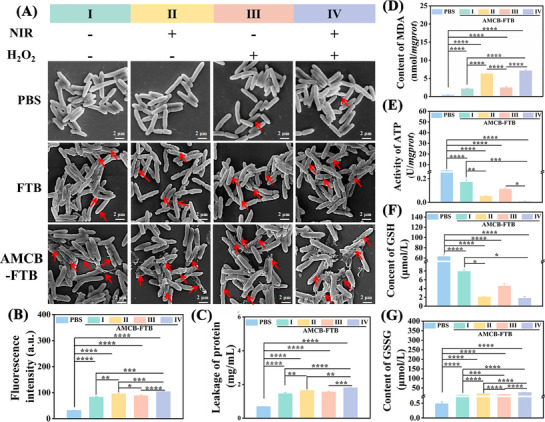
A) SEM images of *P. aeruginosa* after different treatments. Membrane permeability (B) and protein leakage (C) of *P. aeruginosa* after different treatments. The content of MDA (D), GSH (F) and GSSG (G) and activity of ATP (E) of *P. aeruginosa* after different treatments. I: Treatments without NIR and H_2_O_2_. II: Treatments with NIR but without H_2_O_2_. III: Treatments without NIR but with H_2_O_2_. IV: Treatments with NIR and H_2_O_2_. Data are presented as mean ± SD (*n* = 3). Statistical significance was tested with one‐way ANOVA, *p < 0.05, **p < 0.01, ***p < 0.001, ****p < 0.0001.

Further, the effect of different treatments on the integrity and morphology of bacterial membrane were analyzed by SEM (**Figure** **5**A; Figure S22A, Supporting Information). In the absence of NIR irradiation, both *P. aeruginosa* and *E. coli* treated with PBS maintained normal morphology with smooth surfaces and intact cell membranes. In the FTB group, the surfaces of *P. aeruginosa* and *E. coli* showed slight wrinkles. In contrast, bacteria treated with AMCB‐FTB, especially in combination with NIR irradiation and H_2_O_2_, exhibited pronounced membrane crumpling and disruption, accompanied by leakage of cellular contents. These SEM observations confirm that the combined treatment of AMCB‐FTB with NIR and H_2_O_2_ effectively kills bacteria by compromising their cell membrane integrity. Additionally, changes in bacterial membrane permeability were assessed using the fluorescent probe 1‐N‐phenylnaphthylamine (NPN). The intact outer membrane of Gram‐negative bacteria acts as a permeability barrier that excludes hydrophobic compounds such as NPN. Upon membrane damage, NPN can penetrate the phospholipid layer and produce a fluorescent signal.^[^ ^36^ ^]^ The experiment demonstrated that treatment with AMCB‐FTB significantly increased the membrane permeability of both *P. aeruginosa* and *E. coli* (Figure 5B; Figure S22B, Supporting Information). The amount of protein leakage from bacteria after treatment was determined using a protein concentration assay kit (Solarbio, Beijing). As expected, protein leakage in the AMCB‐FIB + H_2_O_2_ + NIR‐treated group also increased significantly (Figure 5C; Figure S22C, Supporting Information). The degree of lipid peroxidation in bacterial cell membranes was evaluated based on changes in malondialdehyde (MDA) content.^[^ ^37^, ^38^ ^]^ MDA quantification (Figure 5D; Figure S22D, Supporting Information) revealed a marked enhancement in lipid peroxidation following treatment with AMCB‐FTB + H_2_O_2_ + NIR. Adenosine triphosphate (ATP) serves as a critical molecule in cellular energy metabolism. A reduction in bacterial ATP levels can adversely affect growth, metabolic activity, and viability. As shown in Figure 5E and Figure S22E (Supporting Information), AMCB treatment led to a significant decrease in ATP content, indicating impaired bacterial survival.^[^ ^39^, ^40^ ^]^ Similarly, a pronounced increase in the ratio of oxidized glutathione (GSSG) to reduced GSH was observed (Figure 5F,G; Figure S22F,G, Supporting Information), reflecting GSH depletion and disruption of the GSSG/GSH redox balance.^[^ ^41^, ^42^ ^]^ These results demonstrate that the combination of AMCB with H_2_O_2_ and NIR induces bacterial destruction through multiple mechanisms: damaging the cell membrane, increasing membrane permeability, inducing protein leakage, promoting lipid peroxidation, depleting GSH, disturbing redox homeostasis, and compromising the cellular energy supply system.

### 2.5 Anti‐Biofilm of AMCB‐FTB in Vitro

**Figure 6:**
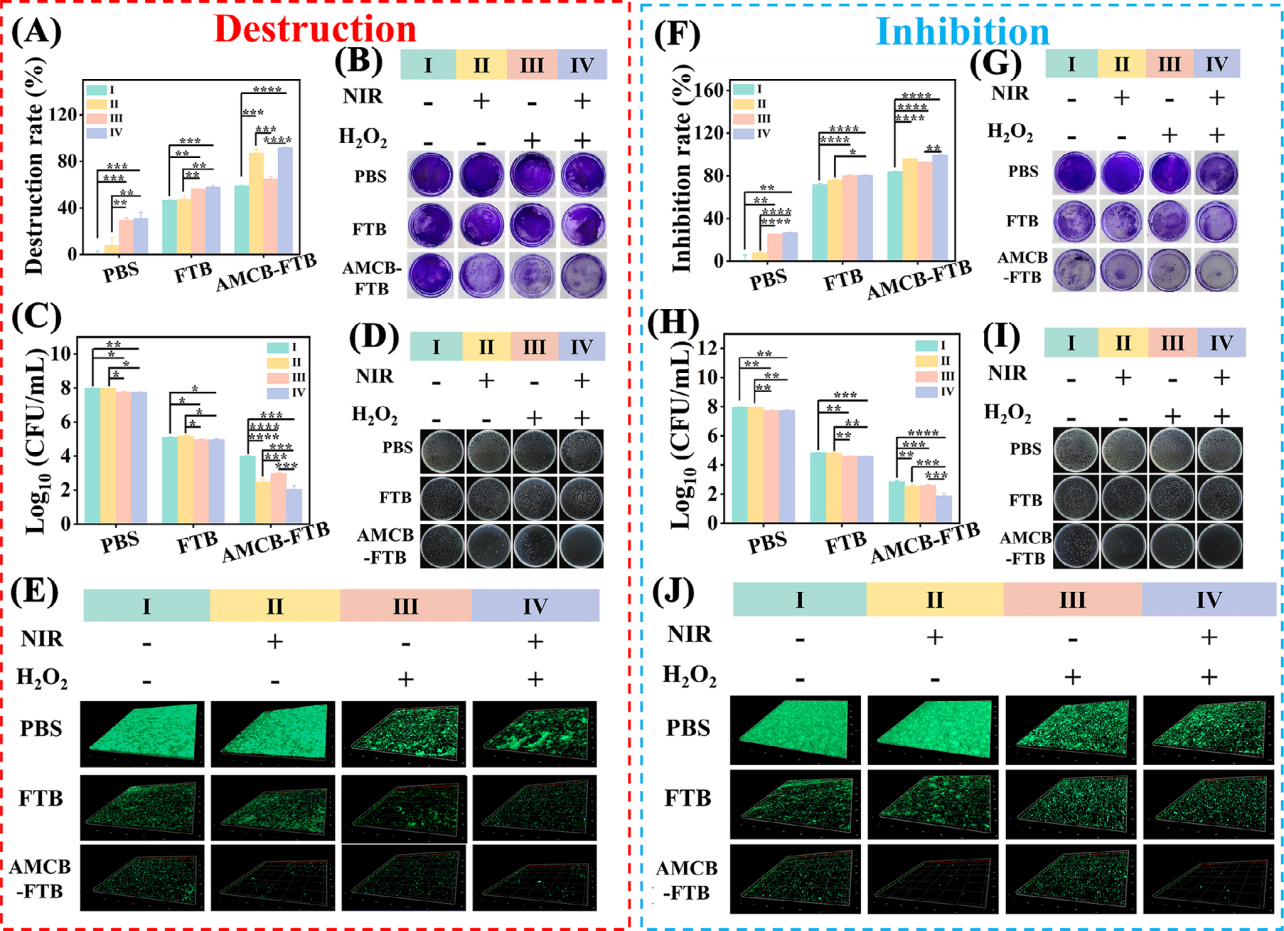
Anti‐biofilm activity in vitro. A) Destruction rate of *P. aeruginosa* mature biofilm in each group. B) Crystalline violet staining images of mature biofilms under different treatment conditions. C) CFU count of *P. aeruginosa* mature biofilms in different groups. D) The agar plate photographs of *P. aeruginosa* mature biofilms in different groups. E) 3D structural images of mature biofilms of *P. aeruginosa* with different treatments. F) Inhibition rate of *P. aeruginosa* biofilm formation in each group. G) Crystalline violet staining images of immature biofilms under different treatment conditions. H) CFU count of *P. aeruginosa* immature biofilms in different groups. I) The agar plate photographs of *P. aeruginosa* immature biofilms in different groups. J) 3D structural images of immature biofilms of *P. aeruginosa* with different treatments. I: Treatments without NIR and H_2_O_2_. II: Treatments with NIR but without H_2_O_2_. III: Treatments without NIR but with H_2_O_2_. IV: Treatments with NIR and H_2_O_2_. Data are presented as mean ± SD (*n* = 3). Statistical significance was tested with two‐way ANOVA, *p < 0.05, **p < 0.01, ***p < 0.001, ****p < 0.0001.

The ability of biofilms to secrete extracellular polymeric substances that encapsulate bacterial cells and enhance antibiotic resistance represents a major challenge in treating bacterial infections. To address this, we evaluated the biofilm eradication efficacy of FTB and AMCB‐FTB. As expected, biofilms treated with AMCB‐FTB + NIR, AMCB‐FTB + H_2_O_2_, or AMCB‐FTB + NIR + H_2_O_2_ were nearly completely removed, with only sporadic residues remaining. In contrast, biofilms treated with PBS or PBS + H_2_O_2_ remained dense and structurally intact (**Figure** **6**B; Figure S23B, Supporting Information). A significant reduction in absorbance at 590 nm was observed in both the FTB and AMCB‐FTB groups compared to the control, indicating effective clearance of mature biofilms (Figure 6A; Figure S23A, Supporting Information). The 3D structure of biofilms was further visualized using laser confocal microscopy (Figure 6E; Figure S23E, Supporting Information). The weakest green fluorescence signal and the thinnest biofilm layers were observed in the AMCB‐FTB + H_2_O_2_ + NIR group, demonstrating the most pronounced disruption of biofilm integrity. Quantification of viable bacteria within the residual biofilm showed a substantial decrease in the AMCB‐FTB‐treated group relative to the control. This reduction was even more pronounced in groups receiving AMCB‐FTB combined with NIR, H_2_O_2_, or both (Figure 6C,D; Figure S23C,D, Supporting Information). Additionally, biofilm formation was inhibited by AMCB‐FTB, AMCB‐FTB + NIR, AMCB‐FTB + H_2_O_2_ and AMCB‐FTB + H_2_O_2_ + NIR treatments (Figure 6F,G; Figure S23F,G, Supporting Information). SYBR Green I staining (Figure 6J; Figure S23J, Supporting Information) and quantitative analysis of live bacteria in the biofilm (Figure 6H,I; Figure S23H,I, Supporting Information) confirmed that AMCB‐FTB + H_2_O_2_ + NIR treatments significantly inhibited biofilm formation, resulting in a looser and weaker biofilm structure. Concurrently, this combination also efficiently killed embedded bacteria within the biofilm. Overall, AMCB‐FTB in combination with H_2_O_2_ and NIR irradiation exhibited strong anti‐biofilm activity, capable of both disrupting preformed mature biofilms and preventing new biofilm formation.

The quorum sensing system and biofilm formation are complex interconnected processes regulated by multiple bacterial physiological activities. We employed RT‐PCR to analyze changes in the expression of genes associated with quorum sensing. As shown in Figure S24A (Supporting Information), genes related to the quorum sensing system and virulence in *P. aeruginosa* were significantly down‐regulated after treatment with AMCB‐FTB + H_2_O_2_ + NIR. A similar down‐regulation trend was observed in *E*. *coli* (Figure S25A, Supporting Information). Furthermore, we quantified the contents of extracellular DNA (eDNA), extracellular polysaccharides (EPS), and protease activity. After treatment with AMCB‐FTB + H_2_O_2_ + NIR, both EPS and eDNA levels were markedly reduced, and protease activity was significantly decreased in both *P. aeruginosa and E. coli* (Figures S24B–D and S25B–D, Supporting Information). The production of pyocyanin by *P. aeruginosa* was also quantified. Similarly, the yield of pyocyanin also decreased significantly after AMCB‐FTB + H_2_O_2_ + NIR treatment (Figure S26A,B, Supporting Information).

### 2.6 RNA Sequencing Analysis

**Figure 7:**
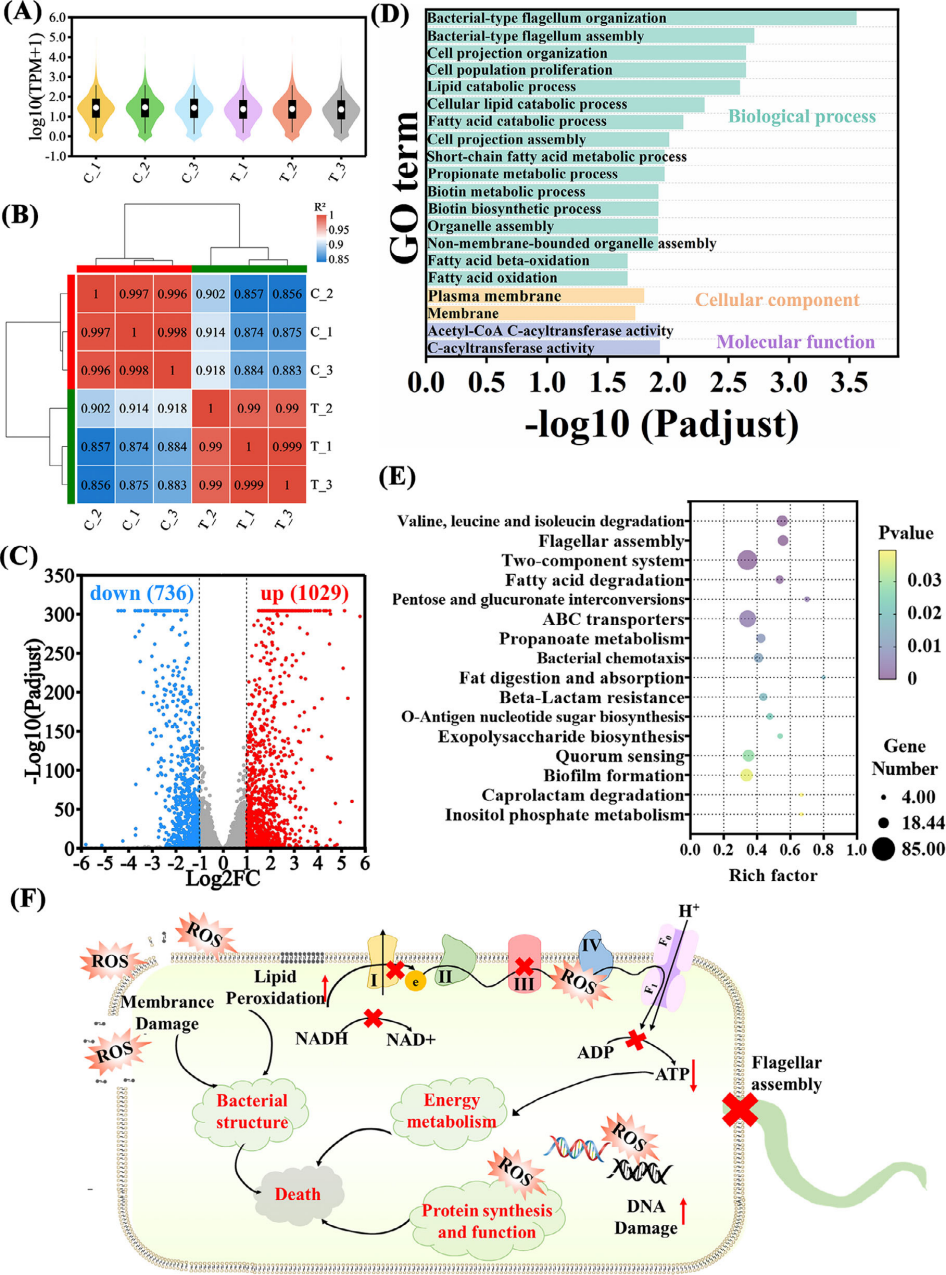
A) Violin plot of overlapping DEGs of *P. aeruginosa* after AMCB‐FTB + NIR + H_2_O_2_ treatment. B) Pearson correlation analysis of DEGs. C) Volcano plot of differentially expressed genes (DEGs) (FDR: false discovery rate, FC: fold change). D) GO enrichment analysis of DEGs. E) Scatterplot of Kyoto Encyclopedia of genes and genomes (KEGG) enrichment analysis. F) Schematic diagram of the antimicrobial mechanism of AMCB‐FTB. C_1‐C_3: Control; T_1‐T_3: AMCB‐FTB + NIR + H_2_O_2_ treatment group.

Given that AMCB‐FTB have potent antibacterial effects against *P. aeruginosa* and *E. coli*, we employed RNA sequencing transcriptomics to elucidate its mechanism of action in *P. aeruginosa*. To evaluate the expression level of individual genes, RNA sequencing by expectation‐maximization (RSEM) values were calculated by comparing fragments per kilobase of transcript per million mapped reads (FPKM) across different samples. The resulting violin plots illustrate the distribution and dispersion of gene expression levels, highlighting the differences between the control and AMCB‐FTB + NIR + H_2_O_2_‐treated groups (**Figure** **7**A). Pearson correlation analysis revealed a reduction in R^2^ values between the AMCB‐FTB + NIR + H_2_O_2_ treatment group and the control group, which might reflect the presence of differentially expressed genes (DEGs) induced by the treatment. Nonetheless, all R^2^ values exceeded 0.85, indicating a strong correlation among the replicate samples and confirming the high repeatability of the samples (Figure 7B). The heatmaps verified the gene expression of different groups. Red indicates up‐regulated genes and blue indicates down‐regulated genes, demonstrating the consistency of gene changes after treatment with AMCB‐FTB + NIR + H_2_O_2_ (Figure S27, Supporting Information). Differential expression analysis identified that, compared with the control group, there were a total of 1765 DEGs in the AMCB‐FTB + NIR + H_2_O_2_ treated group. Among these, 1029 genes were upregulated and 736 genes were downregulated (Figure 7C). To reveal the functional classes of DEGs, we performed Kyoto Encyclopedia of Genes and Genomes (KEGG) and Gene Ontology (GO) enrichment analyses. GO analyses revealed that AMCB‐FTB+ NIR + H_2_O_2_ induced *P. aeruginosa* DEGs are related to multiple aspects such as flagellar organization and assembly, population proliferation, fatty acid, lipid, and biotin metabolism, as well as cellular components and molecular functions (Figure 7D).

KEGG pathway enrichment analysis revealed significant changes in bacterial metabolic and regulatory networks (Figure 7E). Pathways related to amino acid metabolism, such as valine, leucine, and isoleucine degradation, as well as fatty acid and propanoate metabolism, were markedly enriched, suggesting a disruption of fundamental energy supply and biosynthetic processes critical for bacterial survival.^[^ ^43^ ^]^ Moreover, enrichment of pathways including flagellar assembly, bacterial chemotaxis, quorum sensing, and biofilm formation indicates that the hydrogel treatment interferes with bacterial motility and communication, thereby hindering colonization and persistence.^[^ ^44^, ^45^ ^]^ Notably, pathways associated with β‐lactam resistance, ABC transporters, and O‐antigen nucleotide sugar biosynthesis were also affected, implying suppression of bacterial defense mechanisms and cell envelope integrity.^[^ ^46^, ^47^, ^48^ ^]^ Collectively, these findings indicate that the AMCB‐FTB hydrogel not only disrupts core metabolic functions but also attenuates virulence and resistance traits, thereby elucidating the mechanistic basis for its potent antibacterial activity. Then, we focused on the expression of differential genes in these pathways and constructed a protein‐protein interaction (PPI) network, confirming that AMCB‐FTB + NIR + H_2_O_2_ significantly affected *P. aeruginosa* in terms of energy metabolism, amino acid metabolism, structural integrity of the bacterial cell, and biofilm formation and pathogenicity (Figure S28, Supporting Information). These data suggested that AMCB‐FTB + H_2_O_2_ + NIR treatment induced the expression of DEGs, which are involved in several important biological reactions that affect the basic bacterial life activities, weaken bacterial biofilm formation and virulence (Figure 7F). To further clarify the specific contribution of each component within the AMCB‐FTB hydrogel system, we conducted RT‐PCR validation across multiple experimental groups, including control (C_1‐C_4), FTB (F_1‐F_4), AMCB (A_1‐A_4), and AMCB‐FTB (AF_1‐AF_4). As shown in Figure S29 (Supporting Information), in the FTB hydrogel group and the AMCB nanoparticle group, the expression levels of key target genes (such as *ftsI*, *phoP*, *phoQ*, *pmrA*, *motD*, *flhA*, and *mexY*) differed from those in the control group, but the differences were relatively minor. In contrast, significant changes were observed in the AMCB‐FTB hydrogel under NIR irradiation. These results suggest that the transcriptional changes are attributable to the synergistic action of the hydrogel active components and AMCB nanoparticles, with enhanced effects under NIR irradiation.

### 2.7 AMCB Enzyme‐Mimicking Activities under Neutral Conditions

**Figure 8:**
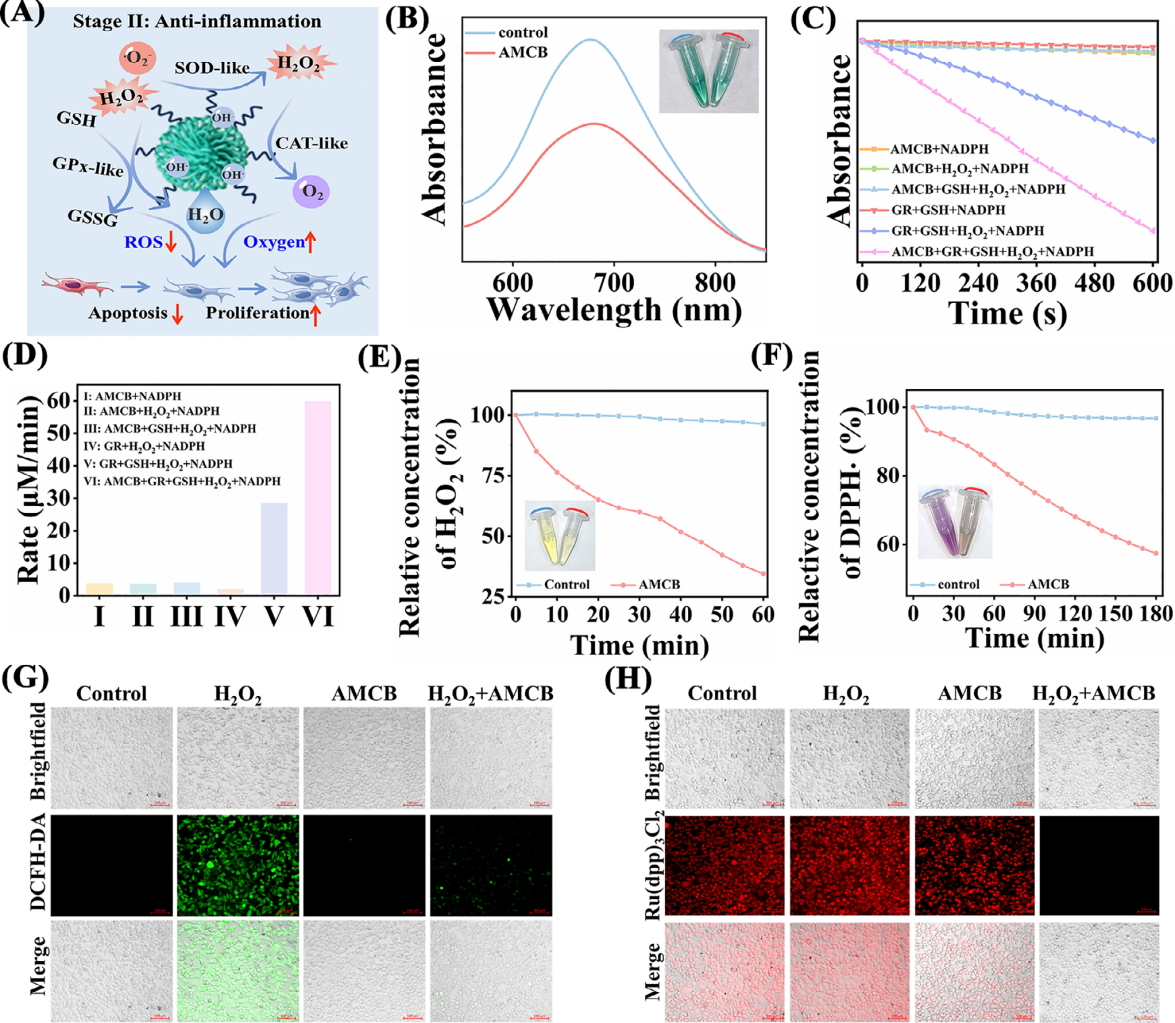
A) Schematic representation of ROS scavenging and attenuation of cellular oxidative stress by AMCB. B) SOD‐like enzyme activity of AMCB. C) GPx‐like activity of AMCB assessed by NADPH depletion. D) Initial rate of GPx‐like activity under different conditions. E) H_2_O_2_ scavenging by AMCB. F) Scavenging of DPPH∙ by AMCB. G) Fluorescence images of DCFH‐DA stained NIH/3T3 cells after different treatments. H) O_2_ levels in NIH/3T3 cells after [Ru(dpp)_3_]Cl_2_ staining after different treatments.

Hypoxia and ROS overexpression in wounds after antimicrobial treatment are the major factors that impact cell survival, migration, and vascularization, resulting in delayed wound healing.^[^ ^49^ ^]^ Although AMCB exhibits pH‐dependent ROS generation and strong antimicrobial effect under acidic conditions (typical infection environment), we explored its dual role in scavenging ROS and mitigating oxidative stress at neutral pH (as in inflammatory sites). To explore the ROS scavenging and anti‐oxidative stress activity of AMCB under neutral conditions, we first assessed its SOD‐like activity using a nitro blue tetrazolium chloride (NBT) colorimetric assay. As demonstrated in **Figure** **8**B, AMCB effectively scavenged ≈37.81% of •O_2_^−^ within 5 min, demonstrating significant SOD‐like activity. The GPx‐like catalytic activity of AMCB was further evaluated by monitoring NADPH consumption. As shown in Figure 8C, the most significant decrease in NADPH concentration occurred in the presence of AMCB combined with glutathione reductase (GR), GSH, and H_2_O_2_, confirming that AMCB has a strong GPx‐like catalytic activity under these conditions. In contrast, AMCB alone did not reduce NADPH levels (Figure 8D), indicating that the antioxidant effect is mediated through GPx‐like enzymatic activity rather than direct NADPH oxidation. AMCB also exhibited CAT‐like activity, effectively removing 65.49% of H_2_O_2_ within 60 min, thereby preventing excessive intracellular H_2_O_2_ accumulation (Figure 8E). Additionally, AMCB exhibited time‐dependent scavenging capability against 1,1‐diphenyl‐2‐picrylhydrazyl (DPPH) radicals, reducing DPPH• by 42.63% over 180 min (Figure 8F). We further determined the SOD‐like (Figure S30, Supporting Information) and CAT‐like (Figure S31, Supporting Information) activities of AMCB under various environmental conditions and time intervals. The results demonstrated that the catalytic efficiency remained robust even under high redox stress and in the presence of high protein concentrations, confirming the strong enzymatic stability of AMCB. Collectively, these findings highlight the potent free radical scavenging capacity of AMCB in vitro.

Furthermore, we established an H_2_O_2_‐induced oxidative stress model to evaluate the antioxidant effects of AMCB at the cellular level. First, MTT assays confirmed that AMCB did not exhibit significant cytotoxicity or inhibit cell proliferation (Figure S32, Supporting Information). The efficiency of AMCB in scavenging intracellular ROS was evaluated using the fluorescent probe DCFH‐DA. As shown in Figure 8G, strong green fluorescence was observed in the H_2_O_2_‐treated group, indicating high levels of intracellular ROS. In contrast, AMCB treatment significantly reduced ROS‐related fluorescence, demonstrating its potent ROS‐scavenging capacity and antioxidative properties. Changes in intracellular O_2_ levels were monitored using the oxygen‐sensitive fluorescent probe [Ru(dpp)_3_]Cl_2_. Treatment with AMCB led to a noticeable increase in intracellular O_2_ concentration, confirming its oxygen‐generating ability (Figure 8H). These findings demonstrate that AMCB effectively scavenges ROS and releases oxygen, thereby mitigating oxidative stress and alleviating the hypoxic microenvironment. This dual functionality helps reduce cellular damage, promotes cell proliferation, and may facilitate enhanced wound healing.

**Figure 9:**
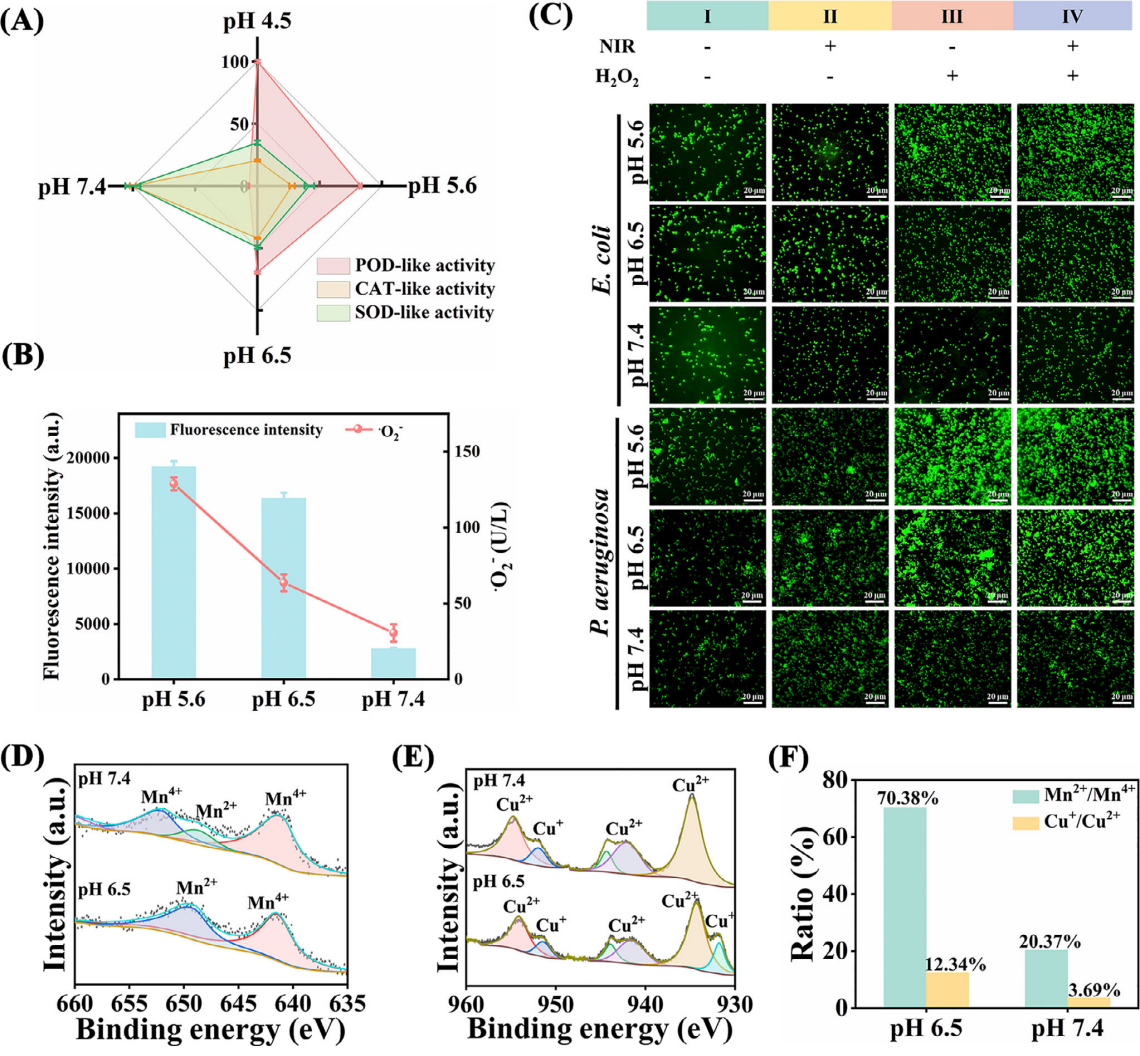
pH‐dependent valence states of AMCB. A) Enzyme activity at different pH conditions (4.5, 5.6, 6.5, and 7.4). B) The generation of O_2_ and •O_2_^−^ of AMCB under different pH conditions. Data are presented as mean ± SD (*n* = 3, independent measurements). C) Fluorescence images of *E. coli* and *P. aeruginosa* stained with DCFH‐DA after different treatments at different pH (5.6, 6.5 and 7.4). I: Treatments without NIR and H_2_O_2_. II: Treatments with NIR but without H_2_O_2_. III: Treatments without NIR but with H_2_O_2_. IV: Treatments with NIR and H_2_O_2_. D) High‐resolution XPS spectra of Mn 2p at pH 6.5 and pH 7.4. E) High‐resolution XPS spectra of Cu 2p at pH 6.5 and pH 7.4. F) Bar plots of Mn^2+^/Mn^4+^, Cu^+^/Cu^2+^ ratios at pH 6.5 and pH 7.4.

To investigate the influence of pH on enzyme‐like activity, we first evaluated the enzyme‐like activity of AMCB under different pH conditions (4.5, 5.6, 6.5, and 7.4). As shown in **Figure** **9**A, AMCB exhibited significantly higher POD‐like activity compared to its SOD‐like and CAT‐like activities under acidic conditions, favoring the production of ROS. In contrast, AMCB showed markedly enhanced SOD‐like and CAT‐like activities over its POD‐like activity under neutral conditions, thereby promoting antioxidant effects. This conclusion was further corroborated through a quantitative evaluation of the conversion efficiency of SOD‐like and CAT‐like activities by measuring the production of O_2_ and •O_2_^−^ under varying pH conditions (Figure 9B). The results demonstrated that under acidic pH conditions, the production of •O_2_^−^ was elevated while O_2_ generation remained low. In contrast, when the pH increased to neutral, O_2_ production rose significantly accompanied by a reduction in •O_2_^−^ levels. These findings indicate that AMCB can dynamically modulate its SOD‐like and CAT‐like activities to effectively eliminate ROS under neutral conditions, thereby facilitating tissue repair. Furthermore, we dynamically monitored the changes in ROS levels in infected wounds across different treatment groups from the “antibacterial phase (acidic environment)” to the “healing phase (neutral environment)” using a time‐resolved fluorescent probe (DCFH‐DA). The results demonstrated that the AMCB + NIR + H_2_O_2_ treatment group effectively generated ROS under acidic conditions, while the ROS levels gradually decreased as the environment shifted to neutral during the healing process (Figure 9C). To elucidate the mechanism by which pH affects enzyme activity, we determined the valence changes in the molar ratios of Cu^2+^/Cu^+^ and Mn^2+^/Mn^4+^ under different pH conditions using high‐resolution XPS. The results revealed a higher proportion of Cu^+^ and Mn^2+^ at pH 6.5, while Cu^2+^ and Mn^4+^ dominated at pH 7.4 (Figure 9D–F). At pH 6.5, the enrichment of Cu^+^ promotes the conversion of hydrogen peroxide into •OH via POD/Fenton‐like pathways (Cu^+^ + H_2_O_2_ → Cu^2+^ + •OH), thereby enhancing ROS generation and contributing to antimicrobial activity. In contrast, at pH 7.4, the enrichment of Mn^4+^ (accompanied by Cu^2+^) activates CAT/SOD‐like pathways (2H_2_O_2_ → 2H_2_O + O_2_, •O_2_^−^ disproportionation), which facilitates ROS scavenging and supports the healing process. In summary, AMCB can dynamically meet the requirements of infection control and tissue repair through a “switch” between pro‐oxidant and antioxidant functions, which is an adaptive regulation process driven by pH and redox status.

### 2.8 Phenotypic Regulation and Enhanced Cell Migration of Macrophages

**Figure 10:**
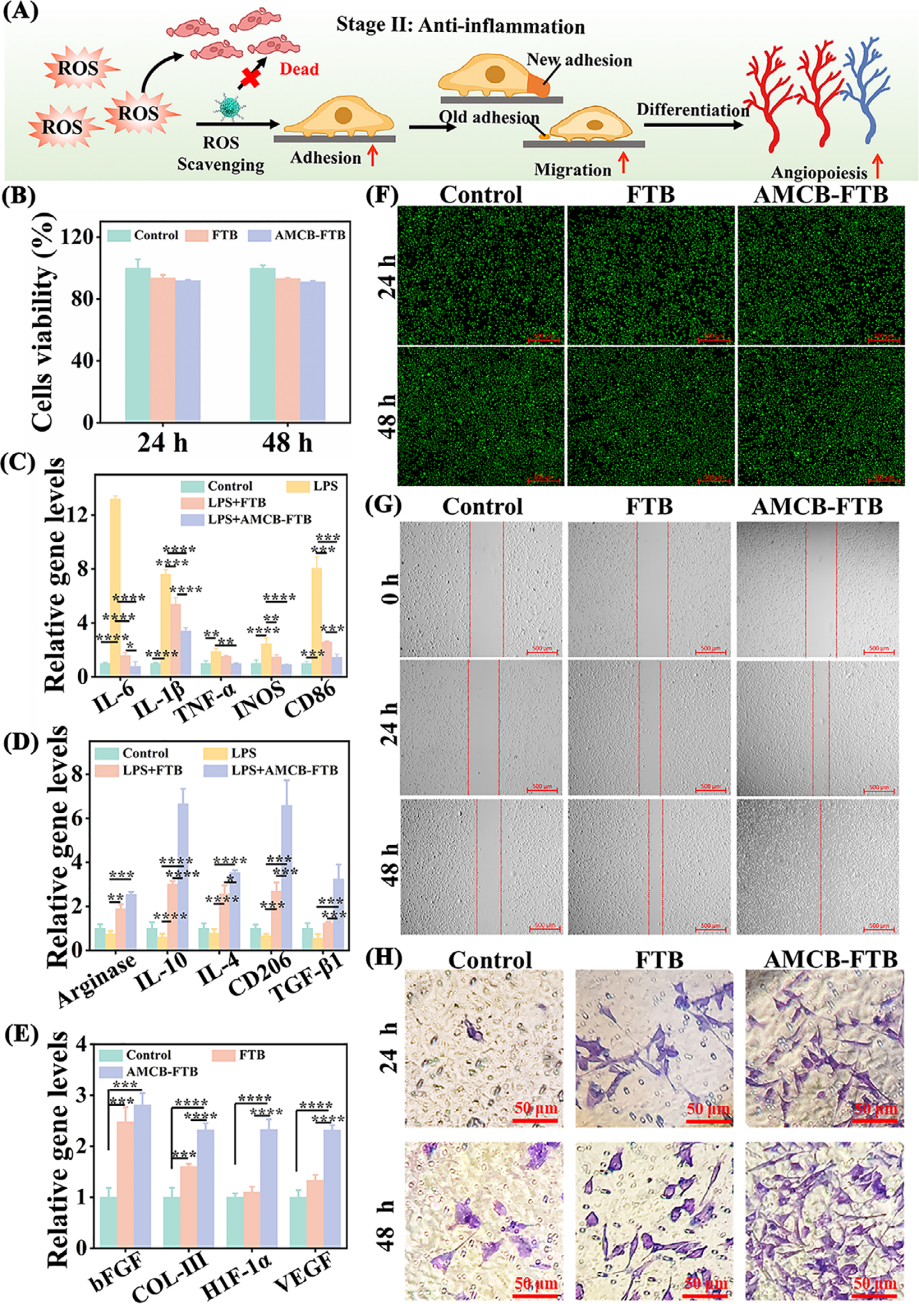
A) Schematic diagram of the mechanism by which ROS scavenging improves endothelial cell adhesion, migration, and angiogenesis. B) The cell viability after co‐culture with FTB and AMCB‐FTB for 24 and 48 h was determined by MTT assay. Data are presented as mean ± SD (*n* = 6, independent measurements). C) Relative gene expression of interleukin‐6 (IL‐6), interleukin‐1β (IL‐1β), tumor necrosis factor‐α (TNF‐α), inducible nitric oxide synthase (iNOS) and cluster of differentiation 86 (CD86) of macrophages. D) Relative gene expression of arginase, interleukin‐10 (IL‐10), interleukin‐4 (IL‐4), mannose receptor (CD206) and transforming growth factor‐β1 (TGF‐β1) of macrophages. E) The expression levels of angiogenesis and epithelialization‐related genes in NIH/3T3 cells cultured on different materials were detected by qRT‐PCR. Data are presented as mean ± SD (*n* = 3). Statistical significance was tested with two‐way ANOVA, *p < 0.05, **p < 0.01, ***p < 0.001, ****p < 0.0001. F) Live/dead staining of NIH/3T3 after incubation for 24 and 48 h. G) Cell scratch images of cell migration measured at 24 and 48 h of culture (scale bars: 500 µm). H) NIH/3T3 migration image stained with crystal violet under light microscopy (scale bars: 50 µm).

The basic requirement for hydrogels in clinical biomaterials is good biocompatibility. As shown in **Figures** **10**B and S33 (Supporting Information), no significant cytotoxicity was observed at 24 and 48 h of treatment. The viability of both NIH/3T3 and RAW 264.7 cells exceeded 90% across all hydrogel groups, indicating that AMCB‐FTB possesses good biocompatibility. Live/dead staining results further supported its favorable cytocompatibility (Figure 10F). Cell migration is crucial for wound recovery. We investigated the effect of AMCB‐FTB on cell migration using cell scratch assay and transwell migration assays. As presented in Figure 10G,H, AMCB‐FTB exhibited the strongest promotive effect on cell migration. To evaluate the anti‐inflammatory activity of AMCB‐FTB, we investigated the effect of AMCB‐FTB on lipopolysaccharide (LPS)‐induced expression of M1/M2 macrophage polarization markers in RAW264.7 cells. After LPS stimulation, M1‐related genes (interleukin‐6 (IL‐6), interleukin‐1β (IL‐1β), tumor necrosis factor‐α (TNF‐α), inducible nitric oxide synthase (iNOS), and cluster of differentiation 86 (CD86)) were dramatically upregulated (Figure 10C). Their expression was slightly inhibited in the FTB group, while AMCB‐FTB treatment significantly suppressed all five M1 markers under inflammatory conditions. Conversely, the expression of M2‐associated genes (arginase, interleukin‐10 (IL‐10), interleukin‐1α (IL‐1α), mannose receptor (CD206), and transforming growth factor‐β1 (TGF‐β1)) was notably increased in the AMCB‐FTB group compared to both the LPS and FTB groups (Figure 10D). To further validate the effect of AMCB‐FTB treatment on promoting M2 macrophage polarization, flow cytometry analysis was performed according to the method reported in the literature.^[^ ^50^, ^51^ ^]^ The expression of the M2 macrophage marker CD206 was used as a representative indicator. As shown in Figure S34 (Supporting Information), compared with the control group, the expression of the M2 macrophage marker CD206 was significantly upregulated in the AMCB‐FTB group. The proportion of CD206‐positive cells increased from 0.019% to 12.9% after AMCB‐FTB treatment. These results demonstrate that AMCB‐FTB possesses significant anti‐inflammatory properties and effectively promotes macrophage polarization toward the M2 phenotype, thereby contributing to the regulation of inflammation and facilitating subsequent tissue repair.

Type collagen III (COL‐III) is the main collagen in the skin and fascia tissues, where it provides essential structural support and facilitates tissue regeneration during wound healing.^[^ ^52^ ^]^ Vascular endothelial growth factor (VEGF) and basic fibroblast growth factor (bFGF) are key regulators of angiogenesis, promoting the formation of new blood vessels essential for tissue repair and regeneration.^[^ ^53^ ^]^ Hypoxia‐inducible factor‐1α (HIF‐1α) serves as a critical mediator of cellular differentiation and migration, particularly under hypoxic conditions, and is integral to tissue regeneration processes.^[^ ^54^ ^]^

We analyzed the expression levels of these vascularization‐ and collagen‐related genes (COL‐III, VEGF, bFGF, and HIF‐1α) using qRT‐PCR. As demonstrated in Figure 10E, after 24 h of co‐culture with NIH/3T3 cells, the AMCB‐FTB group exhibited significantly elevated expression of all four genes compared to both the control and FTB groups. These results suggest that AMCB‐FTB plays a substantial role in enhancing tissue repair and promoting extracellular protein deposition (Figure 10A).

### 2.9 In Vivo Therapeutic Efficacy of AMCB‐FTB in Mice with *P. aeruginosa*‐Infected Wound

**Figure 11:**
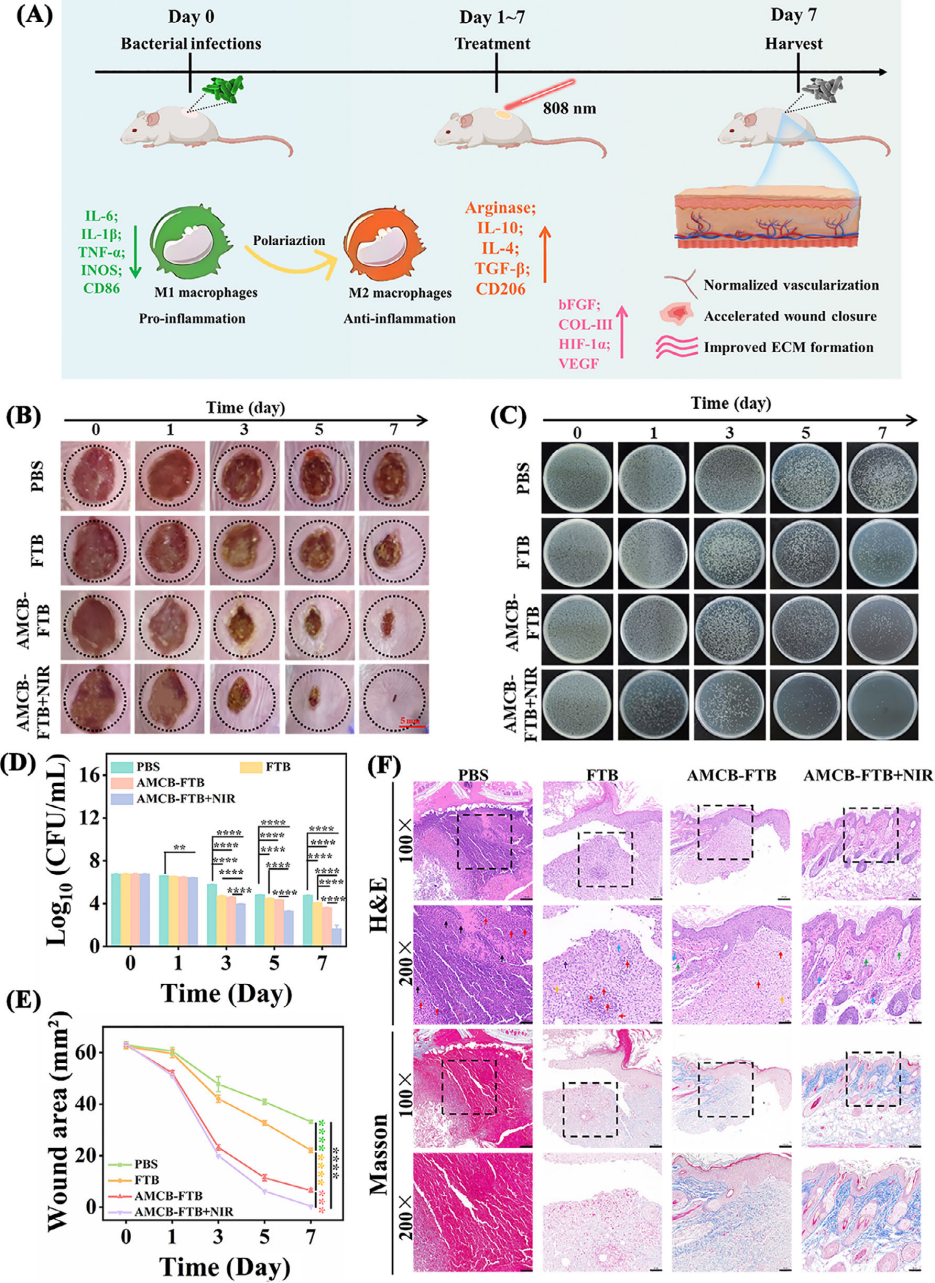
In vivo antibacterial activity of AMCB‐FTB. A) Construction and treatment diagram of the infection wound model. B) Photographs of *P. aeruginosa* infected wounds after various treatments (scale bar = 5 mm). C) Colonies growth of different groups after different treatment. D) The number of bacteria surviving in the skin tissue was counted after treatment. Data are presented as mean ± SD (*n* = 6). Statistical significance was tested with two‐way ANOVA, **p < 0.01, ****p < 0.0001. E) Changes in wound area of different groups on different days. Data are presented as mean ± SD (*n* = 6). Statistical significance was tested with one‐way ANOVA, ****p < 0.0001. F) H&E and Masson staining of wound tissue.

Based on the adaptive ROS regulation ability of the infection and inflammatory microenvironment programmed by AMCB, we further explored the therapeutic effect of FTB or AMCB‐FTB hydrogel as wound dressings for treating *P. aeruginosa* infected wounds in vivo. The animal experiments followed the principles established by the Animal Ethics Committee of Tianjin University of Science & Technology (20240410). BALB/c mice were randomly divided into four groups: PBS, FTB, AMCB‐FTB, and AMCB‐FTB + NIR group (**Figure** **11**). We first evaluated the in vivo photothermal performance of AMCB‐FTB using an infrared camera. In Figure S35E (Supporting Information), after 15 s of NIR irradiation, the local wound temperature in the AMCB‐FTB group rose to 43.2 °C, confirming its potential as an effective photothermal wound dressing. To monitor wound recovery, digital images were taken at various time periods. In Figure 11B, all hydrogel‐treated groups showed significantly accelerated wound closure compared to the PBS group. On day 7, the wound healing rate in the PBS group was only 47.29%, whereas the FTB group reached 64.52%, demonstrating the intrinsic wound‐healing promotion ability of FTB. In contrast, the healing rates of the AMCB‐FTB group and AMCB‐FTB + NIR group were 89.68% and 99.38%, respectively, suggesting that AMCB‐FTB could effectively promote the wound healing process through photothermal therapy (Figure 11E). Notably, the combination of AMCB‐FTB with NIR irradiation resulted in the most pronounced wound healing effect on days 3, 5, and 7 (Figure S35A–D, Supporting Information). Additionally, the in vivo antibacterial effects of FTB and AMCB‐FTB were also evaluated. The results revealed that the AMCB‐FTB group effectively suppressed bacterial growth, with the strongest antibacterial activity observed in the AMCB‐FTB + NIR group, achieving an inhibition rate of 99.95% (Figure 11C,D). Collectively, these findings demonstrate that the AMCB‐FTB hydrogel synergistically combines photothermal sterilization with enhanced wound‐healing functions.

Histological analysis was performed to evaluate wound tissue repair in each treatment group. In Figure 11F, skin tissues in the PBS group exhibited severe structural abnormalities, including extensive necrotic cells (black arrow), dense inflammatory cell infiltration (red arrow), unhealed ulcerated wounds, and complete absence of dermal architecture. The FTB group also showed markedly abnormal skin structure, characterized by lack of stratum spinosum thickening, absence of dermal collagen fibers (yellow arrow), and substantial inflammatory cell infiltration (red arrow). In the AMCB‐FTB group, skin structure displayed only mild abnormalities. The epidermis remained intact within the field of view without spinous layer thickening. Although dermal collagen fibers were reduced, some skin appendages were visible (yellow arrow), along with minimal inflammatory cell infiltration (red arrow). By contrast, the AMCB‐FTB + NIR group presented nearly normal skin morphology, featuring an intact epidermal layer without spinous layer thickening. Dermal collagen fibers were abundant, densely packed, and well‐organized. Skin appendages appeared normal, and no inflammatory cell infiltration was observed. Together, these results demonstrate that AMCB‐FTB combined with NIR irradiation effectively attenuates inflammation, promotes collagen deposition, and accelerates the healing of infected wounds.

**Figure 12:**
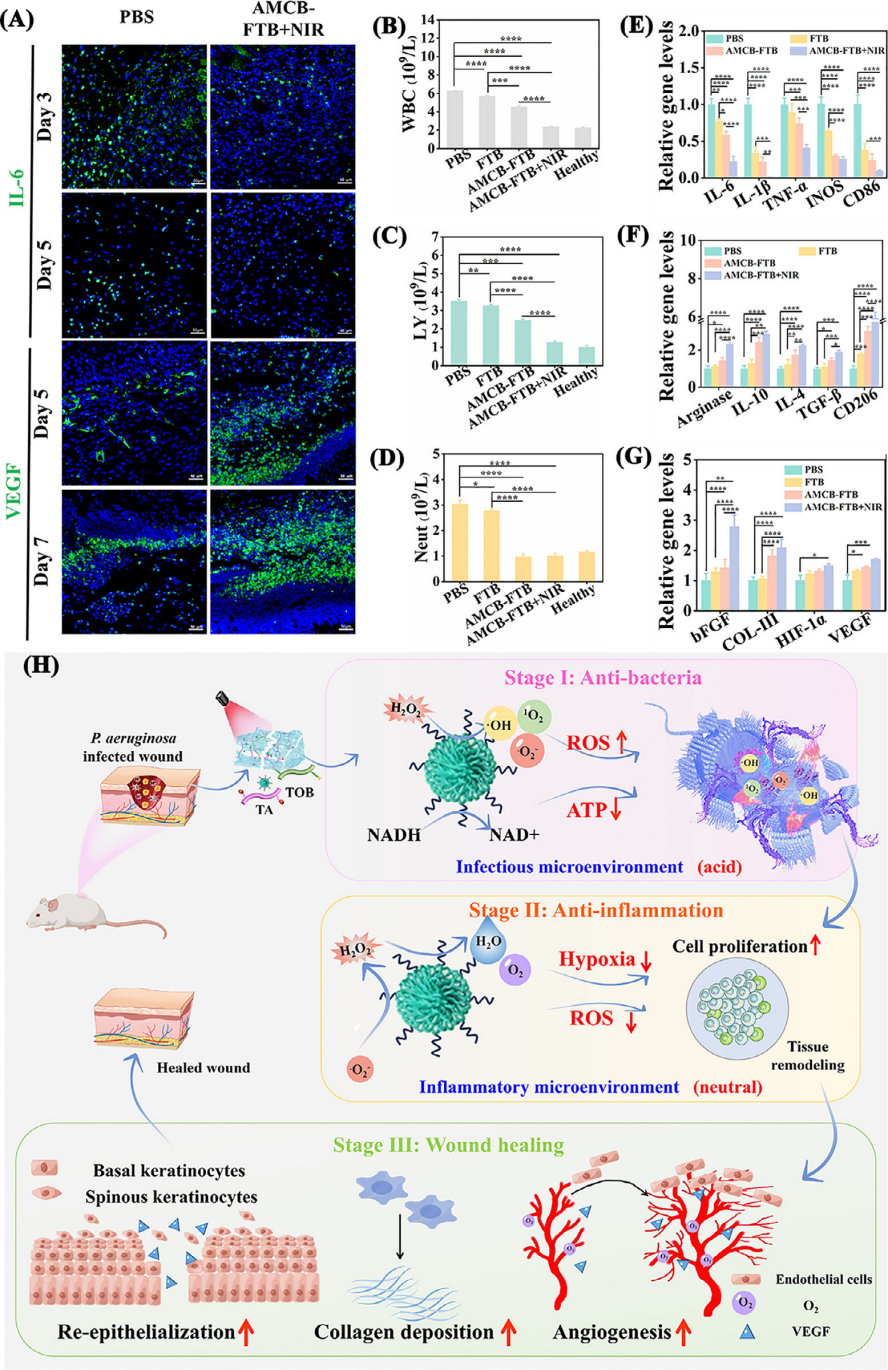
A) Immunohistochemical staining of IL‐6 and VEGF in the treated skin tissues. Changes in white blood cell (B) lymphocytes (C) and neutrophils (D) in each group after 7 days of different treatment. Data are presented as mean ± SD (*n* = 3). Statistical significance was tested with one‐way ANOVA, *p < 0.05, **p < 0.01, ***p < 0.001, ****p < 0.0001. E) qRT‐PCR results of pro‐inflammatory related genes. F) qRT‐PCR result of anti‐inflammatory‐related genes. G) qRT‐PCR result of angiogenesis‐related genes. Data are presented as mean ± SD (*n* = 3). Statistical significance was tested with two‐way ANOVA, *p < 0.05, **p < 0.01, ***p < 0.001, ****p < 0.0001. H) A schematic illustrates the controlled release mechanism of AMCB‐FTB, highlighting its roles in antimicrobial activity, oxygen generation, oxidative stress alleviation, and tissue regeneration promotion.

Immunofluorescence analysis was performed on *P. aeruginosa*‐infected wounds to evaluate the expression of IL‐6 and VEGF, which reflect inflammatory levels and angiogenesis, respectively. As illustrated in **Figure** **12**A, treatment with AMCB‐FTB + NIR resulted in decreased IL‐6 expression and increased VEGF expression compared to the PBS group, indicating a reduction in inflammation and enhancement of angiogenic activity. Furthermore, after 7 days of AMCB‐FTB + NIR treatment, hematological indices associated with the inflammatory response, such as white blood cells (WBC, Figure 12B), lymphocytes (LY, Figure 12C), and neutrophils (Neut, Figure 12D), returned to the same levels as those in healthy mice. Total RNA was then extracted from the wound tissue, and qRT‐PCR analysis was performed to quantify the expression of pro‐inflammatory and anti‐inflammatory factors as well as genes associated with wound healing. The results showed that after 7 days of AMCB‐FTB + NIR treatment, the expression of pro‐inflammatory factors associated with the M1 phenotype were significantly down‐regulated (Figure 12E), while the expression of anti‐inflammatory factors associated with the M2 phenotype (Figure 12F) and genes promoting of wound recovery (Figure 12G) were markedly upregulated. Quantitative analysis of serum IL‐1β and TNF‐α revealed that the AMCB‐FTB + NIR treatment group exhibited the lowest levels (Figure S36A,B, Supporting Information). In conclusion, our findings demonstrate that AMCB‐FTB, integrating drug controlled release with effective antibacterial, anti‐inflammatory and angiogenic properties, and can be used as an efficient and safe wound dressing to treat wounds infected with *P. aeruginosa* through microenvironmental adaptive therapy (Figure 12H).

### 2.10 Biocompatibility of AMCB‐FTB

When developing therapeutic materials, ensuring their biocompatibility is essential. We performed routine blood tests and histological evaluations on mouse samples after 7 days of antimicrobial treatment with AMCB‐FTB + NIR. The histological structures of major organs such as the heart, liver, spleen, lung, and kidney were observed by H&E staining (Figure S37A, Supporting Information). The results showed that compared with the PBS group, no significant tissue abnormalities or pathological changes were observed in the major organs of the mice in the AMCB‐FTB + NIR group. Additionally, we used mouse erythrocytes to determine the hemolytic activity of the hydrogel. Ultrapure water was used as a positive control, and PBS was used as a negative control. As shown in Figure S37B (Supporting Information), clear hemolysis was observed in the positive control, while the supernatants from both the FTB and AMCB‐FTB groups appeared light yellow, similar to the PBS group. The hemolysis rates in these groups were well below the permissible threshold of 5%. We further evaluated the coagulation performance of the hydrogels using a dynamic whole blood coagulation assay. The blood coagulation index (BCI) values for the FTB and AMCB‐FTB groups were 6.91% and 7.04%, respectively, indicating favorable coagulation effects (Figure S37C, Supporting Information). Additionally, mouse body weight was monitored throughout the treatment period and showed no significant differences among groups (Figure S37D, Supporting Information), suggesting that AMCB‐FTB does not induce obvious systemic toxicity.

By routine blood analysis, comparative analysis of blood indices between the experimental and normal groups of mice showed that parameters indicative of oxygen‐carrying capacity, such as red blood cell count (RBC), hemoglobin (HGB), hematocrit (HCT), mean corpuscular volume (MCV), mean corpuscular hemoglobin concentration, (MCHC), mean corpuscular hemoglobin (MCH), and red blood cell distribution width (RDW), did not deviate significantly from the level of normal mice deviation. Similarly, mean platelet volume (MPV), platelet distribution width (PDW), and thrombocytocrit (PCT) of whole blood showed consistency in all study groups (Figure S37E, Supporting Information), confirming that coagulation was not impaired. Serum alanine aminotransferase (ALT, Figure S38A, Supporting Information), aspartate aminotransferase (AST, Figure S38B, Supporting Information), blood urea nitrogen (BUN, Figure S38C, Supporting Information), and creatinine (CR, Figure S38D, Supporting Information) levels in mice also showed no significant changes after different treatments. Together, the H&E staining and comprehensive hematological analyses demonstrate that the multifunctional hydrogel, along with its individual components and the accompanying mild thermal therapy, exerted negligible effects in vivo, further supporting its favorable biocompatibility.

## 3 Conclusion

In this study, we present a microenvironment‐responsive artificial multicatalytic hydrogel (AMCB‐FTB) with multiple enzyme‐like activities for targeted treatment of *P. aeruginosa*‐infected wounds. The system works through three synergistic mechanisms: 1) The low‐temperature photothermal effect triggered by NIR not only released antibacterial components, but also promoted catalytic therapy based on nanozymes to generate ROS, which disrupted the redox balance and energy metabolism of the bacteria and thus lead to bacterial death. 2) After bacterial clearance, the self‐switching enzymatic activity of AMCB‐FTB eliminates excessive ROS, significantly reduces oxidative stress, promotes cell proliferation and angiogenesis, and ultimately promotes wound tissue regeneration, achieving programmed treatment of bacterial infections and inflammatory wounds. 3) The transcriptome analysis showed that AMCB‐FTB + NIR + H_2_O_2_ treatment could severely inhibit bacterial survival, motility, biofilm formation and virulence by interfering with bacterial energy metabolism, protein synthesis and lipid pathways. Responsive drug release, nanozymatic catalytic therapy, ROS modulation and low‐temperature photothermal therapy create a powerful platform for addressing bacterial infections. The multi‐enzyme mimetic hydrogel strategy not only overcomes the limitations of conventional antimicrobial therapies, but also provides a reference for the design of smart biomaterials for complex wound microenvironments.

## Conflict of Interest

The authors declare no conflict of interest.

## Supporting information



Supporting Information

## Data Availability

The data that support the findings of this study are available from the corresponding author upon reasonable request.
