# Supplementary material for: Trimetallic Nanozyme‐Embedded Smart Hydrogel Enables NIR‐Controlled Bacterial Killing and Oxidative Stress Alleviation
Source: Adv Sci (Weinh). 2025 Oct 3;12(48):e12875. doi: 10.1002/advs.202512875 (PMC12752578; doi:10.1002/advs.202512875)
Supplement: Supplementary file 1 — Supporting Information [file ADVS-12-e12875-s001.docx]

**Trimetallic Nanozyme-Embedded Smart Hydrogel Enables NIR-Controlled Bacterial Killing and Oxidative Stress Alleviation**

Zehui Xiao^^[[1]](#footnote-1)^^, Jiangli Cao^1^, Jifeng Liu^1^, Zhiyong Song^2^, Ting Du^1,^* and Xinjun Du^1,^*

**1. Experimental Section**

*Materials**:* Chloroauric acid (HAuCl_4_) and trypsin were obtained from Sigma-Aldrich Co. (St. Louis, MO, USA). Manganese chloride (MnCl_2_) and copper chloride (CuCl_2_) were purchased from Sinopharm Chemical Reagent Co., Ltd. (Shanghai, China). Tobramycin (TOB), 3-formylphenylboronic acid (3-FPBA), glutathione (GSH), 5, 5'-dithiobis (2-nitrobenzoic acid) (DTNB) and tannic acid (TA) were purchased from Macklin Industrial Inc. (Shanghai, China). 2,7-dichlorodihydrofluorescein diacetate (DCFH-DA), 1,3-diphenylisobenzofuran (DPBF) and [Ru(dpp)_3_]Cl_2_ were purchased from Med Chem Express (Tianjin, China). The ATP assay kit, malondialdehyde (MDA) assay kit, glutathione/oxidized glutathione assay kit was obtained from Nanjing jiancheng Bioengineering institute (Jiangsu, China). All chemicals were used as received without any further purification.

*Synthesis of Sea Urchin-like Nanoparticles (AMC, AMCB):* Briefly, 110 μL of ascorbic acid (AA, 0.5 M) was mixed with 10 mL of Tris-HCl (10 mM), followed by the addition of 100 μL of chloroauric acid (HAuCl_4_, 0.1 M). After 20 s, the sea urchin-like gold nanoparticle was prepared and washed once with deionized water. Subsequently, 3.0 mL of manganese chloride (3.2 mg/mL) was added and ultrasonically treated for 1 min at room temperature. Then, 3.0 mL of copper chloride (1.6 mg/mL) was added and sonication was continued. Finally, the resulting nanoparticles (named AMC) were collected by centrifugal washing.

First, COOH-PEG-SH was added to the AMC solution and stirred for 8 h to form Au-S bonds between COOH-PEG-SH and AMC. Next, the activated COOH-PEG-S-AMC was mixed with polymyxin B (PMB) and gently shaken at room temperature for 8 h. Then the solution was transferred to a dialysis bag (MWCO: 35 kDa) and washed several times with PBS to remove free PMB. The solution was then centrifuged at 10,000 rpm for 6 min. The final purified product (named AMCB) was stored at 4°C for further use.

*Characterization of Sea Urchin-like Nanoparticles (Au NPs, AMC, AMCB)*: The spectral properties of Au NPs, AMC, and AMCB were characterized using UV-vis absorption and Fourier transform infrared spectroscopy (FTIR). The particle size distribution and surface zeta potential of Au NPs, AMC, and AMCB were measured by dynamic light scattering (DLS) using a Malvern Zetasizer. The morphologies and structures of Au NPs, AMC, and AMCB were examined via transmission electron microscopy (TEM). Additionally, the elemental valence and chemical composition of Au NPs, AMC, and AMCB were analyzed using X-ray powder diffraction (XRD) and X-ray photoelectron spectroscopy (XPS).

*Validation of the Multiple Enzyme-Like Activities of AMCB*: The AMCB prepared in this work has four enzymatic activities: POD-like, GSH-Px-like, CAT-like, SOD-like. First, the POD-like enzyme activity of AMCB were evaluated using TMB as probes. Six concentrations (0, 1, 2, 4, 8, and 16 μg/mL) of AMCB were established to determine the POD-like properties. After centrifuging to remove AMCB, the absorbance of the supernatant at 652 nm was determined by ultraviolet-visible scanning spectrophotometer.

Using DTNB as a probe, the GSH depletion capacity was examined by UV-vis spectrophotometer to verify the GSH peroxidase activity of AMCB. 5,5'-dithiobis-(2-nirtobenzoic acid) (DTNB, 2.51 mg) was dissolved in 1.0 mL PBS (0.1 M). Then, AMCB solution with different concentrations (0, 1, 2, 4, 8, and 16 µg/mL ) was reacted with 100 µM GSH at room temperature for 10 min, then 50 µL DTNB solution was added and co-incubated for 10 min. After centrifuging to remove AMCB, the absorbance of the supernatant at 412 nm were measured by UV-vis scanning spectrophotometer.

In a CAT-like assay, H_2_O_2_ (1.0 × 10^-3^ M) was mixed with 4 μg/mL of AMCB in PBS (pH 7.4) to a final volume of 2.0 mL. Aliquots of 50 μL of this mixture were then combined with Ti(SO_4_)_2_ solution (100 μL, 13.9 × 10^-3^ M), and the absorbance at 405 nm was recorded at 5 min intervals for a duration of 60 min.

For SOD-like assay, the scavenging capability of the AMCB for superoxide anions was evaluated by dissolving 1.0 mg of potassium superoxide (KO_2_) in 1.0 mL of DMSO containing 3.0 mg/mL of 18-crown-6-ether to generate and stabilize •O_2_^-^. Subsequently, AMCB was dispersed into the KO_2_/DMSO solution to a final concentration of 50 μg/mL. After a 5 min reaction period, the remaining •O_2_^-^ was trapped by nitrotetrazolium blue chloride (NBT) in DMSO solution (10 μL, 10 mg/mL). The absorbance of the solution at 680 nm was measured and compared with the initial •O_2_^-^ concentration to ascertain the scavenging efficiency.

*Preparation of FTB and AMCB-FTB Hydrogel**:* TA (0.025 M, 1.0 mL) was mixed with FPBA (0.07 M), and the pH of the solution was adjusted to 8.2. Then, an equal volume of TOB (0.14 M) was added to the mixture. After standing for 10 min, a hydrogel composed of TA, FPBA and TOB was formed and named FTB hydrogel. Meanwhile, TA (0.025 M, 1.0 mL) was mixed with 0.07 M FPBA, and the pH of the solution was adjusted to 8.2. Then, an equal volume of TOB (0.14 M) and 4 μg/mL AMCB was added to the mixture. After standing for 10 min, a hydrogel composed of TA, FPBA, TOB and AMCB was formed and named AMCB-FTB hydrogel.

*Rheological Studies*: The mechanical properties of the hydrogels were characterized by using a multifunctional rotational rheometer. A 20 mm flat plate was employed for the tests, with parameters set at 25°C temperature, angular frequency of 1 Hz, and a strain rate of 1%. Firstly, the modulus-time curves of the FTB hydrogel were obtained. The rheological properties of both FTB and AMCB-FTB were examined using the rheometer at an angular frequency of 1.0 Hz and a strain of 1%. Additionally, the rheological behavior over a range of angular frequencies (0.1 to 100 Hz) was studied with a fixed oscillatory strain of 1%.

*Photothermal Behavior of AMCB-FTB* *Hydrogel**:* To evaluate the photothermal effect, aqueous suspensions of FCB and AMCB-FTB hydrogel were prepared. 200 μL of the suspension was injected into a 96-well microtiter plate and subjected to vertical irradiation with NIR laser (808 nm, 0.8 W/cm^2^) for 10 min. Temperatures were measured at 1 min intervals using an infrared thermal imager. At the end of the laser irradiation, the light source was removed and the temperature continued to be monitored during the subsequent 10 min cooling process to assess the efficiency of the photothermal conversion. To test the photothermal stability of the AMCB-FTB, 10 rounds of irradiation and cooling cycles were performed. The same method was used to test the photothermal properties of Au NPs, AMC and AMCB.

*Responsive Behaviors of the AMCB-FTB* *Hydrogel**:* The AMCB-FTB were exposed to 808 nm (0.8 W/cm^2^) for 10 min to test their temperature responsiveness. The temperature on the AMCB-FTB was recorded using a NIR temperature sensor. For rheological studies, temperature-dependent rheological measurements were investigated as described above, with a gradual change in plate temperature from 25°C to 50°C.

*In Vitro Antibacterial Efficiency:* The antimicrobial efficacy of the Gram-negative bacteria *Escherichia coli* (*E. coli*, ATCC 700927) and *Pseudomonas aeruginosa* (*P. aeruginosa*, CICC 35150) was evaluated *in vitro*. The two bacteria were divided into 12 different treatment groups: PBS only, PBS + NIR, PBS + H_2_O_2_, PBS + NIR + H_2_O_2_, FTB (pure hydrogel), FTB + NIR, FTB + H_2_O_2_, FTB + NIR + H_2_O_2_, AMCB-FTB, AMCB-FTB + NIR, AMCB-FTB + H_2_O_2_, and AMCB-FTB + NIR + H_2_O_2_. After incubation at 37°C for 1 h, 50 µL of each treatment group was evenly spread on agar plates. The number of colonies was determined and the survival rate of bacteria was determined.

*E. coli* or *P. aeruginosa* were treated with PBS, PBS + NIR,PBS + H_2_O_2_, PBS + NIR+H_2_O_2_, PBS + FTB, PBS + FTB + NIR, PBS + FTB + H_2_O_2_, PBS + FTB + NIR + H_2_O_2_, PBS + AMCB-FTB, PBS + AMCB-FTB + NIR, PBS + AMCB-FTB + H_2_O_2_ or PBS + FTB + NIR + H_2_O_2_ for 1 h. DAPI and PI were added to the bacteria, which were incubated at 37°C for 30 min. After incubation, the solutions were washed three times with PBS. Images of the stained bacteria were obtained with the inverted fluorescence microscope from Zeiss, Germany.

*The Detection of Extracellular ROS**:* The mixture of bacteria from the 12 groups was treated with a 2’,7’-dichlorodihydrofluorescein diacetate (DCFH-DA) probe and incubated for 20 min in an incubator at 37°C. After incubation, the solutions were washed three times with PBS, and green fluorescence was detected under an inverted fluorescence microscope.

*Bacterial Cell Membrane Experiment**:* The dissipated membrane potential of *E. coli* and *P. aeruginosa* after different treatments were determined by using 3,3'-Diethyloxacarbocyanine iodide (DiOC_2_(3)). The fluorescent probe 1-N-phenylnaphthylamine (NPN) was also used to determine the changes in bacterial permeability. After different treatments, the supernatant was collected and centrifuged to discard the precipitate. According to the instructions of the BCA kit, BCA working solution was added and incubated at 37°C for 20 min. The leaked proteins were quantitatively analyzed by measuring the absorbance at ∼562 nm.

The morphology of the bacteria after treatment was observed by SEM. The bacteria were treated with glutaraldehyde solution (2.5%) overnight and then dehydrated using different concentrations of ethanol solutions (30%, 50%, 70%, 80%, 90%, 100%). Finally, the obtained solution was dispersed in absolute ethanol to prepare samples.

*In Vitro Antibiofilm Capacity:* The prepared suspensions of *E. coli* and *P. aeruginosa* (1 × 10^8^ CFU/mL) were mixed with 1.0 mL of LB medium and then injected into 24-well culture plates and incubated at 37°C for 24 h to form mature biofilms. For the biofilm destruction experiment, mature biofilms were respectively treated with PBS, PBS + NIR, PBS + H_2_O_2_, PBS + NIR + H_2_O_2_, PBS + FTB, PBS + FTB + NIR, PBS + FTB + H_2_O_2_, PBS + FTB + NIR + H_2_O_2_, PBS + AMCB-FTB, PBS + AMCB-FTB + NIR, PBS + AMCB-FTB + H_2_O_2_ or PBS + FTB + NIR + H_2_O_2_. After 1 h of co-incubation, the mixture was poured off, followed by washing the biofilm with PBS three times. Subsequently, the residual biofilm was stained with crystal violet stain and a viable bacteria count was performed to assess the number of bacteria in the biofilm. In addition, mature biofilms were formed on glass slides and treated under the following conditions: PBS, PBS + NIR, PBS + H_2_O_2_, PBS + NIR+H_2_O_2_, PBS + FTB, PBS + FTB + NIR, PBS + FTB + H_2_O_2_, PBS + FTB + NIR + H_2_O_2_, PBS + AMCB-FTB, PBS + AMCB-FTB + NIR, PBS + AMCB-FTB + H_2_O_2_ or PBS + FTB + NIR + H_2_O_2_. After treatment, the biofilms were stained with SYBR Green I for 30 min and imaged using a laser confocal microscope to obtain their 3D structures.

For the biofilm inhibition test, *E. coli* and *P. aeruginosa* suspensions (1 × 10^8^ CFU/mL) were treated with PBS, PBS + NIR, PBS + H_2_O_2_, PBS + NIR+H_2_O_2_, PBS + FTB, PBS + FTB + NIR, PBS + FTB + H_2_O_2_, PBS + FTB + NIR + H_2_O_2_, PBS + AMCB-FTB, PBS + AMCB-FTB + NIR, PBS + AMCB-FTB + H_2_O_2_ or PBS + FTB + NIR + H_2_O_2_ for 48 h. The inhibitory effect on biofilm was evaluated by crystal violet staining, counting of viable bacteria, and construction of 3D structures.

*Transcriptome Analysis:* *P. aeruginosa* was selected for transcriptome sequencing. The control group included C_1, C_2, and C_3, and the experimental group included T_1, T_2, and T_3. RNA from the treated bacteria was extracted using the Bacterial Total RNA Extraction Kit and the integrity of RNA was assessed. A cDNA library was then constructed and sequenced. Differential gene expression was assessed using the DESeq2 R software package (1.20.0).

*Skin Wound Healing Assay In Vivo:* The effect of FTB and AMCB-FTB hydrogel on wound healing was investigated by constructing a mouse skin wound model infected with *P. aeruginosa*. Eight-week-old BALB/c female mice were used for the experiments. 100 μL of *P. aeruginosa* suspension at a concentration of 1 × 10^8^ CFU/mL was applied to the wound surface and allowed to dry naturally. After 7 days of treatment, skin samples were collected from each group of mice and analyzed histologically by Masson staining and H&E staining. Meanwhile, heart, liver, spleen, lungs and kidneys were stained with H&E and blood samples were collected for comprehensive hematological tests. All animal experiments in this study followed the principles established by the Animal Ethics Committee of Tianjin University of Science & Technology (20240410).

*Statistical Analysis*: Data analysis was performed using GraphPad Prism (Version 9.0.1). Results are expressed as mean ± standard deviation. Comparisons among multiple groups were conducted using one-way or two-way analysis of variance (ANOVA), as appropriate (*p < 0.05, **p < 0.01, ***p < 0.001, ****p < 0.0001).

**2. Results**


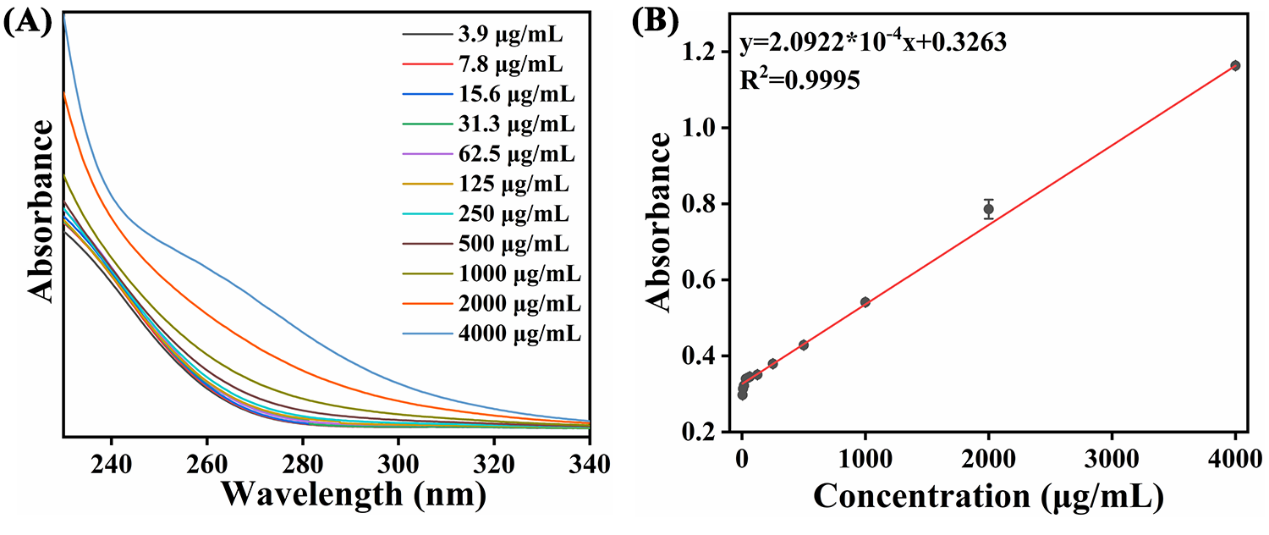


**Figure S1.** (A) The UV-Vis absorption of PMB at different concentrations. (B) The linear relationship between PMB concentration and absorption intensity.


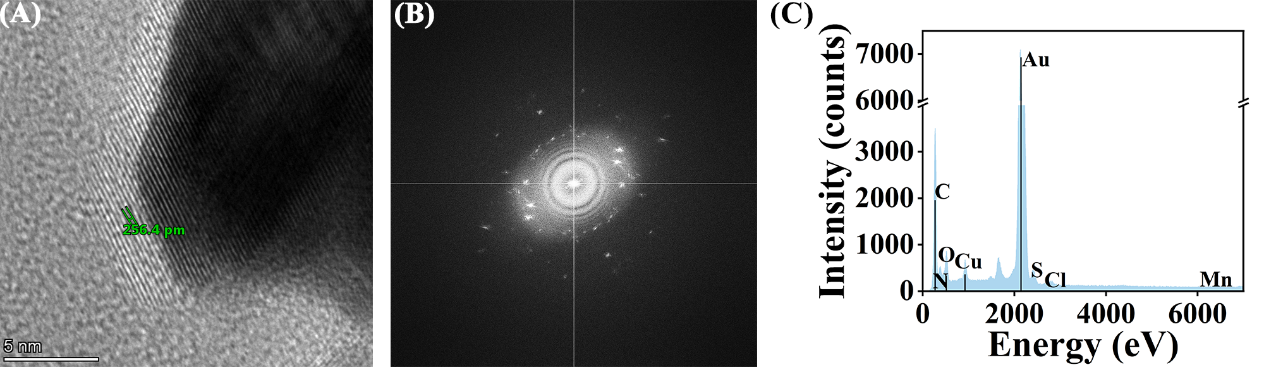


**Figure S2.** The lattice (A), SAED patterns (B), and EDS analysis (C) of AMCB.


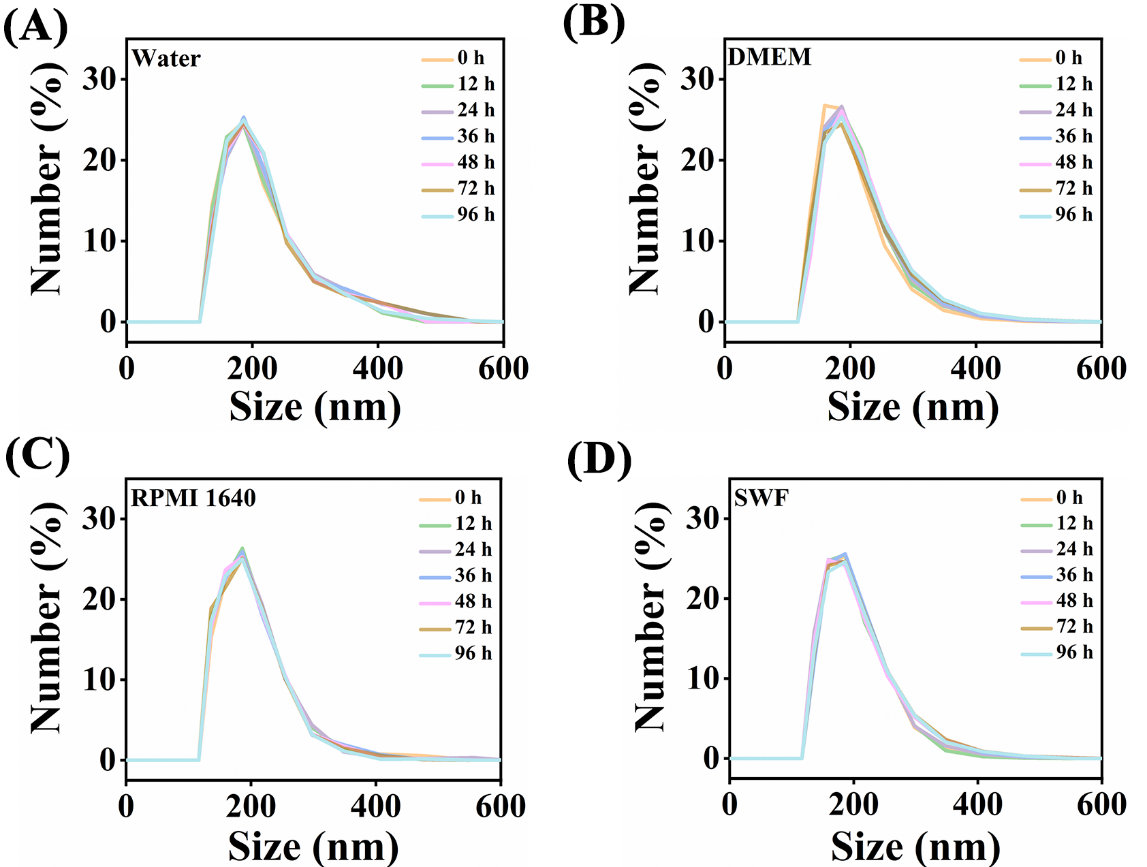


**Figure S3.** The particle size distribution of AMCB in water (A), DMEM (B), RPMI 1640 (C), and simulated wound fluid (SWF) (D) after different incubation time.

**
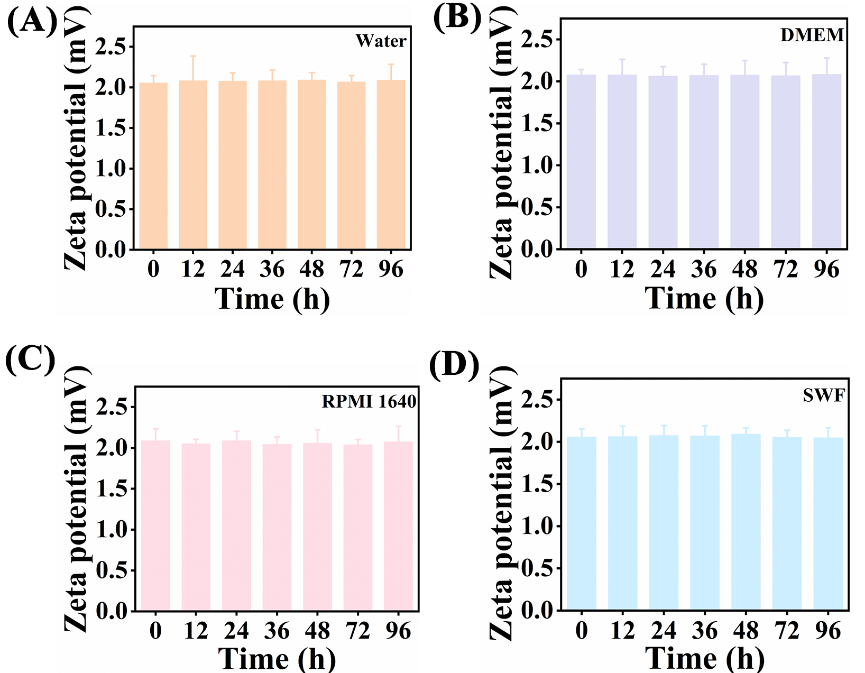
**

**Figure S4.** Zeta potential of AMCB in water (A), DMEM (B), RPMI 1640 (C), and simulated wound fluid (SWF) (D) after different incubation time. Data are presented as mean ± SD (n = 3, independent measurements).


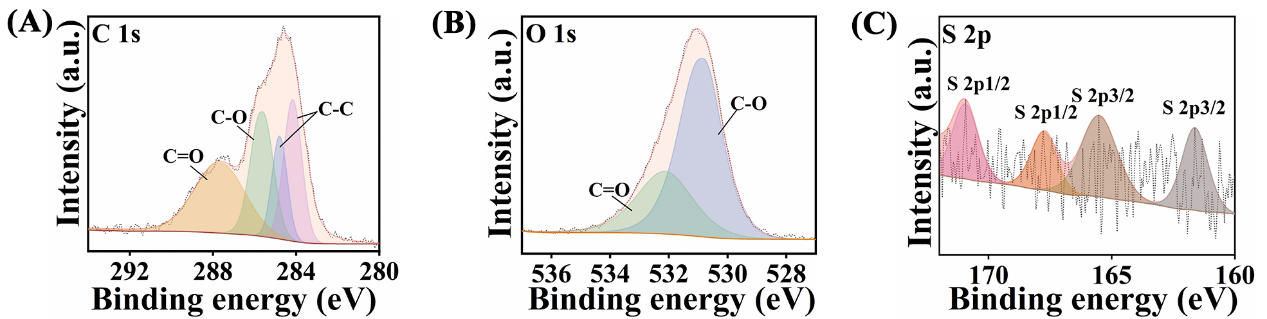


**Figure S5.** High-resolution XPS spectra of (A) C 1s, (B) O 1s and (C) S 2p orbits for AMCB.


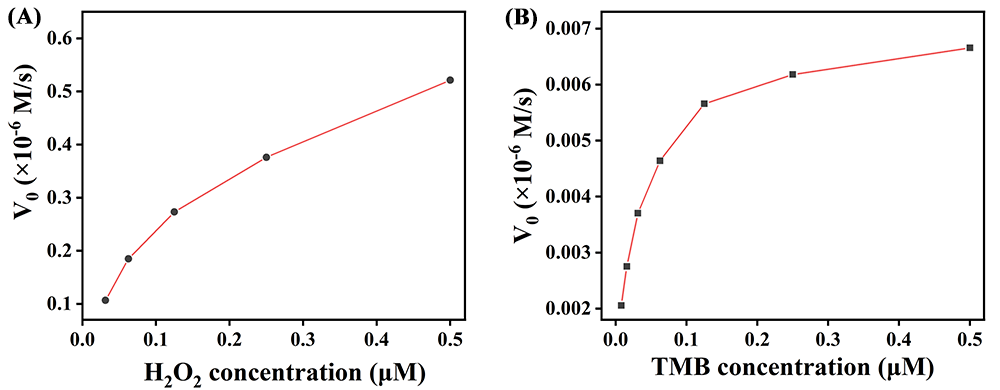


**Figure S6.** Steady-state kinetics curves of AMCB nanozymes for (A) H_2_O_2_ and (B) TMB.


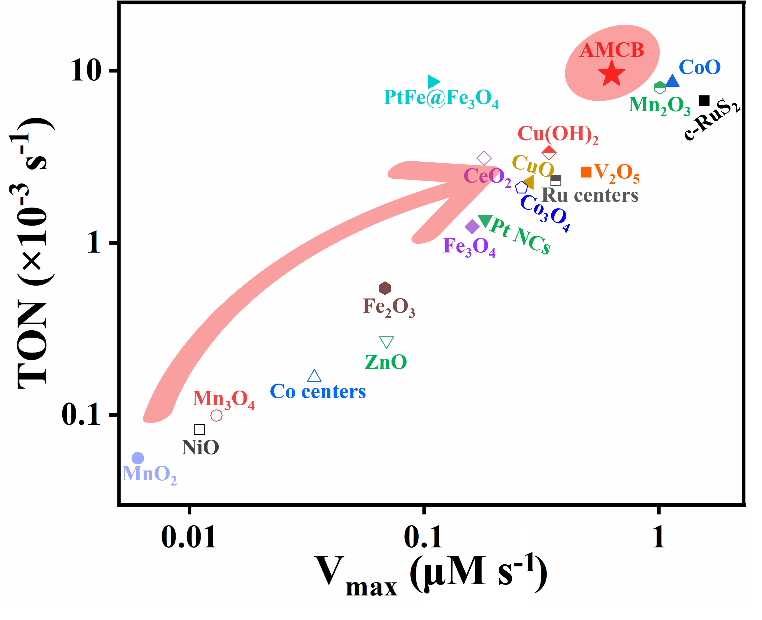


**Figure S7.** Comparison of the ROS-related catalytic performance index on POD-like activity of our synthesized materials with other reported metallic oxides and metal nanoparticles-based biocatalysts.


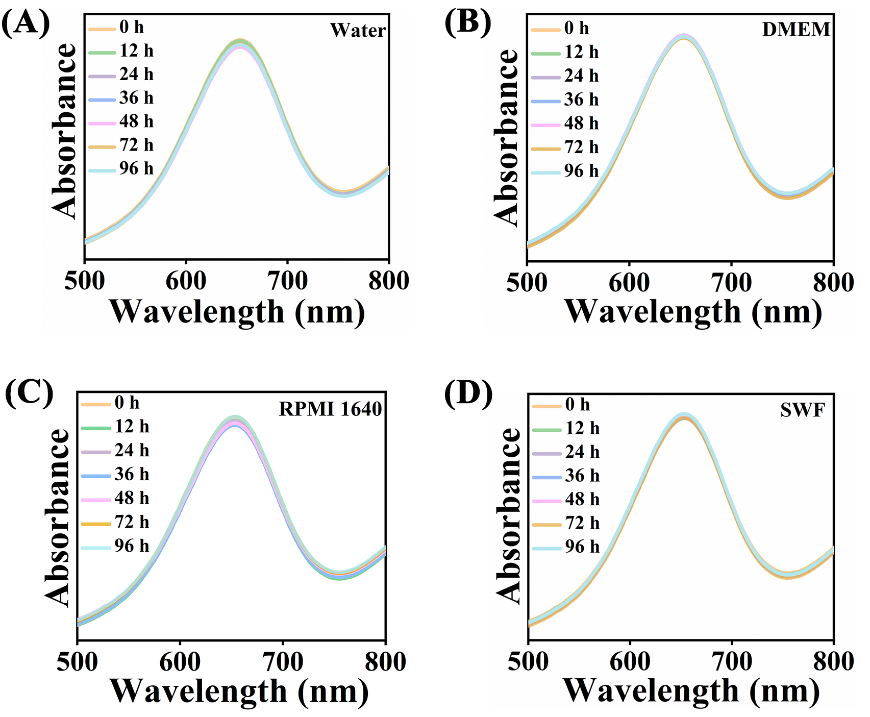


**Figure S8.** Peroxidase (POD)-like activity of AMCB in water (A), DMEM (B), RPMI 1640 (C), and simulated wound fluid (SWF) (D) after different incubation time.


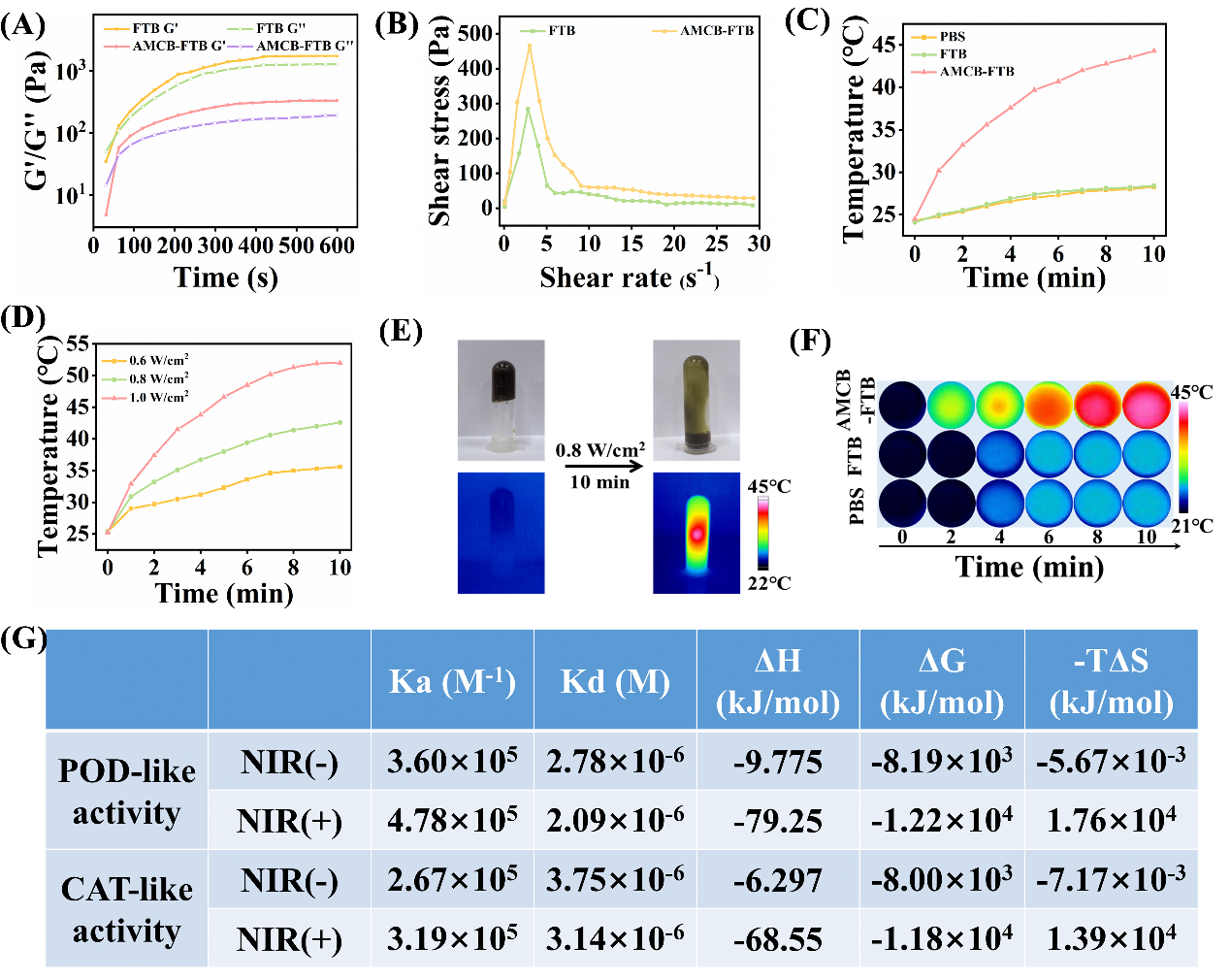


**Figure S9.** (A) FTB and AMCB-FTB modulus-time dependent rheological diagrams. (B) Shear stress and shear rate curves of FTB and AMCB-FTB. (C) Photothermal curves of FTB and AMCB-FTB in the presence of an 808 nm laser. (D) Photothermal curves of AMCB-FTB under laser irradiation at different power densities. (E) Digital and temperature images of sample bottles containing AMCB-FTB before and after heating. (F) Real-time thermal imaging of FTB and AMCB-FTB in the presence of an 808 nm laser. (G) Thermodynamic parameters (Ka, Kd, ΔH, ΔG, -TΔS) derived from ITC fitting.


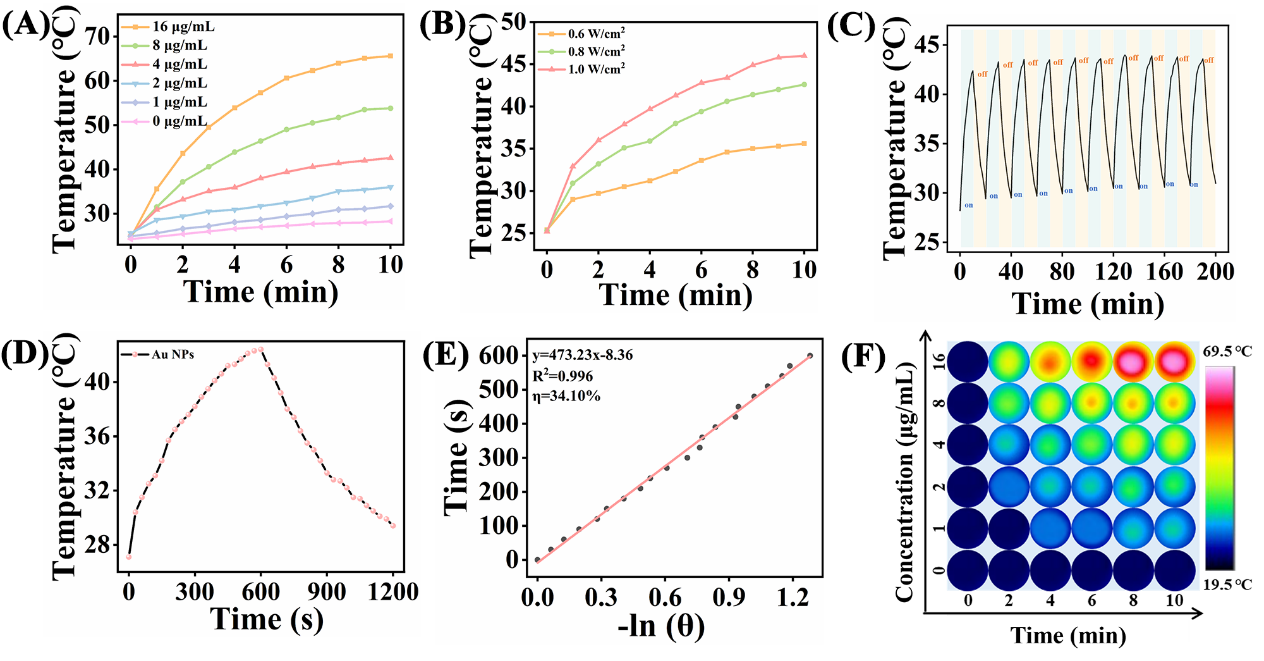


**Figure S10.** Photothermal heating performances of Au NPs. (A) Photothermal curves of Au NPs with different concentrations in the presence of an 808 nm laser. (B) Photothermal curves of Au NPs with different power. (C) Photothermal stability of Au NPs under successive 10 cycles of on/off laser irradiation. (D) The temperature profile of Au NPs after exposure to 808 nm laser irradiation for single on/off cycle. (E) Cooling time vs -lnθ obtained from cooling period in (D). (F) Real-time thermal imaging of different concentrations of Au NPs in the presence of an 808 nm laser.


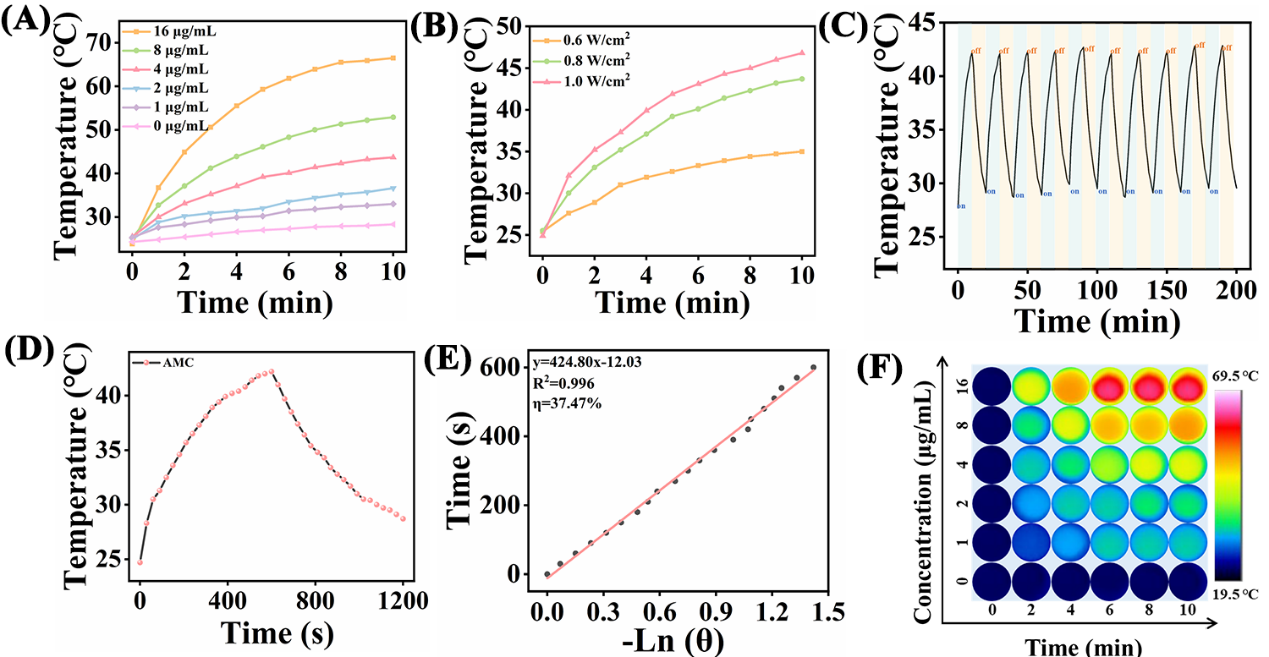


**Figure S11.** Photothermal heating performances of AMC. (A) Photothermal curves of AMC with different concentrations in the presence of an 808 nm laser. (B) Photothermal curves of AMC with different power. (C) Photothermal stability of AMC under successive 10 cycles of on/off laser irradiation. (D) The temperature profile of AMC after exposure to 808 nm laser irradiation for single on/off cycle. (E) Cooling time vs -lnθ obtained from cooling period in (D). (F) Real-time thermal imaging of different concentrations of AMC in the presence of an 808 nm laser.


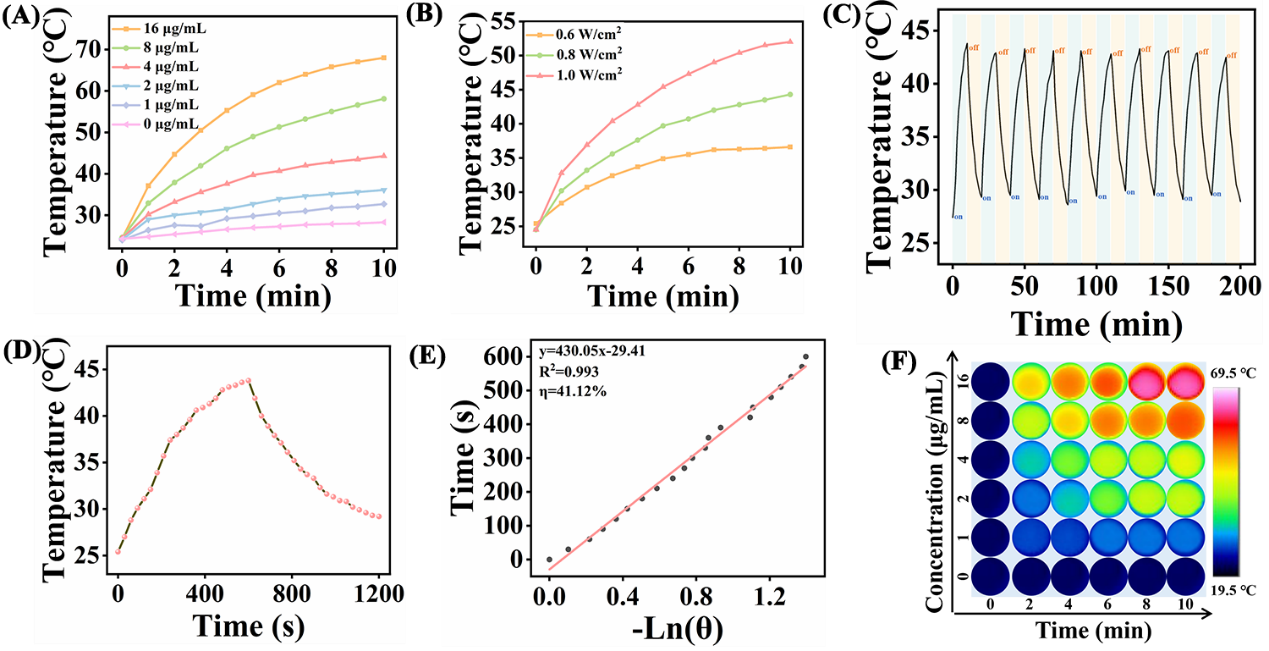


**Figure S12.** Photothermal heating performances of AMCB. (A) Photothermal curves of AMCB with different concentrations in the presence of an 808 nm laser. (B) Photothermal curves of AMCB with different power. (C) Photothermal stability of AMCB under successive 10 cycles of on/off laser irradiation. (D) The temperature profile of AMCB after exposure to 808 nm laser irradiation for single on/off cycle. (E) Cooling time vs -lnθ obtained from cooling period in (D). (F) Real-time thermal imaging of different concentrations of AMCB in the presence of an 808 nm laser.
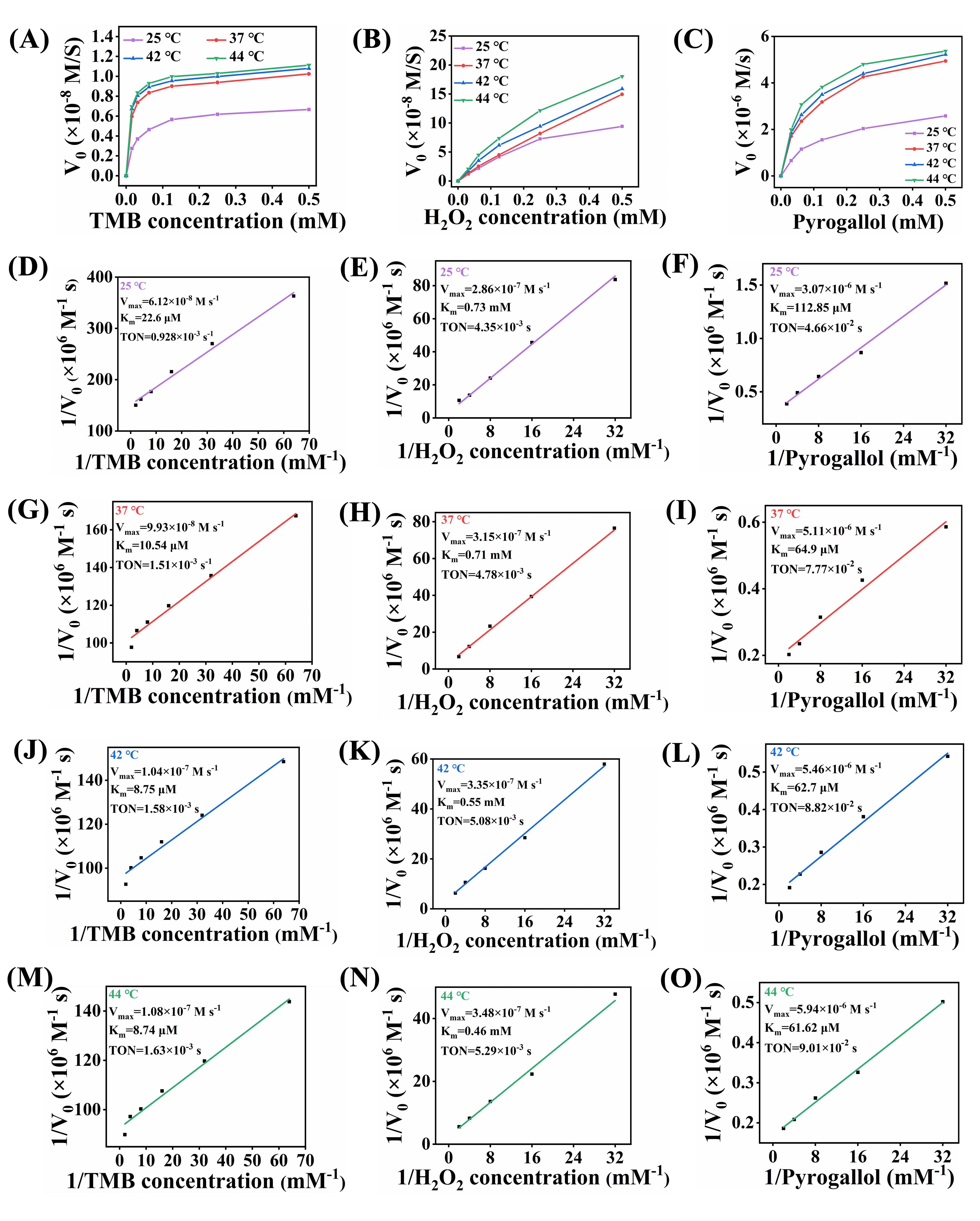


**Figure S13. Temperature-dependent enzymatic kinetics of AMCB.** Steady-state kinetic curves of AMCB at different temperatures (25, 37, 42, and 44°C) for TMB (A), H_2_O_2_ (B), and pyrogallol (C). (D-O) Lineweaver–Burk plots of AMCB at different temperatures (25, 37, 42, and 44°C) using TMB (substrate for POD-like activity), H_2_O_2_ (substrate for CAT-like activity), and pyrogallol (substrate for SOD-like activity).


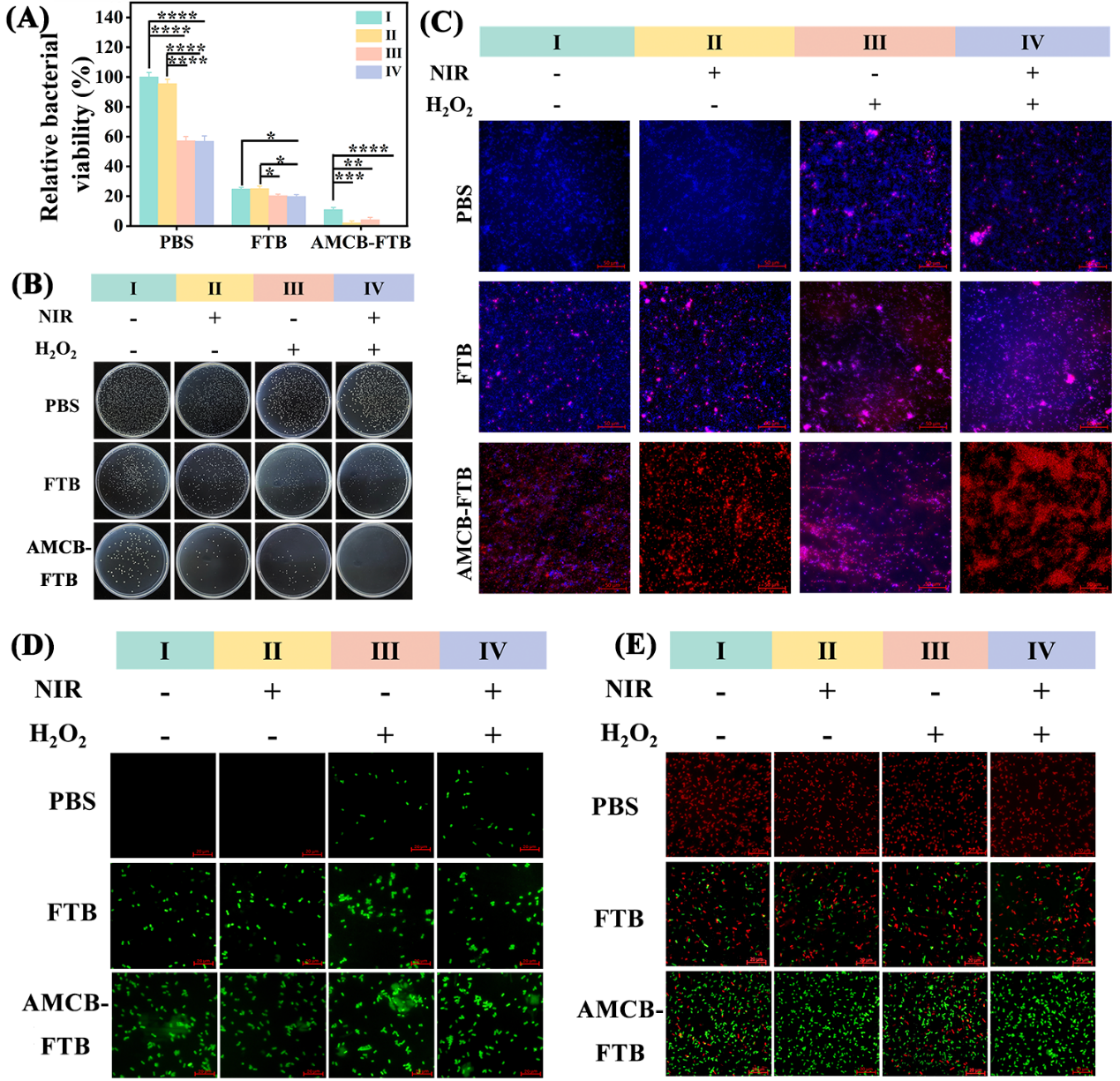


**Figure S14.** (A) The corresponding relative bacterial viability of *E. coli.* (B) Colonies growth of *E. coli* after different treatments on agar plates. (C) Live/dead bacterial cells staining images of *E. coli* after different treatments. (D) Fluorescence images of *E. coli* stained with DCFH-DA after different treatments. (E) Membrane potentials of *E. coli* after different treatments were stained with the fluorescent dye DiOC_2_(3). I: Treatments without NIR and H_2_O_2_. Ⅱ: Treatments with NIR but without H_2_O_2_. Ⅲ: Treatments without NIR but with H_2_O_2_. Ⅳ: Treatments with NIR and H_2_O_2_. Data are presented as mean ± SD (n = 3). Statistical significance was tested with two-way ANOVA, *p < 0.05, **p < 0.01, ***p < 0.001, ****p < 0.0001.


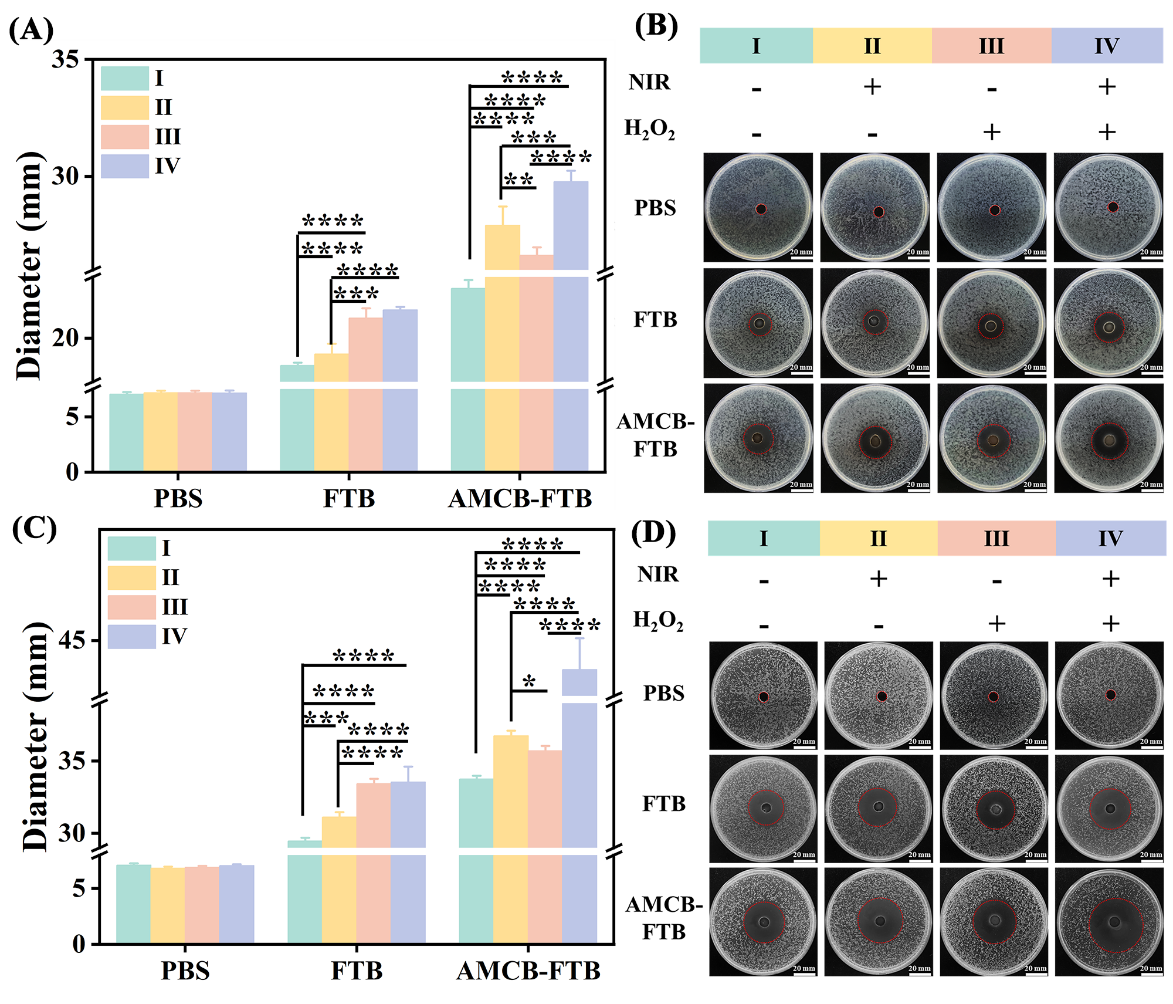


**Figure S15.** The diameters (A) and images (B) of *P. aeruginosa* treated with different treatment. The diameters (C) and images (D) of *E. coli* treated with different treatment. I: Treatments without NIR and H_2_O_2_. Ⅱ: Treatments with NIR but without H_2_O_2_. Ⅲ: Treatments without NIR but with H_2_O_2_. Ⅳ: Treatments with NIR and H_2_O_2_. Data are presented as mean ± SD (n = 3). Statistical significance was tested with two-way ANOVA, *p < 0.05, **p < 0.01, ***p < 0.001, ****p < 0.0001.


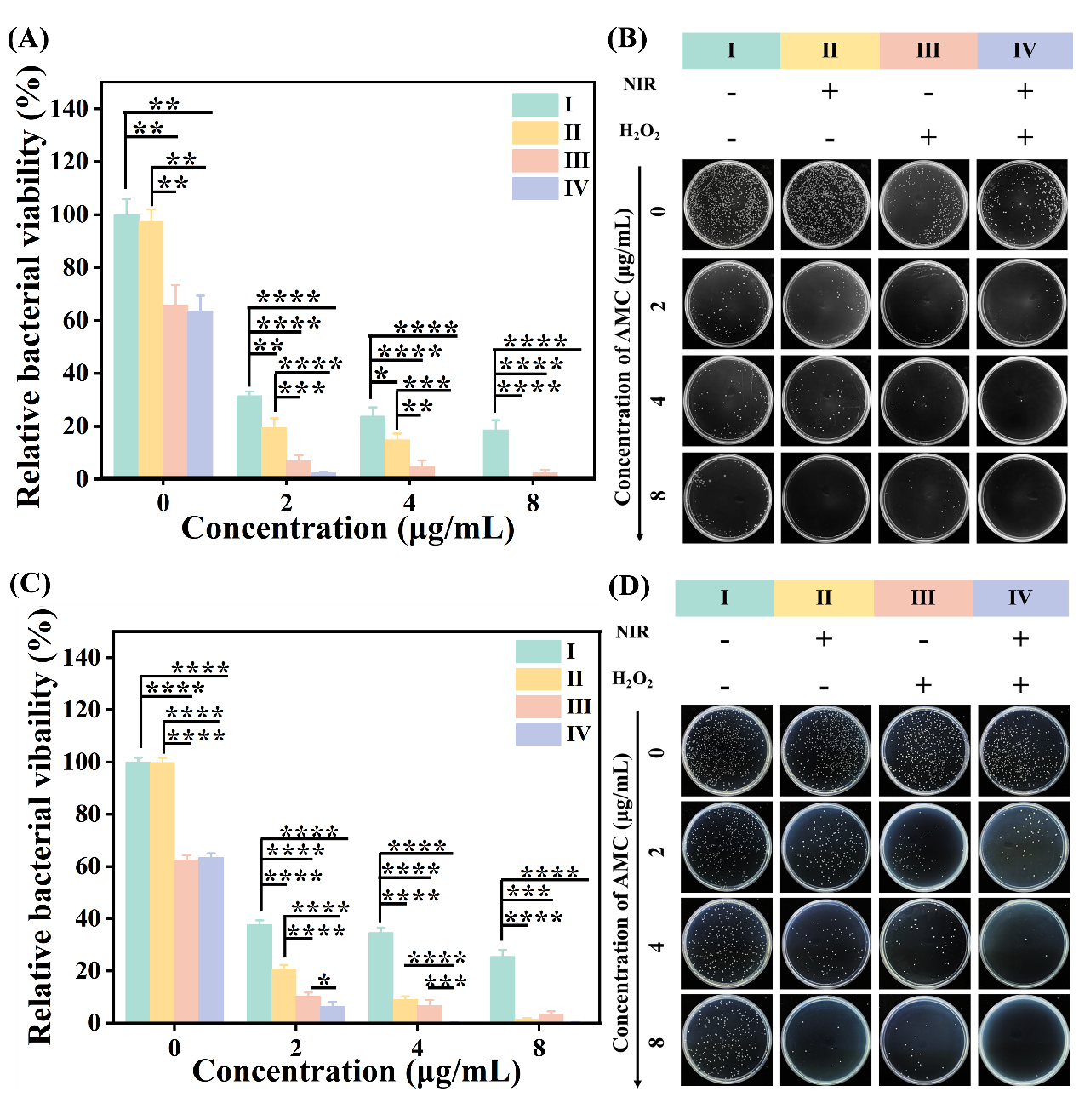


**Figure S16.** The corresponding relative bacterial viability of *P. aeruginosa* (A) and *E. coli* (C) treated with different concentration AMC. Colonies growth of *P. aeruginosa* (B) and *E. coli* (D) after different concentration AMC treatments on agar plates. I: Treatments without NIR and H_2_O_2_. Ⅱ: Treatments with NIR but without H_2_O_2_. Ⅲ: Treatments without NIR but with H_2_O_2_. Ⅳ: Treatments with NIR and H_2_O_2_. Data are presented as mean ± SD (n = 3). Statistical significance was tested with two-way ANOVA, *p < 0.05, **p < 0.01, ***p < 0.001, ****p < 0.0001.


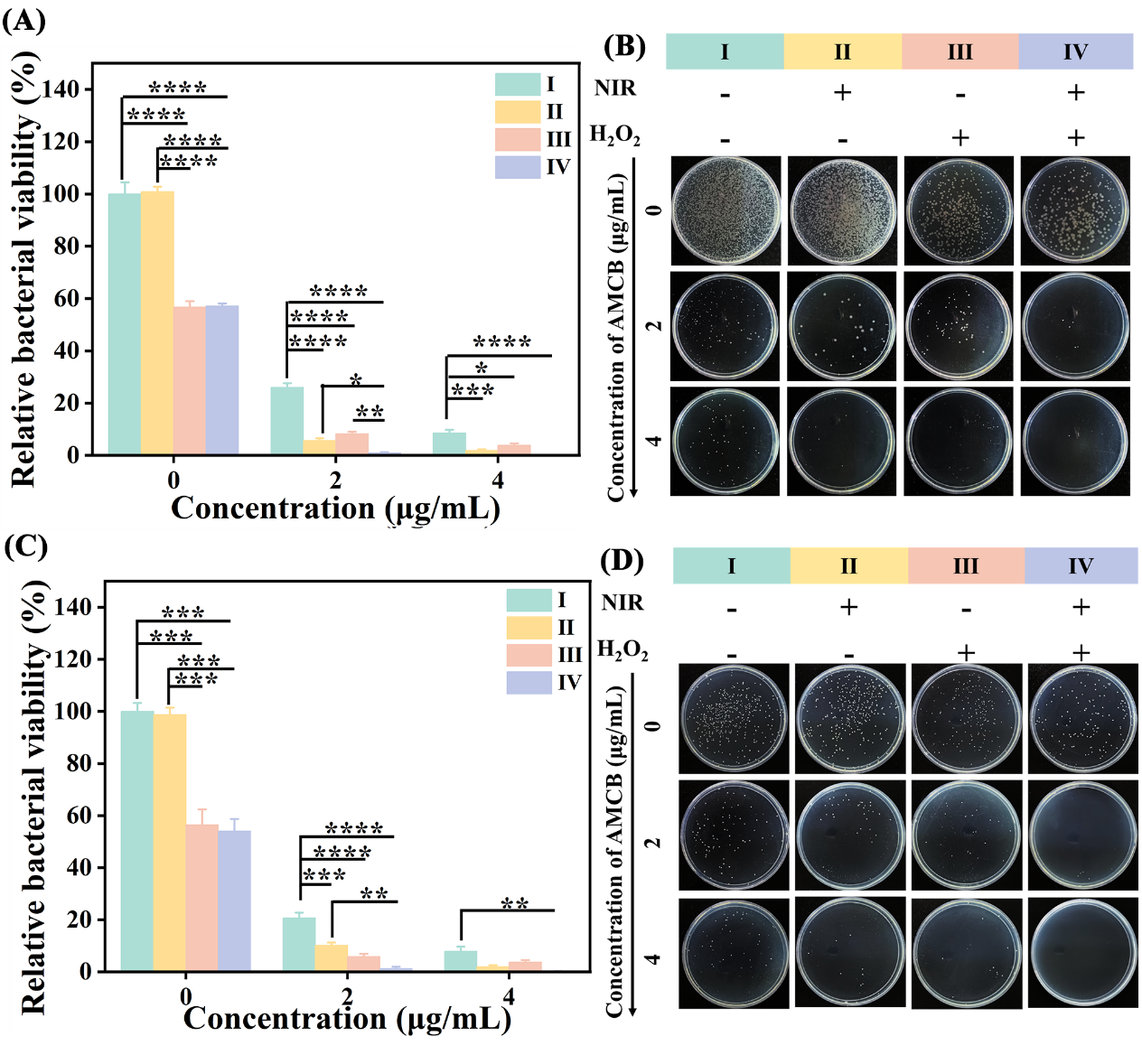


**Figure S17.** The corresponding relative bacterial viability of *P. aeruginosa* (A) and *E. coli* (C) treated with different concentration AMCB. Colonies growth of *P. aeruginosa* (B) and *E. coli* (D) after different concentration AMCB treatments on agar plates. I: Treatments without NIR and H_2_O_2_. Ⅱ: Treatments with NIR but without H_2_O_2_. Ⅲ: Treatments without NIR but with H_2_O_2_. Ⅳ: Treatments with NIR and H_2_O_2_. Data are presented as mean ± SD (n = 3). Statistical significance was tested with two-way ANOVA, *p < 0.05, **p < 0.01, ***p < 0.001, ****p < 0.0001.


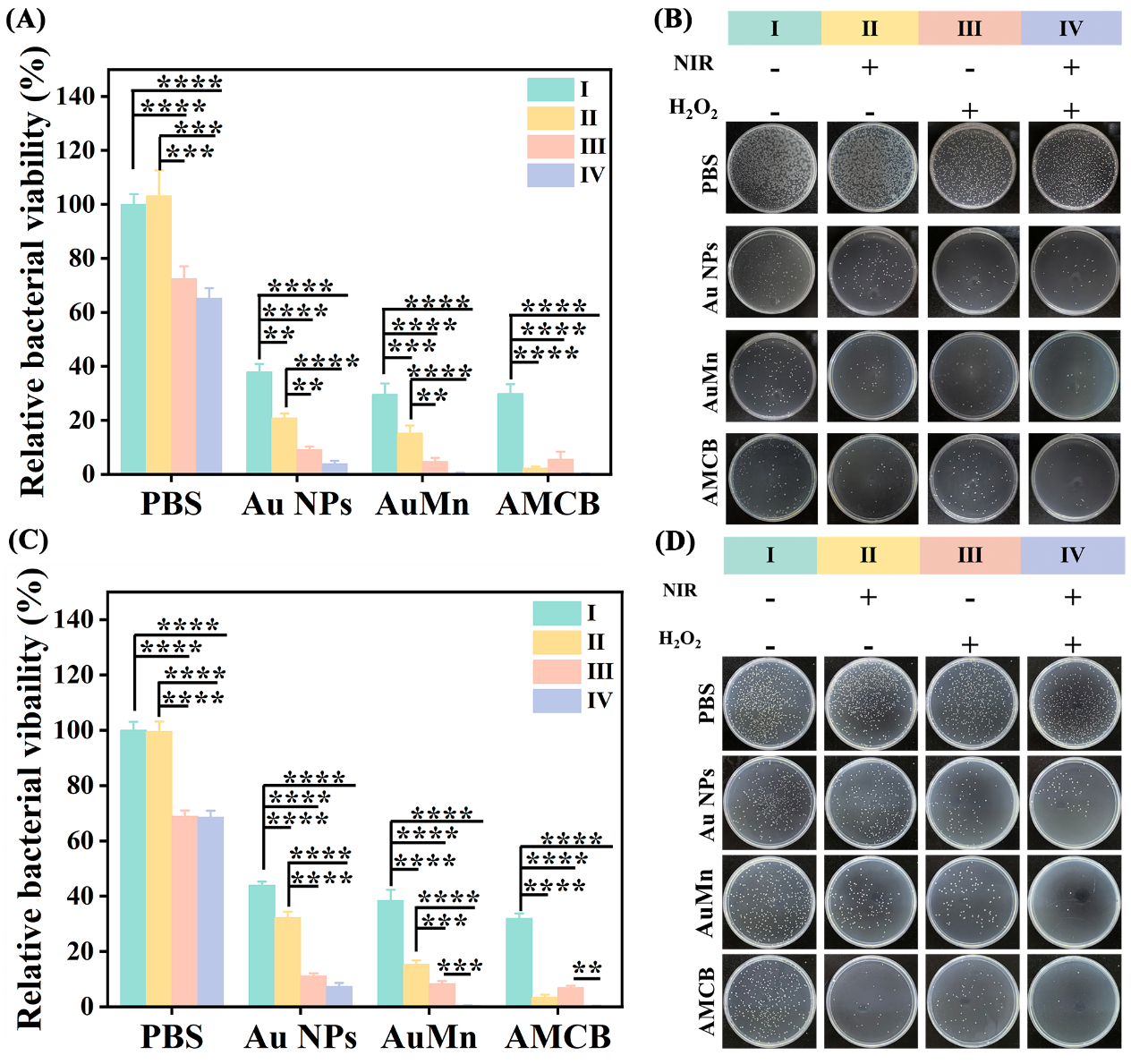


**Figure S18.** The corresponding relative bacterial viability of *P. aeruginosa* (A) and *E. coli* (C) treated with Au NPs, AuMn and AMCB. Colonies growth of *P. aeruginosa* (B) and *E. coli* (D) after treatments with Au NPs, AuMn and AMCB on agar plates. I: Treatments without NIR and H_2_O_2_. Ⅱ: Treatments with NIR but without H_2_O_2_. Ⅲ: Treatments without NIR but with H_2_O_2_. Ⅳ: Treatments with NIR and H_2_O_2_. Data are presented as mean ± SD (n = 3). Statistical significance was tested with two-way ANOVA, **p < 0.01, ***p < 0.001, ****p < 0.0001.


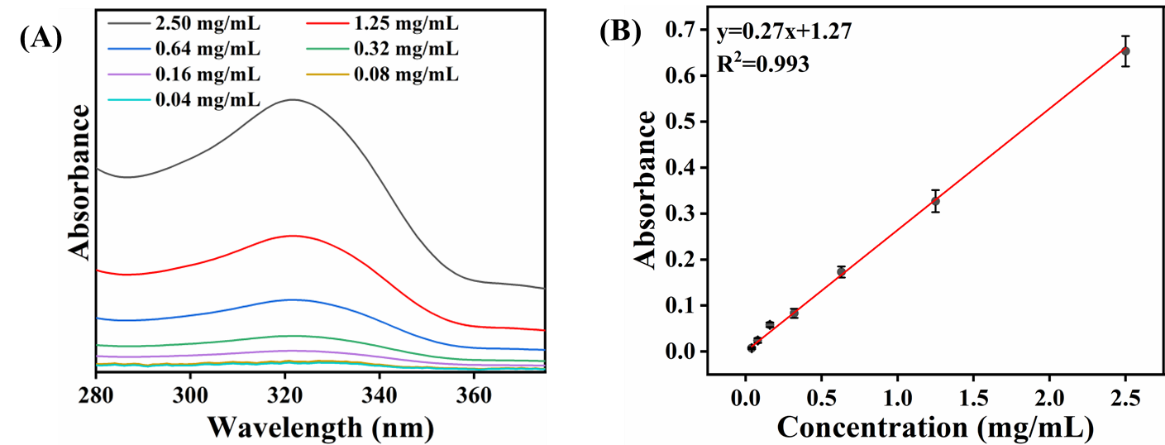


**Figure S19.** (A) The UV-Vis absorption of TA at different concentrations. (B) The linear relationship between TA concentration and absorption intensity.


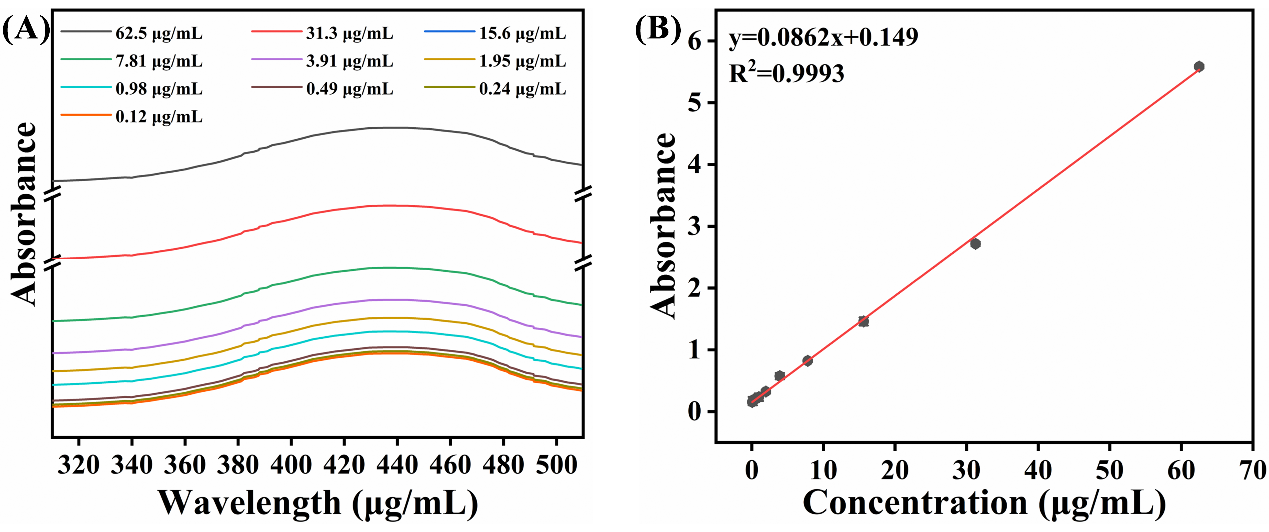


**Figure S20.** (A) The UV-Vis absorption of TOB at different concentrations. (B) The linear relationship between TOB concentration and absorption intensity.


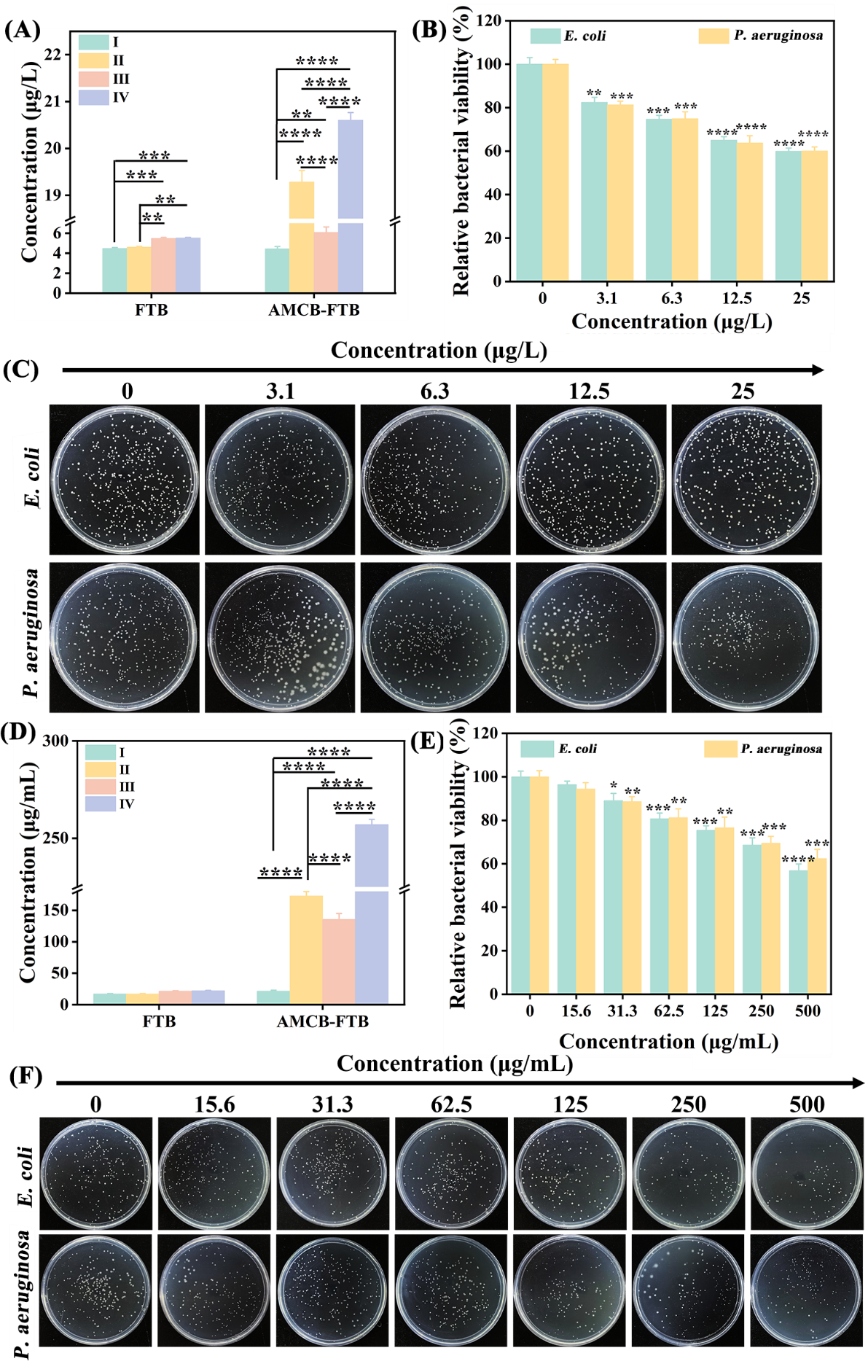


**Figure S21.** In *vitro* release of TOB (A) and TA (D) from FTB and AMCB-FTB under different process. Data are presented as mean ± SD (n = 3). Statistical significance was tested with two-way ANOVA, **p < 0.01, ***p < 0.001, ****p < 0.0001. The corresponding relative bacterial viability of *P. aeruginosa* and *E. coli* that treated with different concentrations TOB (B) or TA (E)*.* Data are presented as mean ± SD (n = 3). Statistical significance was tested with one-way ANOVA, *p < 0.05, **p < 0.01, ***p < 0.001, ****p < 0.0001. Colonies growth of *P. aeruginosa* (C) and *E. coli* (F) after different concentrations of TOB or TA on agar plates. I: Treatments without NIR and H_2_O_2_. Ⅱ: Treatments with NIR but without H_2_O_2_. Ⅲ: Treatments without NIR but with H_2_O_2_. Ⅳ: Treatments with NIR and H_2_O_2_.


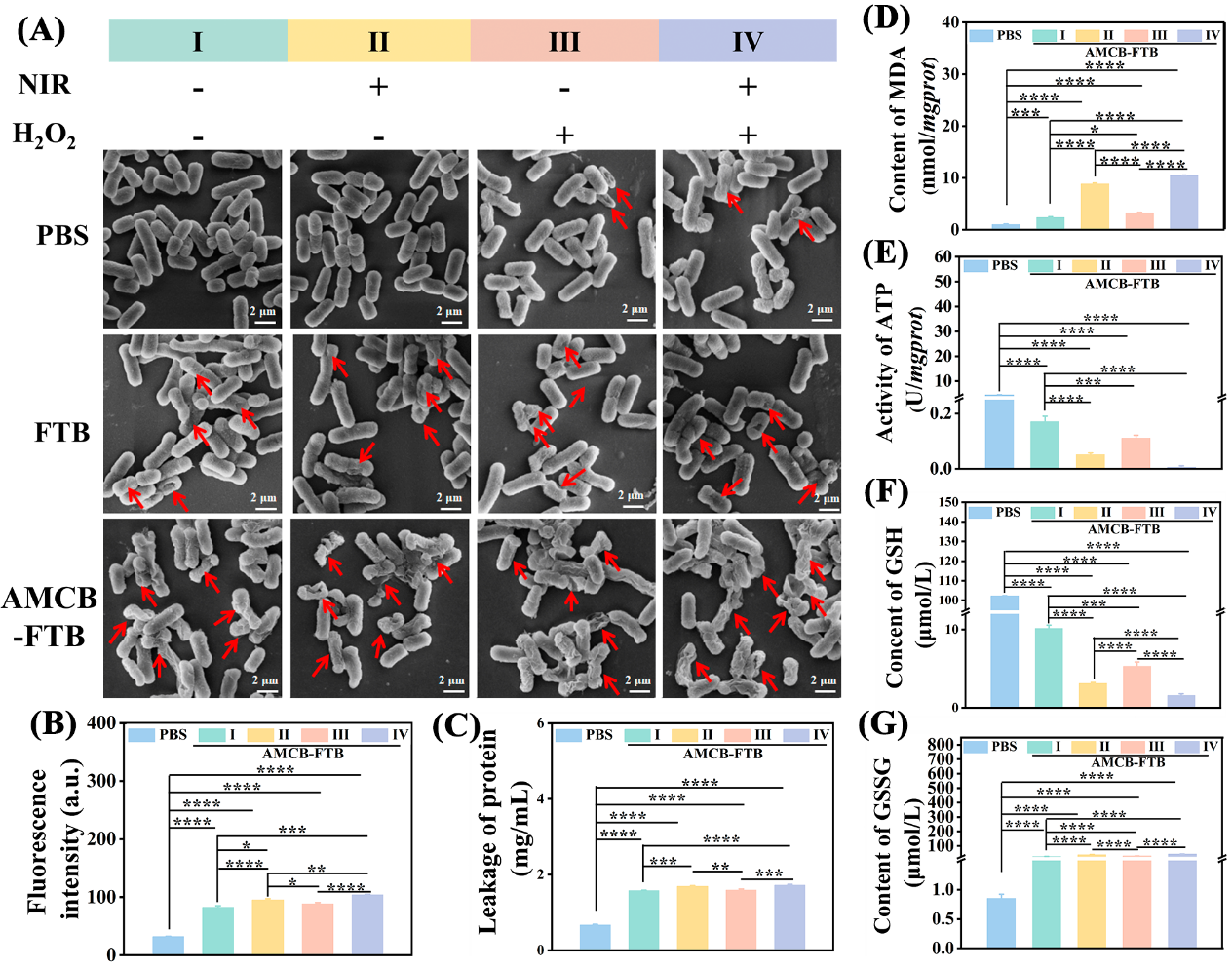


**Figure S22.** (A) SEM images of *E. coli* after different treatments. Membrane permeability (B) and protein leakage (C) of *E. coli* after different treatments. The content of MDA (D), GSH (F) and GSSG (G) and activity of ATP (E) of *E. coli* after different treatments. I: Treatments without NIR and H_2_O_2_. Ⅱ: Treatments with NIR but without H_2_O_2_. Ⅲ: Treatments without NIR but with H_2_O_2_. Ⅳ: Treatments with NIR and H_2_O_2_. Data are presented as mean ± SD (n = 3). Statistical significance was tested with one-way ANOVA, *p < 0.05, **p < 0.01, ***p < 0.001, ****p < 0.0001.


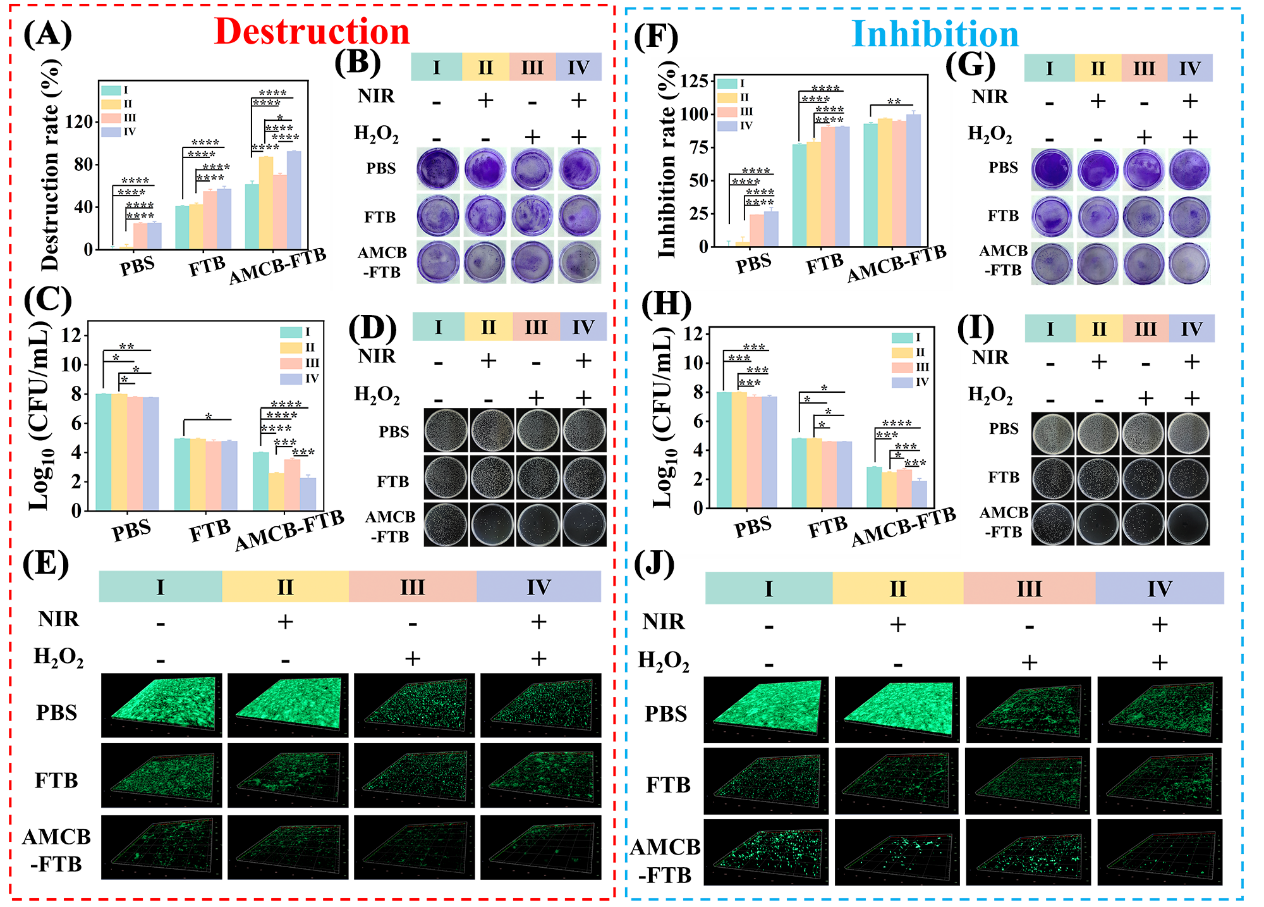


**Figure S23.** (A) Destruction rate of *E. coli* mature biofilm in each group. (B) Crystalline violet staining images of mature biofilms under different treatment conditions. (C) CFU count of *E. coli* mature biofilms in different groups. (D) The agar plate photographs of *E. coli* mature biofilms in different groups. (E) 3D structural images of mature biofilms of *E. coli* with different treatments. (F) Inhibition rate of *E. coli* biofilm formation in each group. (G) Crystalline violet staining images of immature biofilms under different treatment conditions. (H) CFU count of *E. coli* immature biofilms in different groups. (I) The agar plate photographs of *E. coli* immature biofilms in different groups. (J) 3D structural images of immature biofilms of *E. coli* with different treatments. I: Treatments without NIR and H_2_O_2_. Ⅱ: Treatments with NIR but without H_2_O_2_. Ⅲ: Treatments without NIR but with H_2_O_2_. Ⅳ: Treatments with NIR and H_2_O_2_. Data are presented as mean ± SD (n = 3). Statistical significance was tested with two-way ANOVA, *p < 0.05, **p < 0.01, ***p < 0.001, ****p < 0.0001.


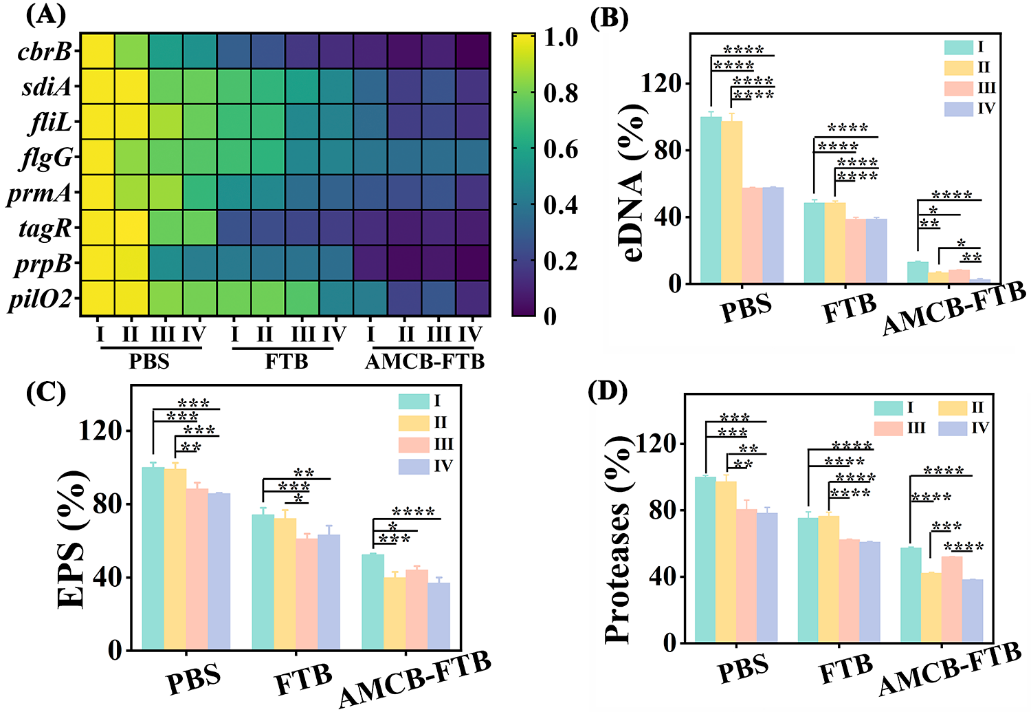


**Figure S24.** (A) RT-qPCR analysis of the relative expressions of *cbrB*, *sdiA*, *fliL*, *flgG*, *prmA*, *tagR*, *prpB* and *pilO2* in *P. aeruginosa* after different treatments. Effects of the different treatments on eDNA production (B), EPS production (C) and proteases production (D) of *P. aeruginosa* biofilm. I: Treatments without NIR and H_2_O_2_. Ⅱ: Treatments with NIR but without H_2_O_2_. Ⅲ: Treatments without NIR but with H_2_O_2_. Ⅳ: Treatments with NIR and H_2_O_2_. Data are presented as mean ± SD (n = 3). Statistical significance was tested with two-way ANOVA, *p < 0.05, **p < 0.01, ***p < 0.001, ****p < 0.0001.


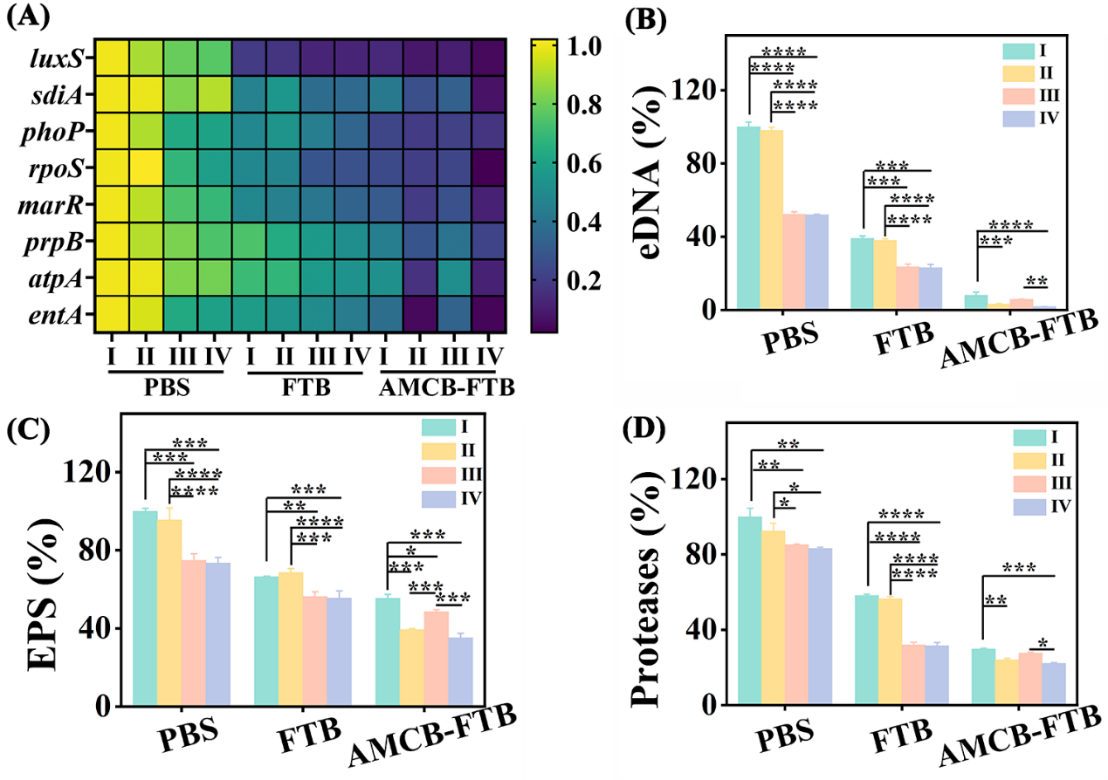


**Figure S25.** (A) RT-qPCR analysis of the relative expressions of *luxS*, *phoP*, *sdiA*, *rpoS*, *marR*, *prpB*, *entA* and *atpA* in *E. coli* after different treatments. Effects of the different treatments on eDNA production (B), EPS production (C) and proteases production (D) of *E. coli* biofilm. I: Treatments without NIR and H_2_O_2_. Ⅱ: Treatments with NIR but without H_2_O_2_. Ⅲ: Treatments without NIR but with H_2_O_2_. Ⅳ: Treatments with NIR and H_2_O_2_. Data are presented as mean ± SD (n = 3). Statistical significance was tested with two-way ANOVA, *p < 0.05, **p < 0.01, ***p < 0.001, ****p < 0.0001.


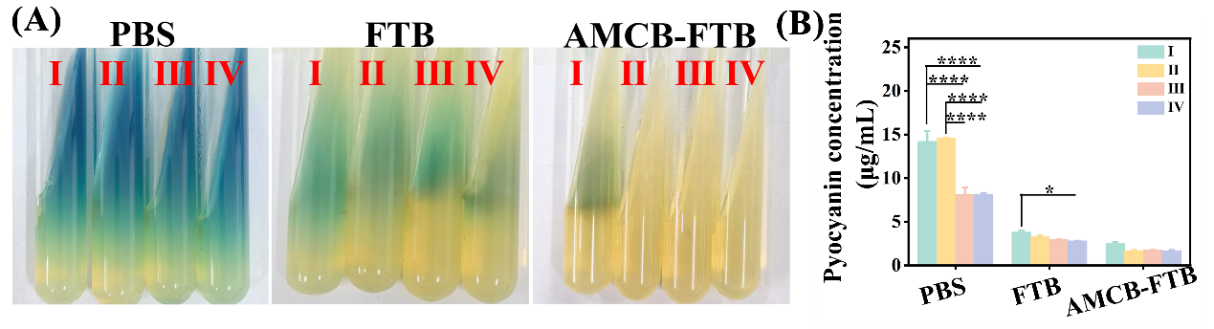


**Figure S26.** (A) Photographs of changes in the production of chlorotoxin by *P. aeruginosa* after different treatments. (B) Changes in the amount of chlorotoxin produced by *P. aeruginosa* after different treatments. I: Treatments without NIR and H_2_O_2_. Ⅱ: Treatments with NIR but without H_2_O_2_. Ⅲ: Treatments without NIR but with H_2_O_2_. Ⅳ: Treatments with NIR and H_2_O_2_. Data are presented as mean ± SD (n = 3). Statistical significance was tested with two-way ANOVA, *p < 0.05, ****p < 0.0001.


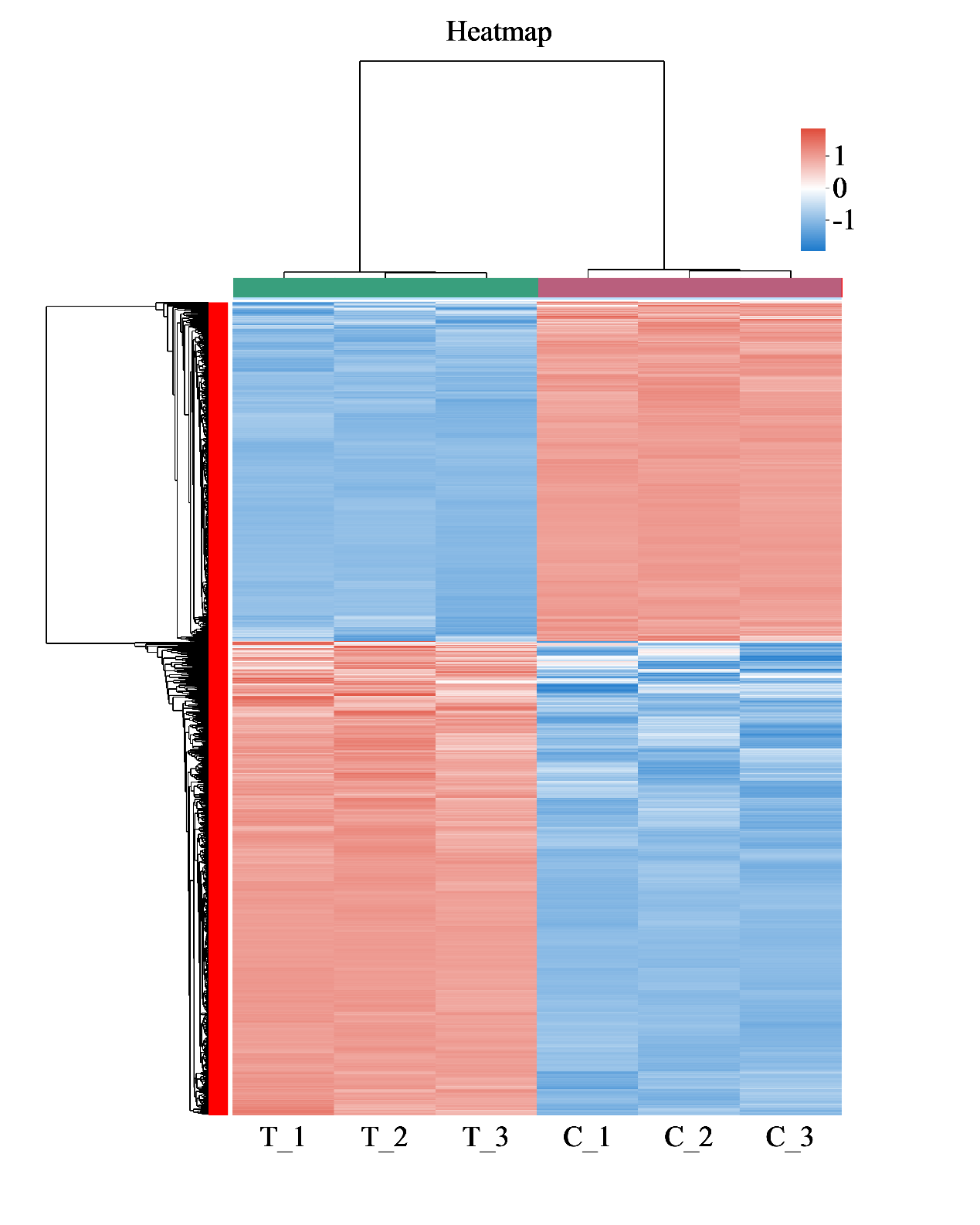


**Figure S27.** Cluster analysis of DEGs showing gene expression patterns. Red: up-regulation; blue: down-regulation. C_1-C_3: Control; T_1-T_3: AMCB-FTB+ NIR + H_2_O_2_ treatment group.


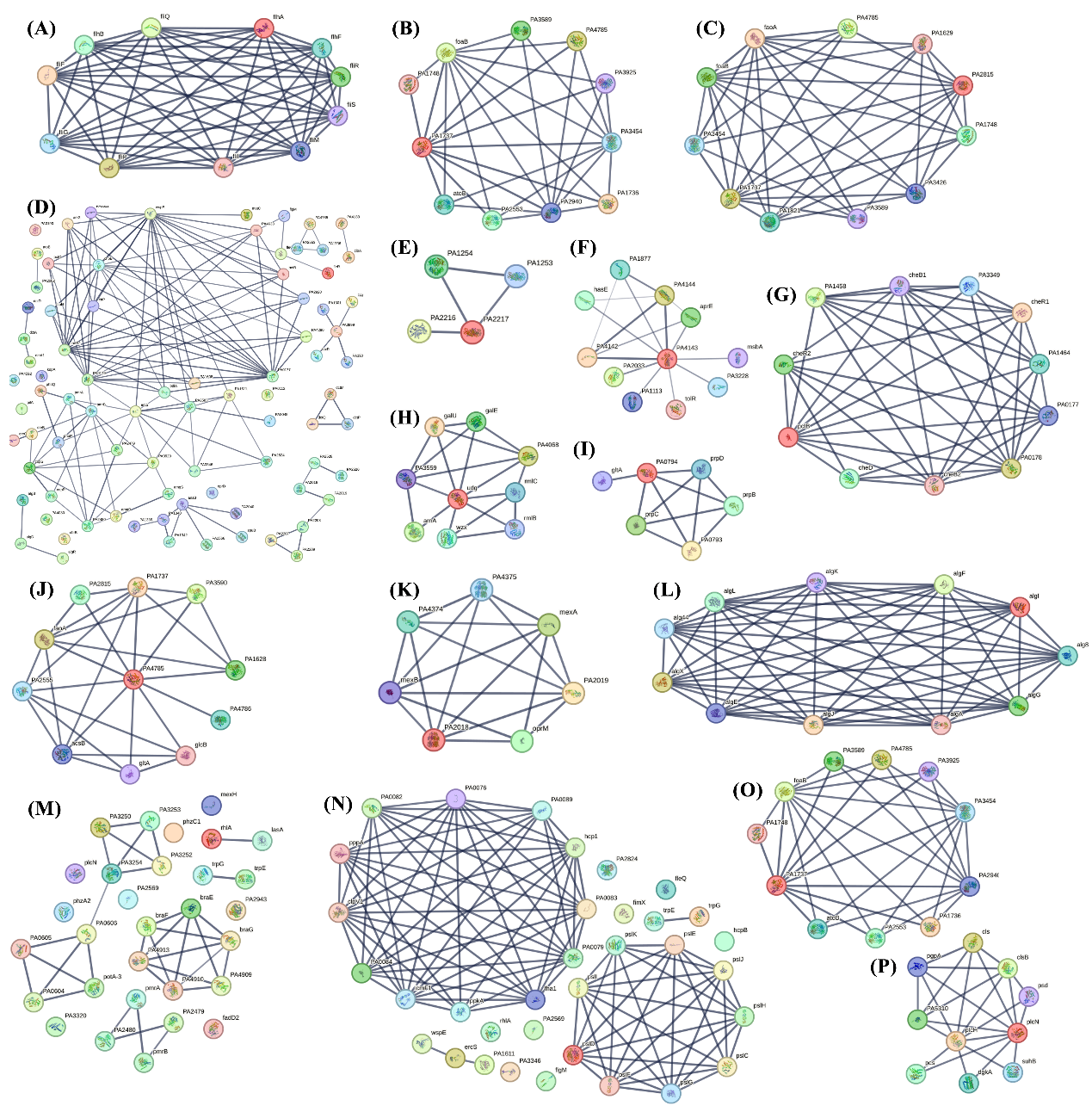


**Figure S28.** Protein-protein interaction network of DEGs in *P. aeruginosa*. (A) Valine, leucine and isoleucine degradation. (B) Flagellar assembly. (C) Fatty acid degradation. (D) Two-component system. (E) ABC transporters. (F) Pentose and glucuronate interconversions. (G) Bacterial chemotaxis. (H) Propanoate metabolism. (I) O-Antigen nucleotide sugar biosynthesis. (J) beta-Lactam resistance. (K) Fat digestion and absorption. (L) Exopolysaccharide biosynthesis. (M) Quorum sensing. (N) Inositol phosphate metabolism. (O) Caprolactam degradation. (P) Biofilm formation-*P. aeruginosa.*

*
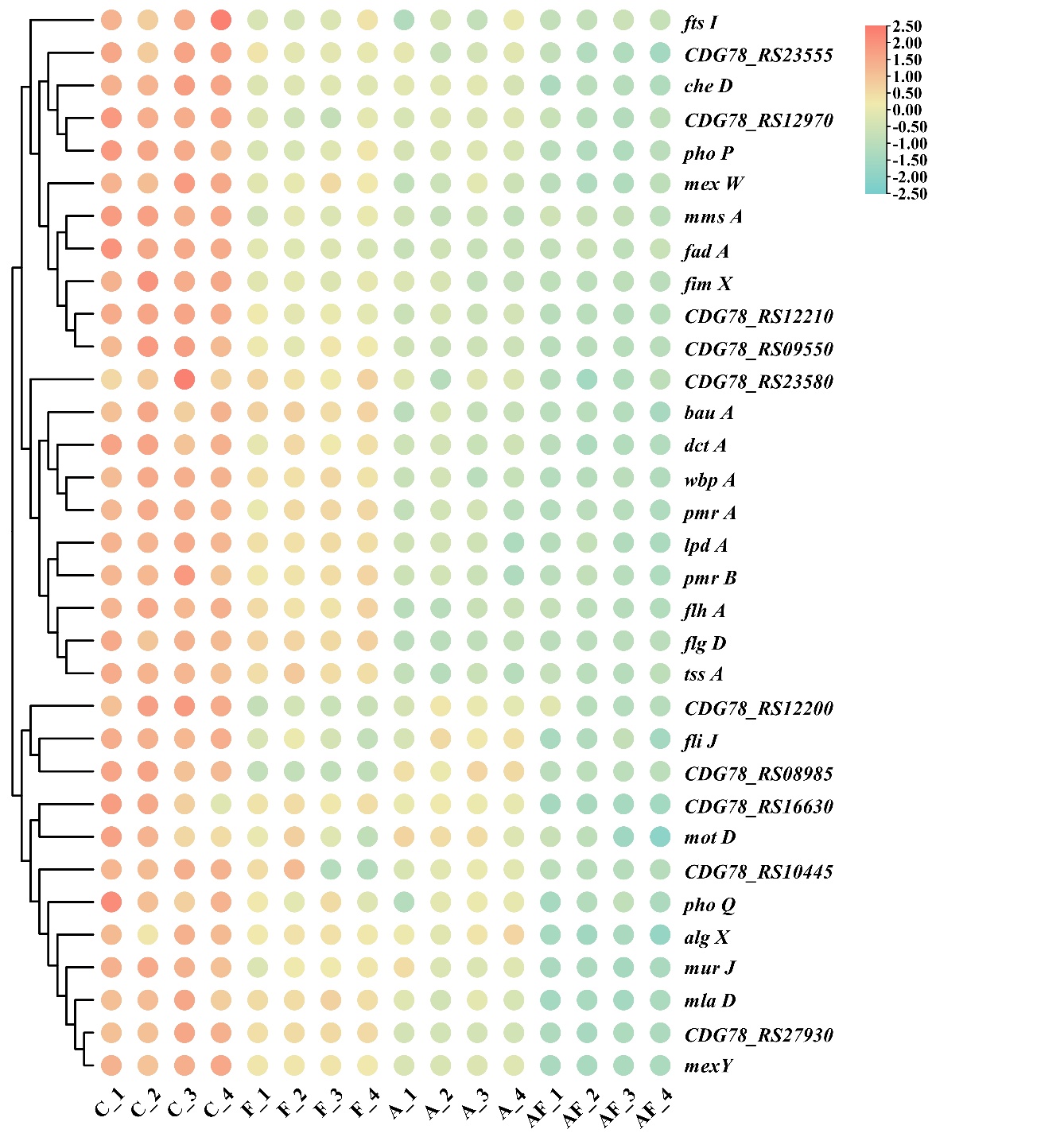
*

**Figure S29.** Heatmap of key genes expression levels.


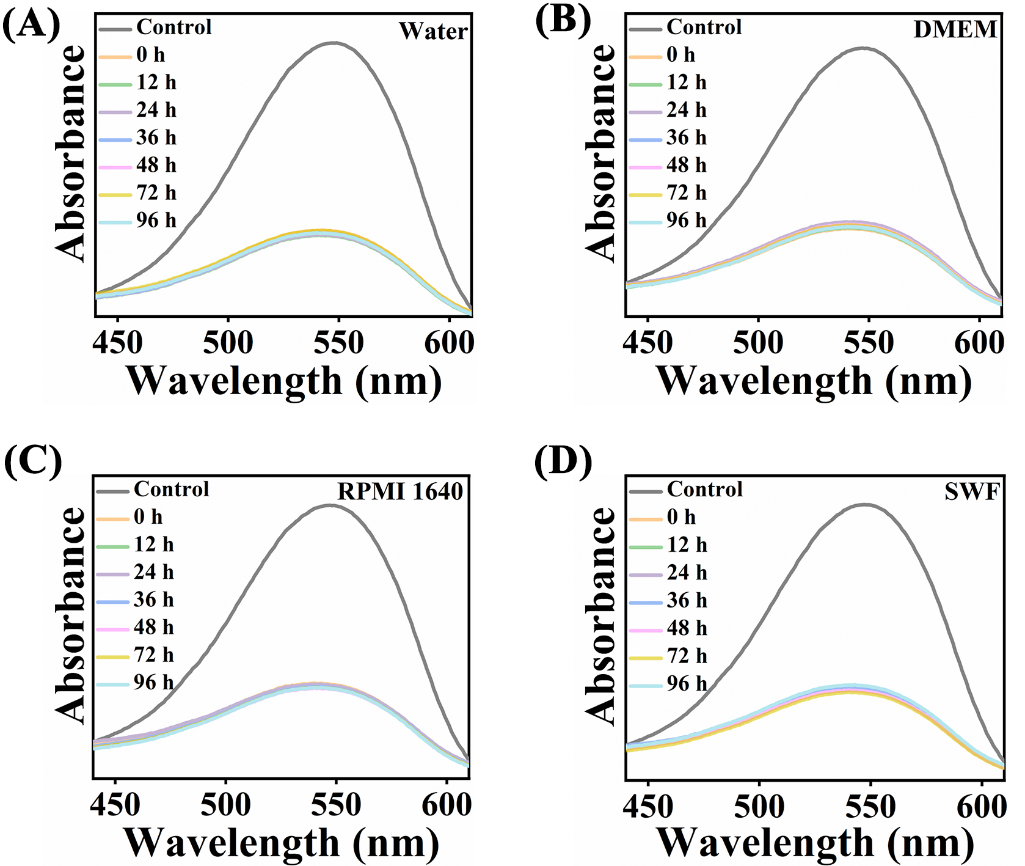


**Figure S30.** Superoxide (SOD)-like dismutase activity of AMCB in in water (A), DMEM (B), RPMI 1640 (C), and simulated wound fluid (SWF) (D) after different incubation time.


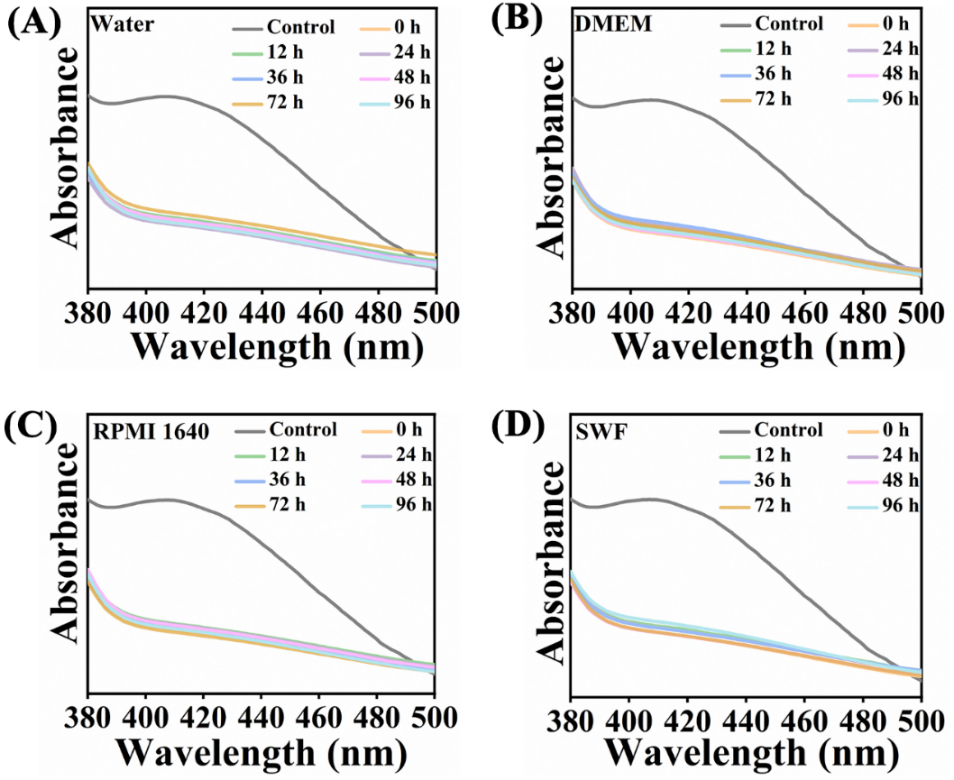


**Figure S31.** Catalase (CAT)-like activity of AMCB in water (A), DMEM (B), RPMI 1640 (C), and simulated wound fluid (SWF) (D) after different incubation time.





**Figure S32.** The NIH/3T3 cells viability after treatment with different concentration of AMCB for 24 h and 48 h was determined by MTT. Data are presented as mean ± SD (n = 6, independent measurements).





**Figure S33.** The RAW 264.7 cells viability after incubation with FTB and AMCB-FTB for 24 h and 48 h was determined by MTT assay. Data are presented as mean ± SD (n = 6, independent measurements).


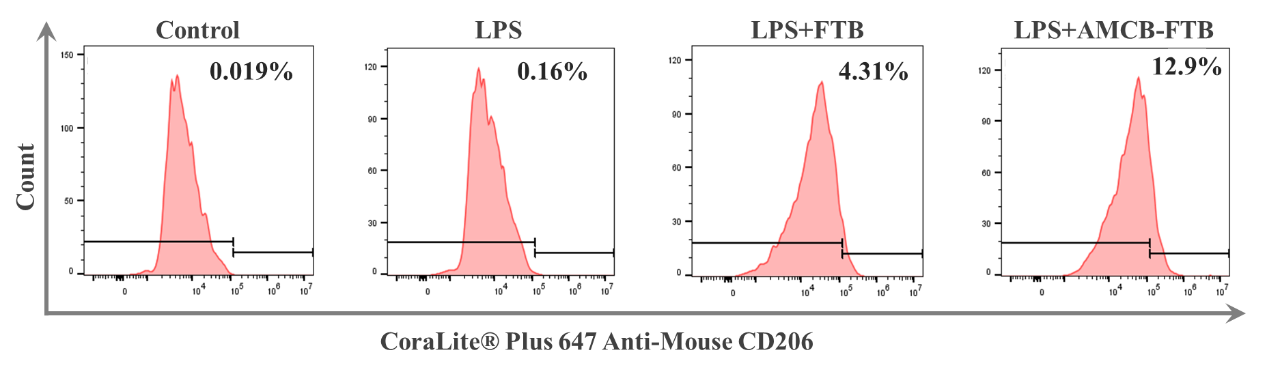


**Figure S34.** Flow cytometry detection of CD206 surface antigen ratio in RAW264.7 cells after different treatment.


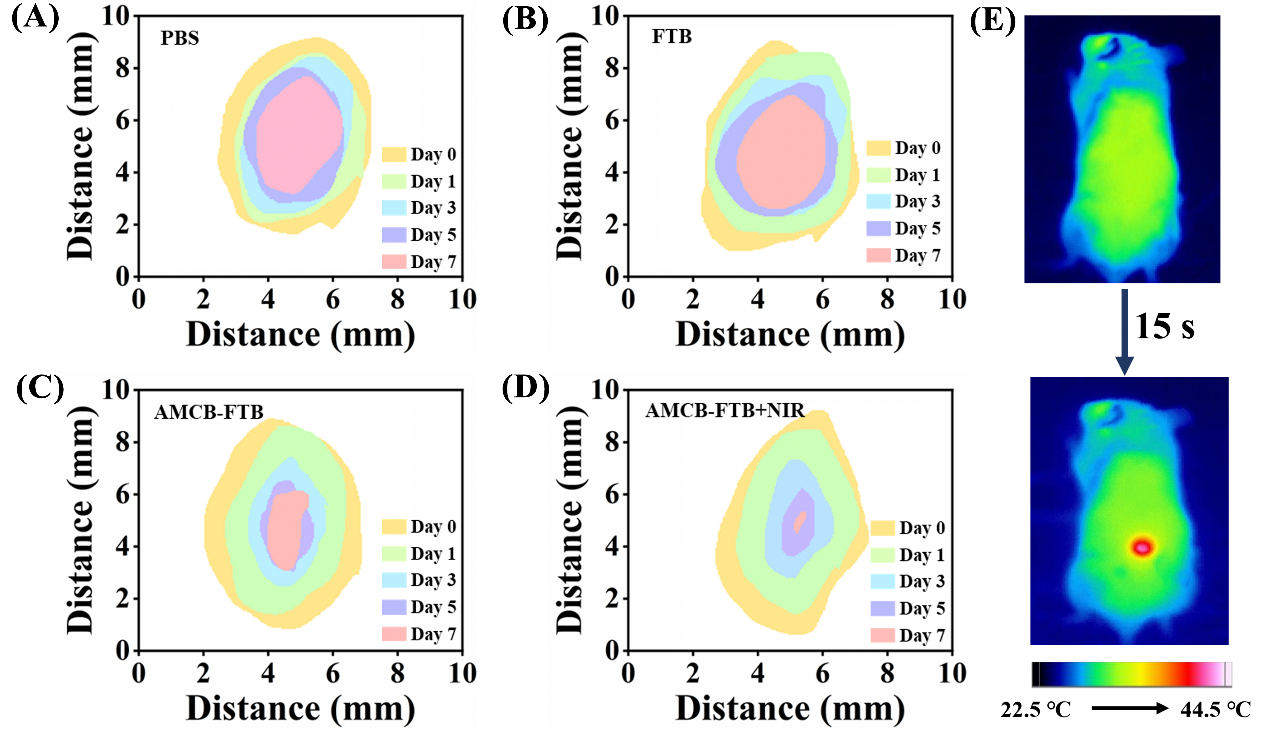


**Figure S35.** Traces of wound healing in PBS (A), FTB (B), AMCB-FTB (C) and AMCB-FTB + NIR (D). (E) The body temperature change of mice treated with AMCB-FTB under 808 nm.


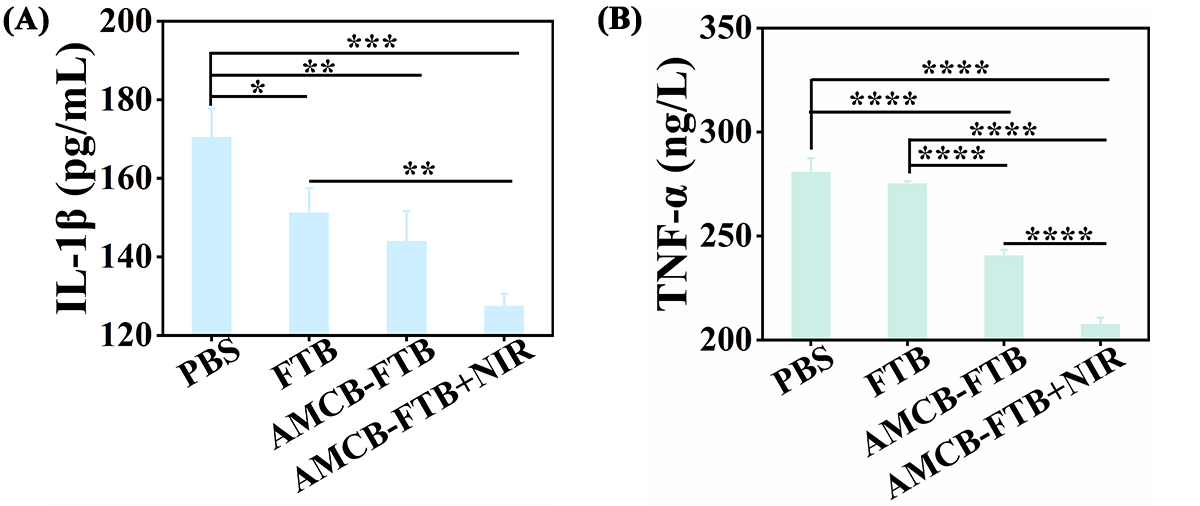


**Figure S36.** Changes in IL-1β (A) and TNF-α (B) levels in mouse serum after different treatment. Data are presented as mean ± SD (n = 3). Statistical significance was tested with one-way ANOVA, *p < 0.05, **p < 0.01, ***p < 0.001, ****p < 0.0001.


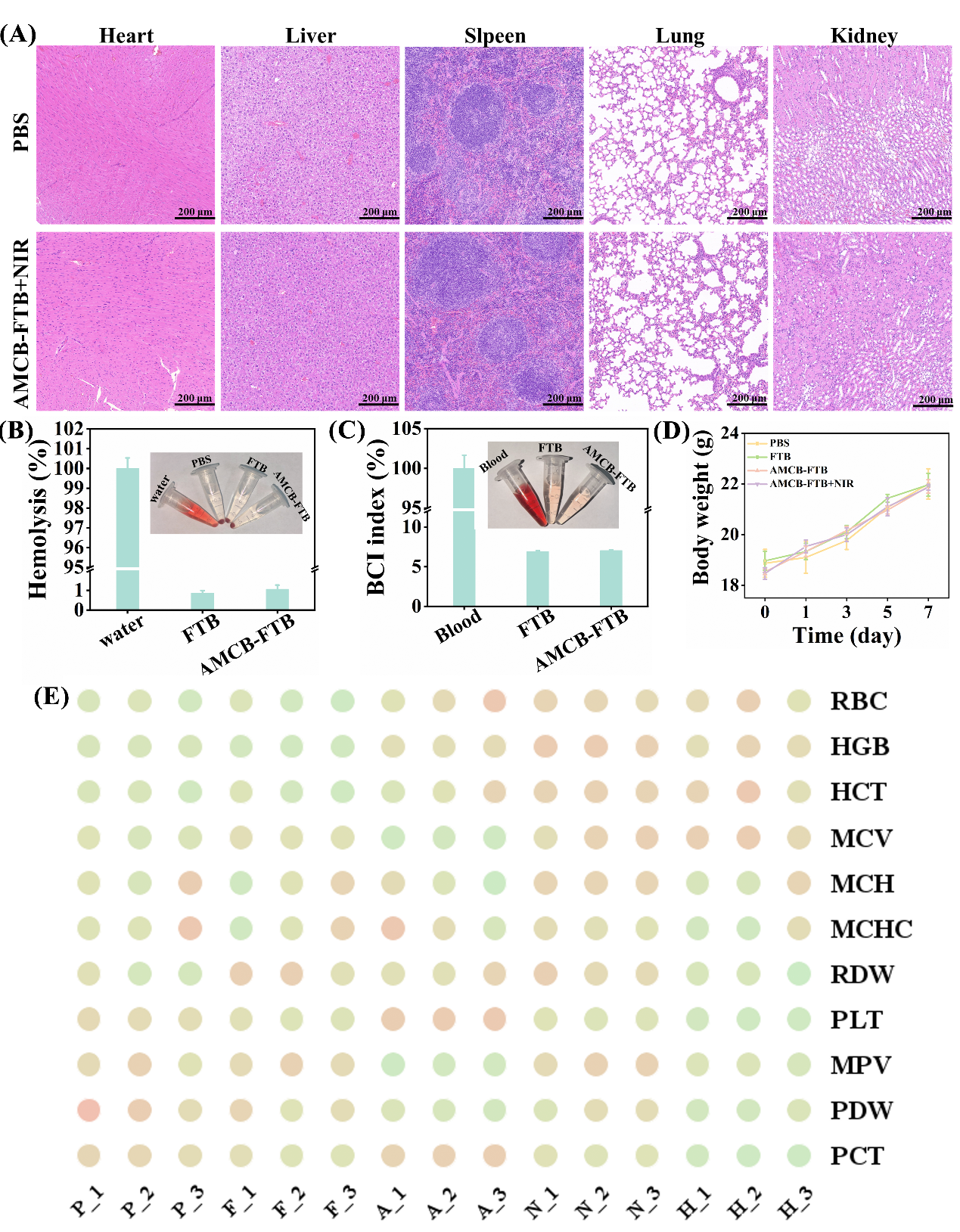


**Figure S37. *In vivo* biocompatibility assessment of AMCB-FTB**. (A) H&E sections of major organs (the heart, liver, spleen, lungs, and kidneys) of mice in different groups on day 7. (B) Blood compatibility evaluated by hemolysis assay. Data are presented as mean ± SD (n = 3, independent measurements). (C) Whole-blood clotting assay of FTB and AMCB-FTB. The inset is the corresponding photograph of the blood supernatants. Data are presented as mean ± SD (n = 3, independent measurements). (D) Body weights of the mice at days 0-7. Data are presented as mean ± SD (n = 6, independent measurements). (E) Routine blood of normal mice and other treatment groups on day 7, including red blood cell, hemoglobin, hematocrit, mean corpuscular volume, mean corpuscular hemoglobin concentration, mean corpuscular hemoglobin, red blood cell distribution width, mean platelet volume, platelet distribution width and thrombocytocrit (n = 3, independent measurements). P_1-P_3: I: Treatments with PBS. F_1-F_3: Treatments with FTB. A_1-A_3: Treatments with AMCB-FTB. N_1-N_3: Treatments with AMCB-FTB + NIR. H_1-H_3: Healthy mice.


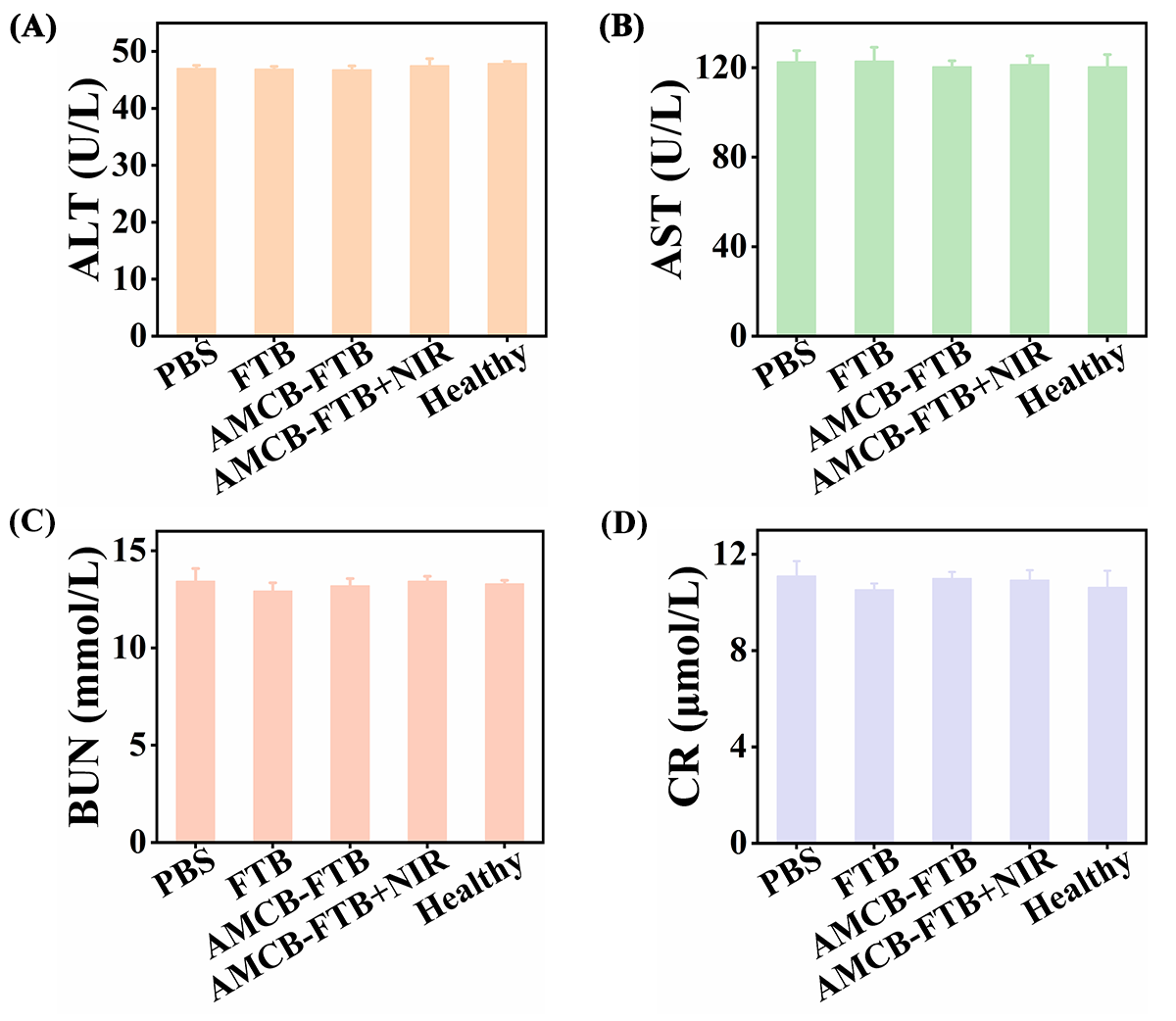


**Figure S38.** Changes in serum levels of alanine aminotransferase (ALT), aspartate aminotransferase (AST), blood urea nitrogen (BUN), and creatinine (CR) in mice after different treatment. Data are presented as mean ± SD (n = 3, independent measurements).

**Table S1.** Comparison of the kinetic constants for ROS-production activities of our synthesized materials with other reported metallic oxides and metal nanoparticles-based biocatalysts.

| **Materials** | ***K_m_* (mM)** | ***V_max_* (μM s^-1^)** | **TON (s^-1^)** | **References** |
| --- | --- | --- | --- | --- |
| AMCB | 0.15 | 0.629 | 9.54 | This work |
| Ru centers | 0.92 | 0.364 | 2.3 | *Adv. Mater.* 2024, 2408787 |
| Co centers | 0.09 | 0.034 | 0.165 |  |
| CoO | 92.1 | 1.14 | 8.55 | *Nat. Commun.* 2019, 10, 704 |
| CeO_2_ | 4.41 | 0.18 | 3.096 |  |
| Fe_2_O_3_ | 75.97 | 0.068 | 0.544 |  |
| Fe_3_O_4_ | 41.66 | 0.16 | 1.237 |  |
| CuO | 31.18 | 0.28 | 2.24 |  |
| Co_3_O_4_ | 41.75 | 0.26 | 2.089 |  |
| Mn_2_O_3_ | 12.53 | 1.01 | 7.979 |  |
| Cu(OH)_2_ | 28.91 | 0.34 | 3.332 |  |
| NiO | - | 0.011 | 0.082 |  |
| MnO_2_ | - | 0.006 | 0.056 |  |
| Mn_3_O_4_ | - | 0.013 | 0.099 |  |
| V_2_O_5_ | 9.88 | 0.49 | 2.57 | *Angew. Chem. Int. Ed.* 2023, 63, e202310811 |
| CuBCats | 1.789 | 0.565 | - | *Adv. Funct. Mater.* 2023, 33, 2301986 |
| c-RuS_2_ | 1.92 | 1.56 | 6.69 | *Small Methods,* 2023, 7, 2300011 |
| ZnO | 0.041 | 0.069 | 0.27 | *Adv. Mater.* 2022, 34, 2108646 |
| Pt NCs | - | 0.182 | 1.366 | *ACS Appl. Mater. Interfaces*, 2017, 9, 10027 |
| [PtFe@Fe_3_O_4_](mailto:PtFe@Fe3O4) | 53.55 | 0.108 | 8.617 | *Angew. Chem. Int. Ed.* 2019, 58, 12624 |

**Table S2.** Comparison of AMCB-FTB with other reported “photothermal-nanozyme” composite systems in terms of antibacterial efficiency, photothermal conversion efficiency, and enzymatic activity.

| **Photothermal-nanozymes** | **PCE** | **Enzyme-like activity** | **Antibacterial efficiency** | **References** |
| --- | --- | --- | --- | --- |
| AMCB-FTB | 46.5% | POD  CAT  SOD  GSH-Px | *P. aeruginosa:* 99.95%  *E. coli*: 99.97% | This work |
| Cu_7_S_4_ Nanozyme | - | POD  CAT  GSH-Px | *E. coli*: 80.42% | *Adv. Sci.* 2025, 12, e03793 |
| CuMnOx@CuO_2_@IR820 hydrogel | - | OXD  POD  CAT | *S. aureus,*  *E. coli,* MRSA: 99% | *Adv. Funct. Mater.* 2025, 35, 2421176 |
| Co_7_Fe_3_/ZnO@C | 29.1% | POD  SOD  CAT | Not specified | *Adv. Mater.* 2025, 37, 2418731 |
| Pt-Pd-Au alloy nanozymes | 52.21% | POD  CAT | MRSA:95% | *Bioact. Mater.* 2025, 51, 841-857 |
| PTPPG hydrogel | 30.1% | Gox  POD  CAT  OXD | *S. aureus,* MRSA,  *P. aeruginosa,* PDR-*PA*: >75% | *Adv. Sci.* 2025, 12, 2500696 |
| AuCeO_2_ hydrogel | - | GOx | Not specified | *Adv. Sci.* 2025, 12, 2414161 |
| Cu_2-x_Se nanoparticles (CSNPS) | 34.8% | POD  GSH-px | MRSA: 99.17%  *E. coli*: 99% | *Adv. Funct. Mater.* 2025, e07799 |
| Pd@Au nanoframe hydrogel | - | POD | *E. coli*: 98% | *ACS Nano,* 2025, 19, 15069-15080 |
| DAC@PDA/MnO_2_ | - | OXD | MRSA: >90% | *Chem. Eng. J*. 2025, 518, 164687 |
| BCSO NS | 31.6% | POD | MRSA: 100%  *E. coli*: 100% | *Nat. Commun.* 2025, 16, 5822 |
| mCu-SAE@BNN6@PEG-Van (CBPV) | 43.9% | POD  OXD | MRSA: 99.6% | *Adv. Sci.* 2025, e09621 |
| PDA-MnO_2_ bilayer microneedle patch | - | SOD  CAT | *E. coli:* 87.9%  *S. aureus:*95.5% | *Adv. Funct. Mater.* 2025, 35, 2417415 |

**Table S3.** Primers used for real-time RT-PCR of *P. aeruginosa* in this study.

| Primer name | Primer sequence (5′- 3′) |
| --- | --- |
| 16S-F | ACTCCTACGGGAGGCAG |
| 16S-R | ATTACCGCGGCTGCTGG |
| *cbrB*-F | CATCCCCTCCTTCGACATGG |
| *cbrB*-R | GCTCGGATTCGATCAGGGTT |
| *rsaL*-F | TCACACGAGAGAACACAGCC |
| *rsaL*-R | GATCTTGCCTCTCAGGTCGG |
| *fliL*-F | GCCTTGAGCGAACCAGTTTC |
| *fliL*-R | CAGACCGACGACGATGATGT |
| *flgG*-F | GTTCCAGGACCTGCTGTACC |
| *flgG*-R | CAGTTCCTCGACCACGTTGA |
| *prmA*-F | CGAGCACCATGTCGAGGAAA |
| *prmA*-R | GAGATCGAAGTGTGCGGAGT |
| *tagR*-F | CATCGGCAAGTACGAGGTGA |
| *tagR*-R | AGGACTCCTGGACCATCTCC |
| *prpB*-F | GCATATCGAGGACCAGGTCG |
| *prpB*-R | CTTCTGCTCGAAGCTGTGGT |
| *pilO2*-F | CCTCAAGCGCGAATACAAGC |
| *pilO2*-R | GATCTTGGGCTGCAGGTCTT |

**Table S4.** Primers used for real-time RT-PCR of *E. coli* in this study.

| Primer name | Primer sequence (5′- 3′) |
| --- | --- |
| 16S-F | AGGCCTTCGGGTTGTAAAGT |
| 16S-R | GTTAGCCGGTGCTTCTTCTG |
| *luxS*-F | CAATCACCGTGTTCGATCTGC |
| *luxS*-R | CGCATAAAACCAGCAAACAGG |
| *sdiA*-F | GATGACTTATTCAGCGAAGCACAG |
| s*diA*-R | GCAACGGGAAAAGGACAAAA |
| *phoP*-F | TGCGGGAAAGCCATACCATT |
| *phoP*-R | CGCGAACGGTGGTAATCACT |
| *rpoS*-F | CGAAAAAGCGTTGCTGGACA |
| *rpoS*-R | GCTCGAACAGCCATTTGACG |
| *marR*-F | CCTGGTCTGTAAAGGCTGGG |
| *marR*-R | GCAGGTCCTGGCCAACTAAT |
| *atpA*-F | TGTACTGGCCGTATCCTGGA |
| *atpA*-R | GGCTGATCTACGGACTGACG |
| *entA*-F | GACACCGATATGCAACGCAC |
| *entA*-R | GGTCAGAGGCGAGGAACAAA |

**Table S5.** Primers used for real-time RT-PCR of mouse and cells in this study.

| Primer name | Primer sequence (5′- 3′) |
| --- | --- |
| GAPDH-F | CTCCCACTCTTCCACCTTCG |
| GAPDHR | TTGCTGTAGCCGTATTCATT |
| TGF-β-F | ACCGCAACAACGCCATCTATGAG |
| TGF-β-R | GGCACTGCTTCCCGAATGTCTG |
| VEGF-F | GCACCCACGACAGAAGGAG |
| VEGF-R | GCATCAGCGGCACACAGGA |
| IL-1α-F | CTCCAGCTGGAGGAAGTTAAC |
| IL-1α-R | CTGACTCAAAGCTGGTGGTG |
| IL-10-F | GAGAAGCATGGCCCAGAAATC |
| IL-10-R | GAGAAATCGATGACAGCGCC |
| IL-6-F | ATAGTCCTTCCTACCCCAATTTCC |
| IL-6-R | GATGAATTGGATGGTCTTGGTCC |
| IL-4-F | CGTTGGAGGTCCGGTGTTTA |
| IL-4-R | CCACAAAACAGGCGAGTGTG |
| Arginase-F | CATATCTGCCAAGGACATCG |
| Arginase-R | GGTCTCTTCCATCACTTTGC |
| TNF-α-F | CTGAACTTCGGGGTGATCGG |
| TNF-α-R | GGCTTGTCACTCGAATTTTGAGA |
| HIF-1α-F | GGCGGCGAGAACGAGAAGAAAAATA |
| HIF-1α-R | GGAAGTGGCAACTGATGAGCAAG |
| INOS-F | ACATCGACCCGTCCACAGTAT |
| INOS-R | CAGAGGGGTAGGCTTGTCTC |
| CD68-F | TTCTTCTGCCTGGTGCTCAG |
| CD68-R | CTCTGGGTGGCAGATGTGAG |
| CD206-F | CCTGGAATGGGTAGGCCTTC |
| CD206-R | CACTGGTCTGCTGAAGCTCA |
| bFGF-F | ATGAAGGAAGATGGACGGCT |
| bFGF-R | TTCTGTCCAGGTCCCGTTTT |
| COL-Ⅲ-F | ACTGGTGAACGTGGCTCTAA |
| COL-Ⅲ-R | CGAGCCCTCAGATCCTCTTT |

1. Z. Xiao , J. Cao, J. Liu, T. Du, X. Du

   State Key Laboratory of Food Nutrition and Safety, College of Food Science and Engineering, Tianjin University of Science and Technology, Tianjin 300457, PR China

   E-mail: [tingdu@tust.edu.cn](mailto:tingdu@tust.edu.cn), xjdu@tust.edu.cn

   Z. Song

   College of Science, Huazhong Agricultural University, Wuhan 430070, PR China [↑](#footnote-ref-1)
